# Supplementary material for: Association between polygenic propensity for psychiatric disorders and nutrient intake
Source: Commun Biol. 2021 Aug 26;4:965. doi: 10.1038/s42003-021-02469-4 (PMC8390493; doi:10.1038/s42003-021-02469-4)
Supplement: Supplementary file 2 — Supplementary Information [file 42003_2021_2469_MOESM2_ESM.pdf]

Association between polygenic propensity for psychiatric disorders and nutrient intake

Avina K. Hunjan, Christopher Hübel, Yuhao Lin, Thalia C. Eley, Gerome Breen.

Supplementary Methods

Recruitment and completion of dietary assessment

UK Biobank investigators sent postal invitations to 9,238,453 individuals registered with the UK's National Health Service who lived within approximately 25 miles (40 km) of one of 22 assessment centers located throughout England, Wales, and Scotland. Overall, 503,317 participants consented to join the study cohort and visited an assessment center between 2006 and 2010, resulting in a participation rate of 5.45 %.

Participants recruited between April 2009 and September 2010 completed the dietary assessment at the assessment centre. After the recruitment period closed, the dietary assessment was completed remotely on four separate occasions (cycle 1, February-April 2011; cycle 2, June-September 2011; cycle 3, October-December 2011; cycle 4, April-June 2012) by participants who provided an email address at recruitment. In total, 211,036 participants completed the dietary assessment at least once. Given that follow-up assessments were optional, this explains why not all participants recruited by the UK Biobank project completed the diet assessment at least once. Nonetheless, the sample size used in our study was sufficiently large.

Calculation of nutrient intakes

Nutrient intake (Category 100098) was pre-calculated by UK Biobank using a three-step process. First, each food and drink type listed in the dietary assessment tool was assigned a portion size, using the 'Food Portion Sizes' atlas [1]. Secondly, the quantity of each food and drink consumed was calculated by multiplying the assigned portion size by the amount

consumed. Using this information, nutrient intake for each participant was calculated by multiplying the quantity consumed by the nutrient composition of the food or drink, as taken from a food composition database [2].

Matrix decomposition to identify number of independent tests

We used a Bonferroni threshold (by correcting α as α/N) to estimate the number of significant associations, with N = estimated number of independent tests. We built a similarity matrix reflecting the trait similarity where D = number of principal components accounting for 99.5% of the data variance in the correlation matrix. In that case, D is the estimated number of independent traits, and the number of independent tests can be computed as $N = (D(D-1))/2$.

Supplementary Figures

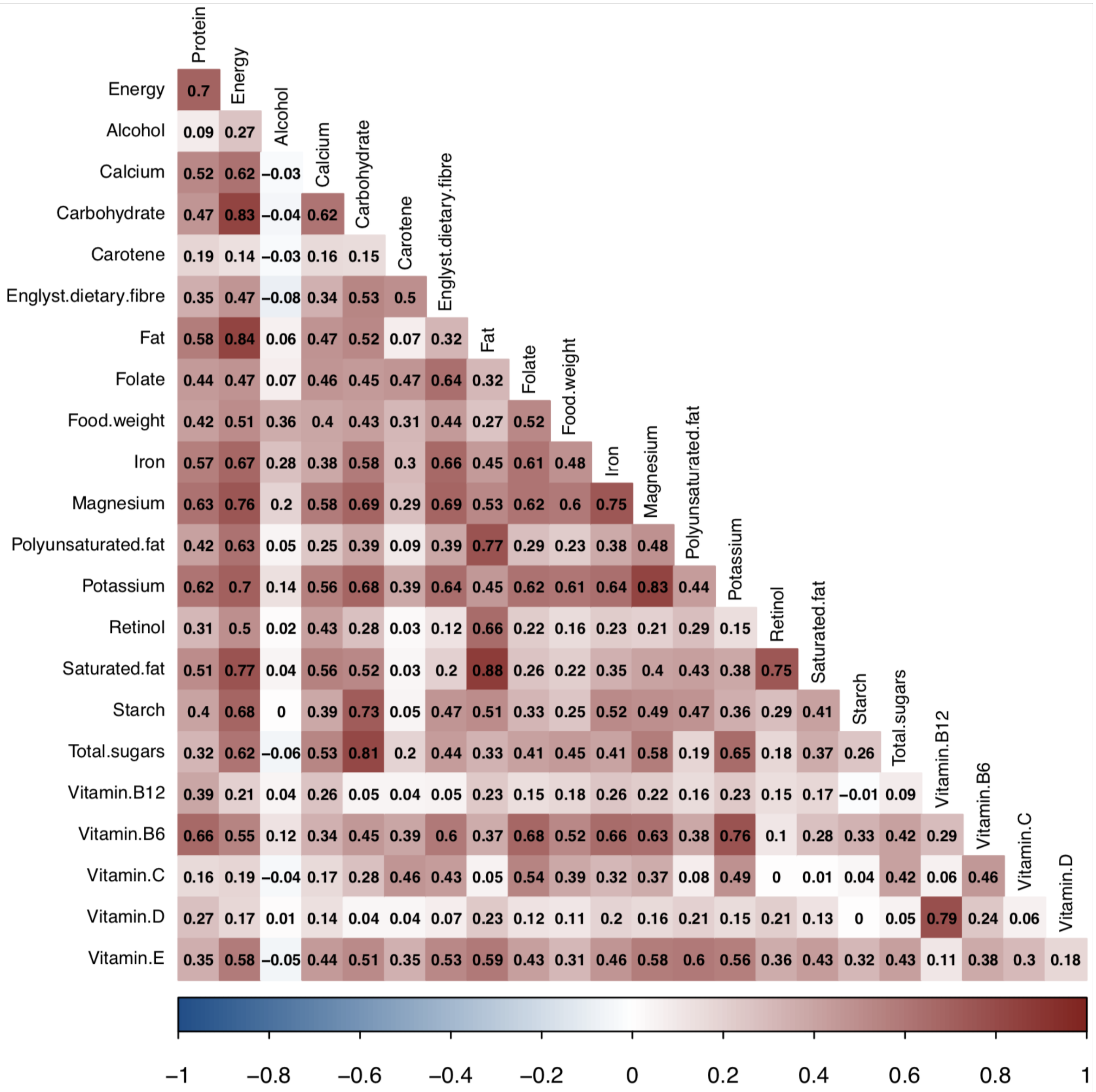

Supplementary Figure 1. Correlation matrix of nutrients. For each participant all time points were included for each nutrient. Data are represented for 163,619 participants of the UK Biobank cohort.

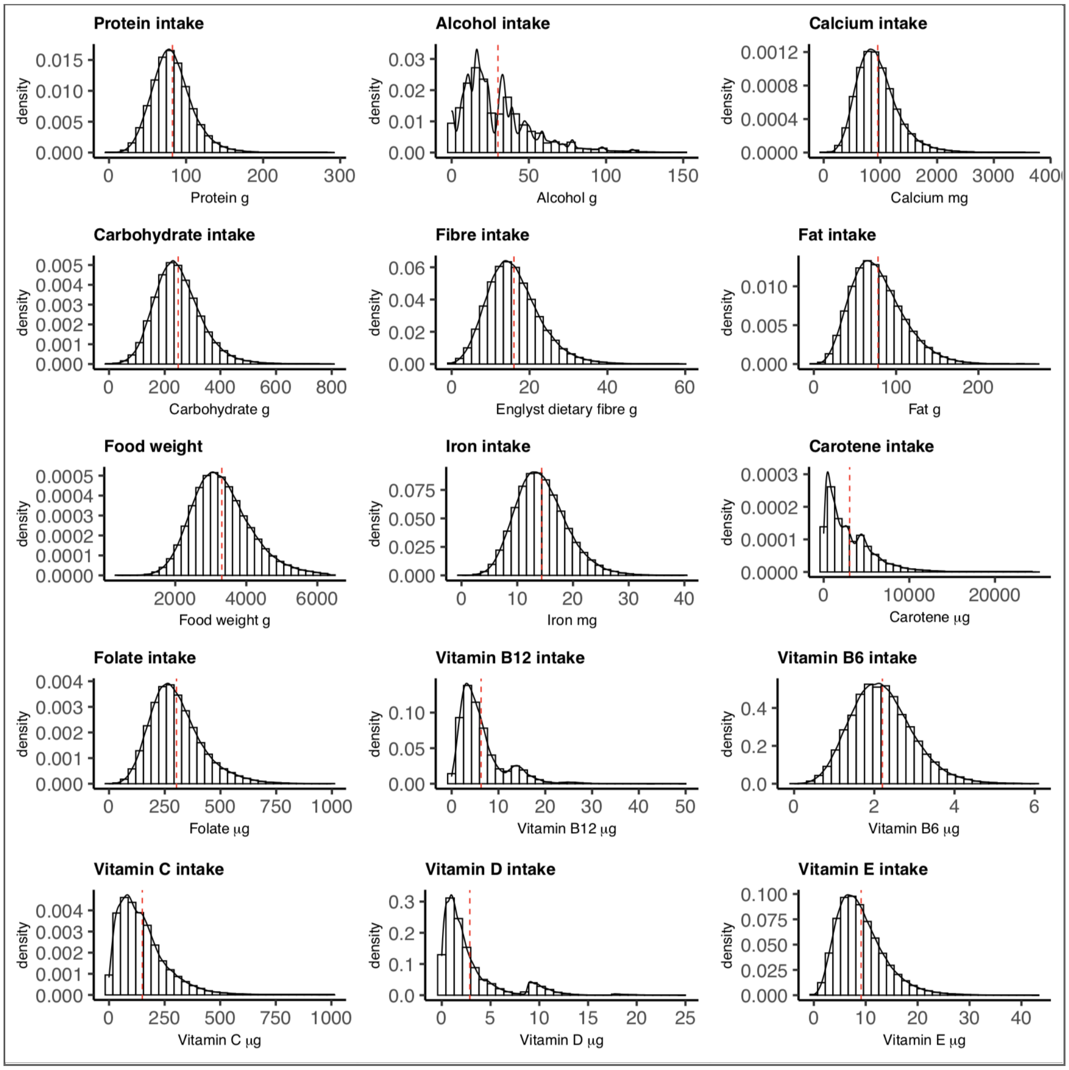

Supplementary Figure 2. Histogram plots of each nutrient with a fitted normal density curve. For each participant all time points were plotted for each nutrient. The red dashed line represents the mean. Data are represented for 163,619 participants of the UK Biobank cohort.

A

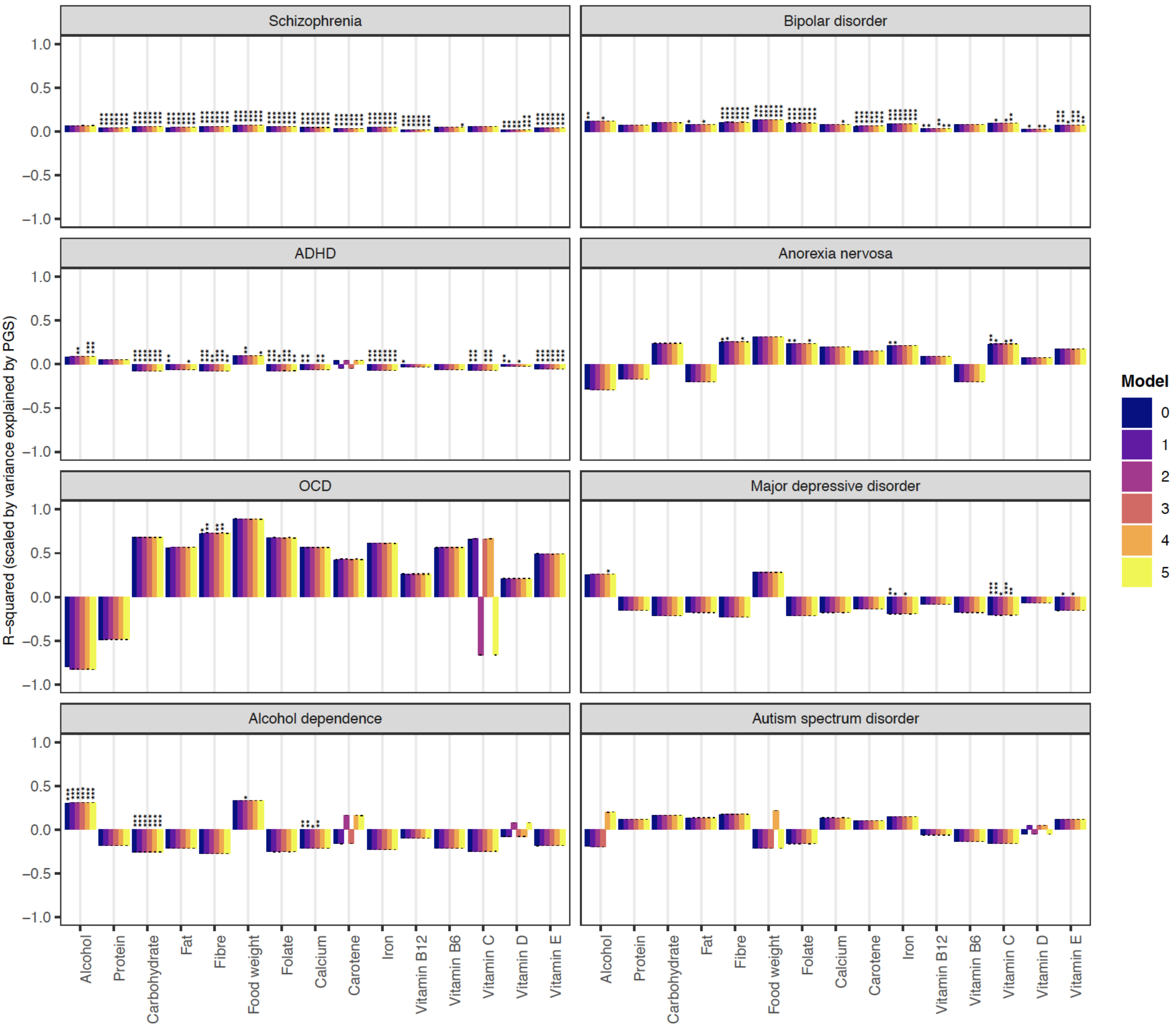

Schizophrenia
Bipolar disorder
ADHD
Anorexia nervosa
OCD
Major depressive disorder
Alcohol dependence
Autism spectrum disorder
R−squared (scaled by variance explained by PGS)
1.0
0.5
0.0
−0.5
−1.0
Alcohol
Protein
Carbohydrate
Fat
Fibre
Food weight
Folate
Calcium
Carotene
Iron
Vitamin B12
Vitamin B6
Vitamin C
Vitamin D
Vitamin E
Model
0
1
2
3
4
5

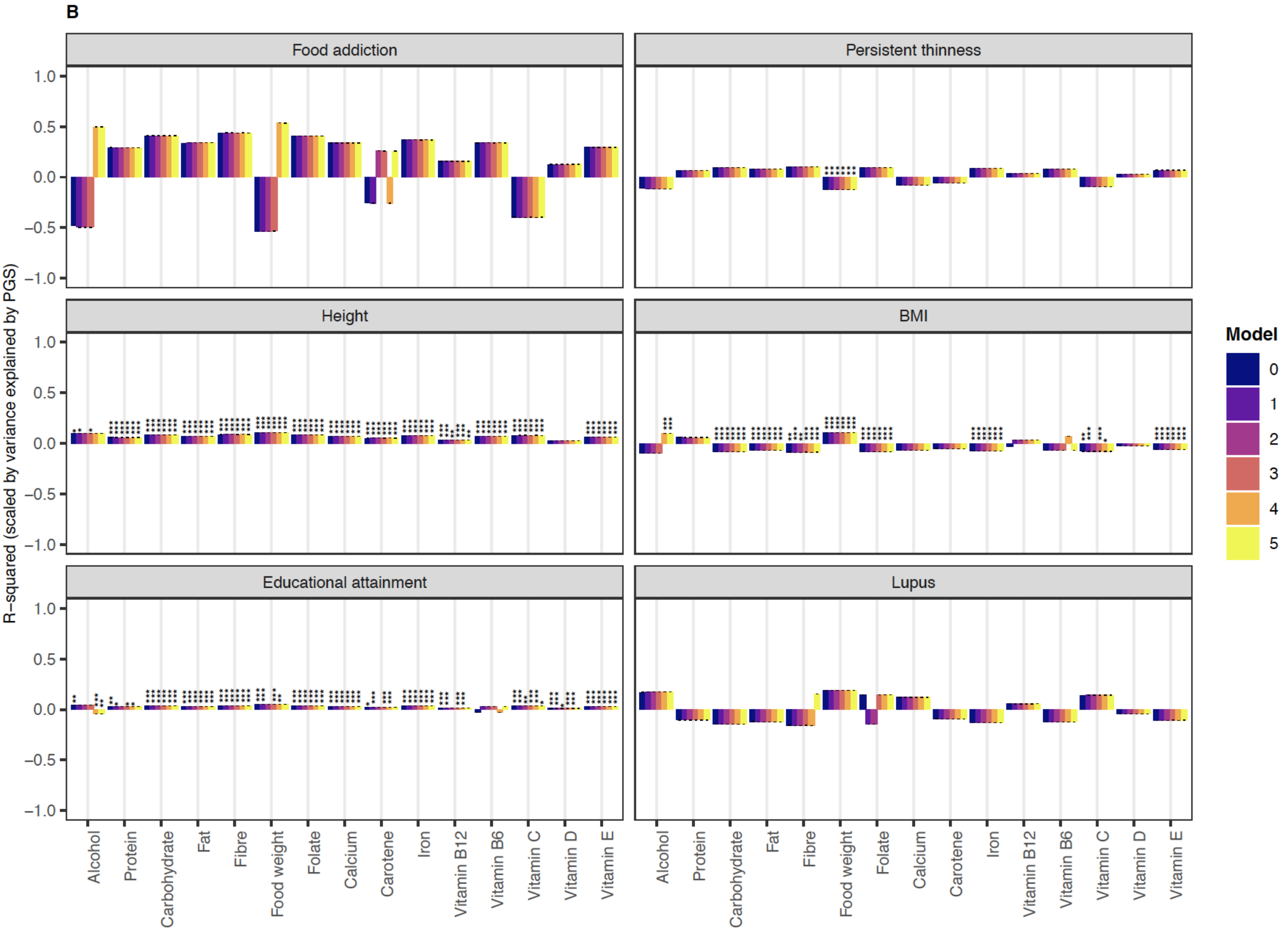

Supplementary Figure 3. Scaled associations between polygenic scores for psychiatric, behavioural and anthropometric traits and nutrient intake - configuration 1 from Table 2. A) psychiatric disorders and B) psychiatric-related traits, height, BMI, educational attainment and lupus.

Results are shown from linear mixed-effects model analyses. Y axis shows the R-squared estimates which have been scaled by the variance explained by the PGS predicting itself on the liability scale and have been multiplied by the direction of the coefficient estimate. Colours represent the different models:

Model 0: Sex, age and PC 1-6

Model 1: Sex, age, PC 1-6, special diet and typical diet yesterday

Model 2: Model 1 + socioeconomic status and educational attainment

Model 3: Model 1 + Physical activity

Model 4: Model 1 + diagnoses and medication that affect food intake, smoking and alcohol consumption

Model 5: All fixed effects

Error bars represent standard errors and asterisks indicate statistically significant estimates.

Bonferroni-corrected p value thresholds: * = $p < 0.05/132$, ** = $p < 0.01/132$, *** = $p < 0.001/132$

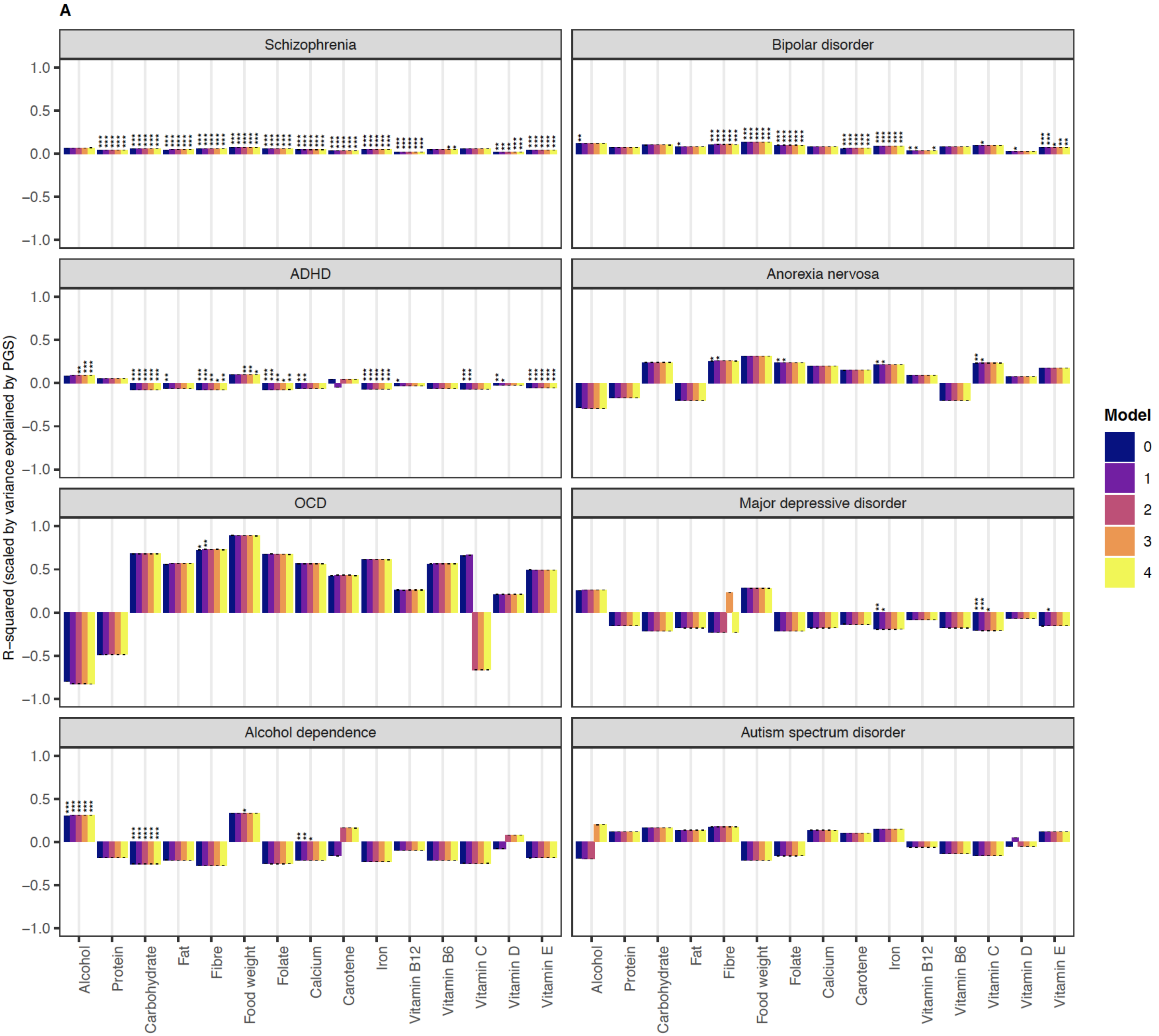
A
Schizophrenia
Bipolar disorder
ADHD
Anorexia nervosa
OCD
Major depressive disorder
Alcohol dependence
Autism spectrum disorder
R−squared (scaled by variance explained by PGS)
1.0
0.5
0.0
−0.5
−1.0
Alcohol
Protein
Carbohydrate
Fat
Fibre
Food weight
Folate
Calcium
Carotene
Iron
Vitamin B12
Vitamin B6
Vitamin C
Vitamin D
Vitamin E
Model
0
1
2
3
4

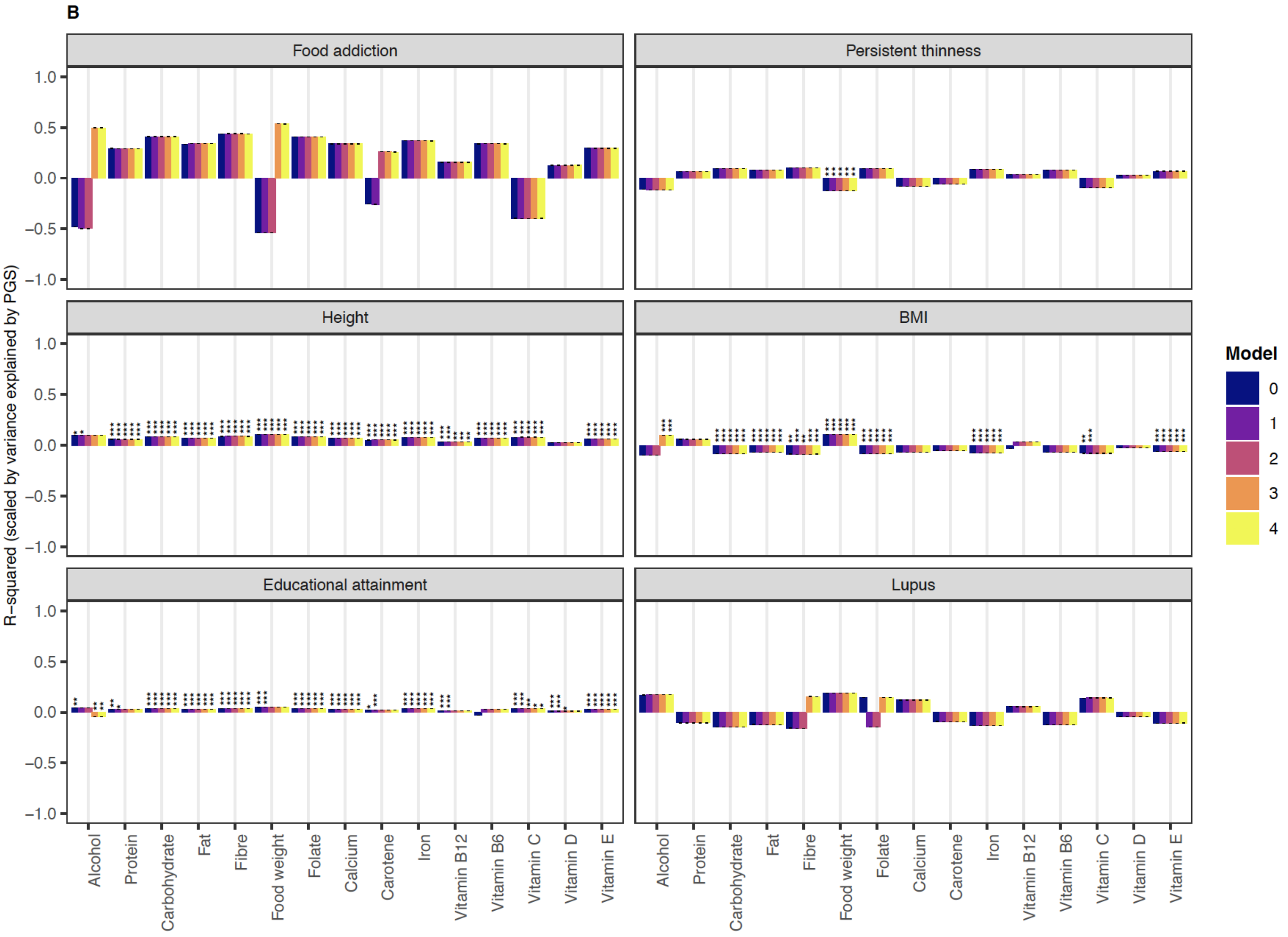

Supplementary Figure 4. Scaled associations between polygenic scores for psychiatric, behavioural and anthropometric traits and nutrient intake - configuration 2 from Table 2. A) psychiatric disorders and B) psychiatric-related traits, height, BMI, educational attainment and lupus.

Results are shown from linear mixed-effects model analyses. Y axis shows the R-squared estimates which have been scaled by the variance explained by the PGS predicting itself on the liability scale and have been multiplied by the direction of the coefficient estimate. Colours represent the different models:

Model 0: Sex, age and PC 1-6

Model 1: Sex, age, PC 1-6, special diet and typical diet yesterday

Model 2: Model 1 + socioeconomic status and educational attainment

Model 3: Model 2 + diagnoses and medication that affect food intake, smoking and alcohol consumption
Model 4: Model 3 + physical activity
Error bars represent standard errors and asterisks indicate statistically significant estimates.
Bonferroni-corrected p value thresholds: * = $p < 0.05/132$, ** = $p < 0.01/132$, *** = $p < 0.001/132$

A

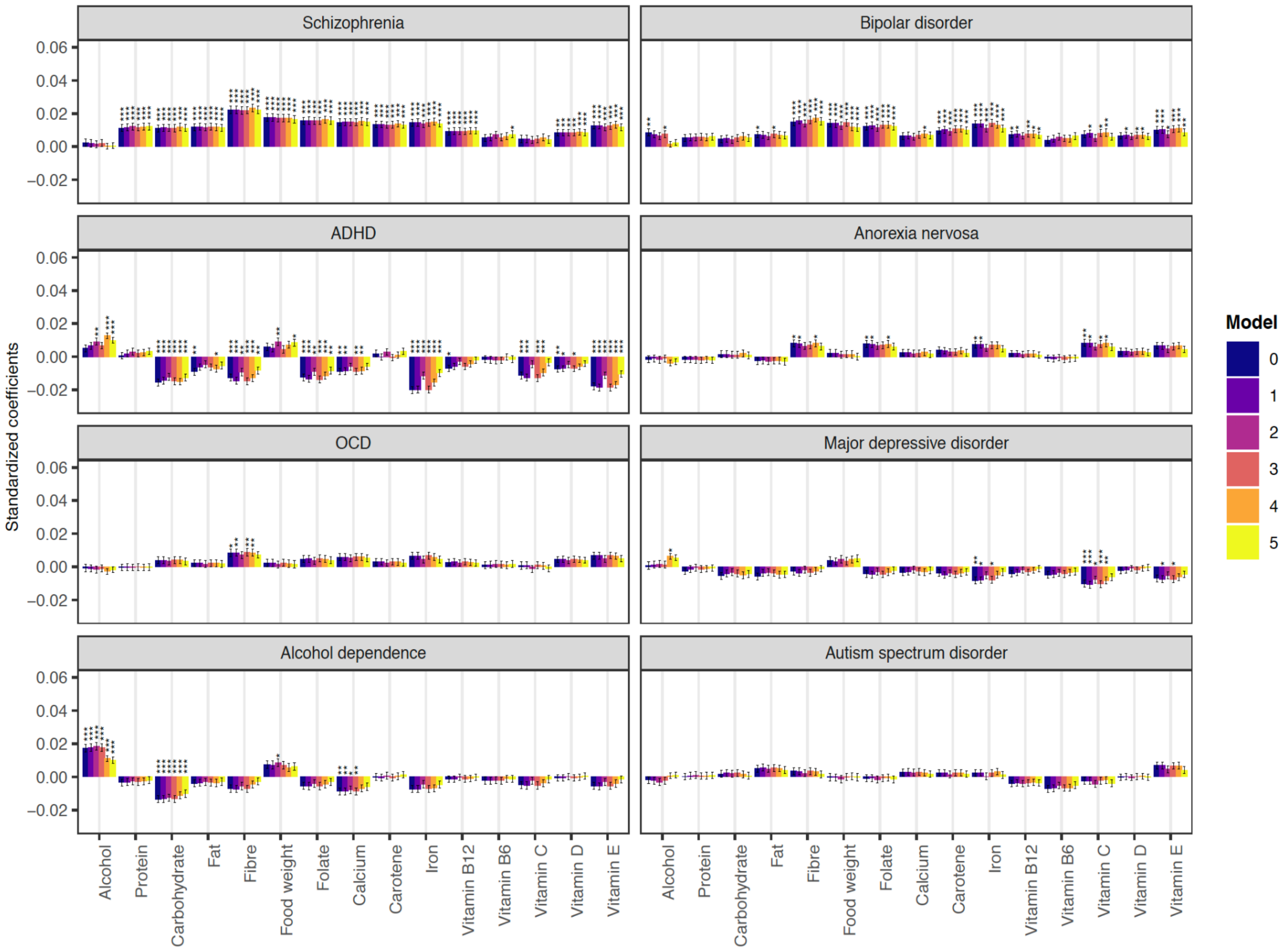

Schizophrenia
Bipolar disorder
ADHD
Anorexia nervosa
OCD
Major depressive disorder
Alcohol dependence
Autism spectrum disorder
Standardized coefficients
0.06
0.04
0.02
0.00
−0.02
Alcohol
Protein
Carbohydrate
Fat
Fibre
Food weight
Folate
Calcium
Carotene
Iron
Vitamin B12
Vitamin B6
Vitamin C
Vitamin D
Vitamin E
Model
0
1
2
3
4
5

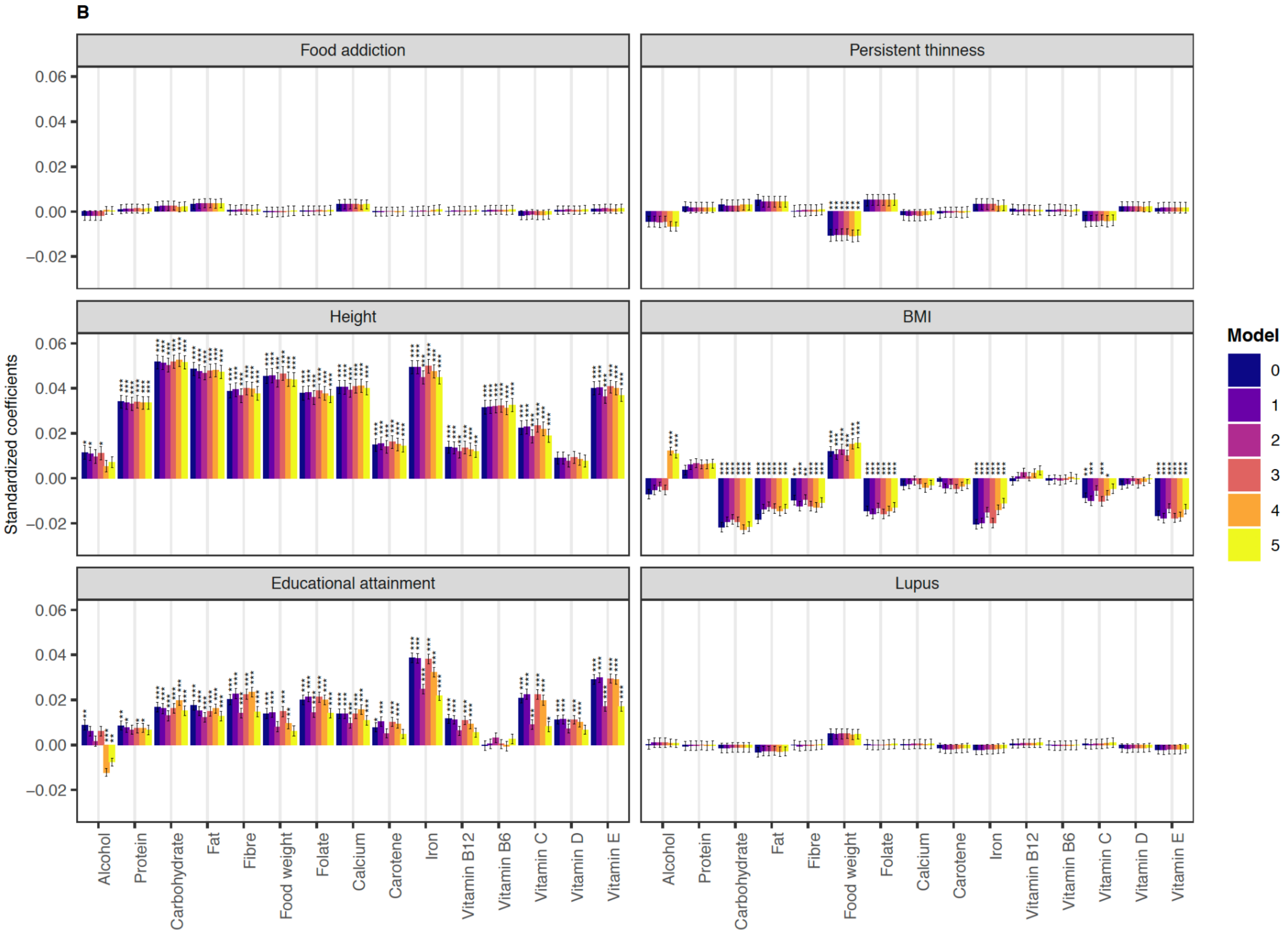

Supplementary Figure 5. Unscaled associations between polygenic scores for psychiatric, behavioural and anthropometric traits and nutrient intake - configuration 1 from Table 2. A) psychiatric disorders and B) psychiatric-related traits, height, BMI, educational attainment and lupus.

Results are shown from linear mixed-effects model analyses. Y axis shows the standardised β coefficient estimate (measured in units of standard deviation). PGSs and nutrient intakes were standardised with the figure showing the effect of a one standard deviation increase in PGS on nutrient intake. Colours represent the different models:

Model 0: Sex, age and PC 1-6

Model 1: Sex, age, PC 1-6, special diet and typical diet yesterday

Model 2: Model 1 + socioeconomic status and educational attainment

Model 3: Model 1 + Physical activity
Model 4: Model 1 + diagnoses and medication that affect food intake, smoking and alcohol consumption
Model 5: All fixed effects
Error bars represent standard errors and asterisks indicate statistically significant estimates.
Bonferroni-corrected p value thresholds: * = $p < 0.05/132$, ** = $p < 0.01/132$, *** = $p < 0.001/132$

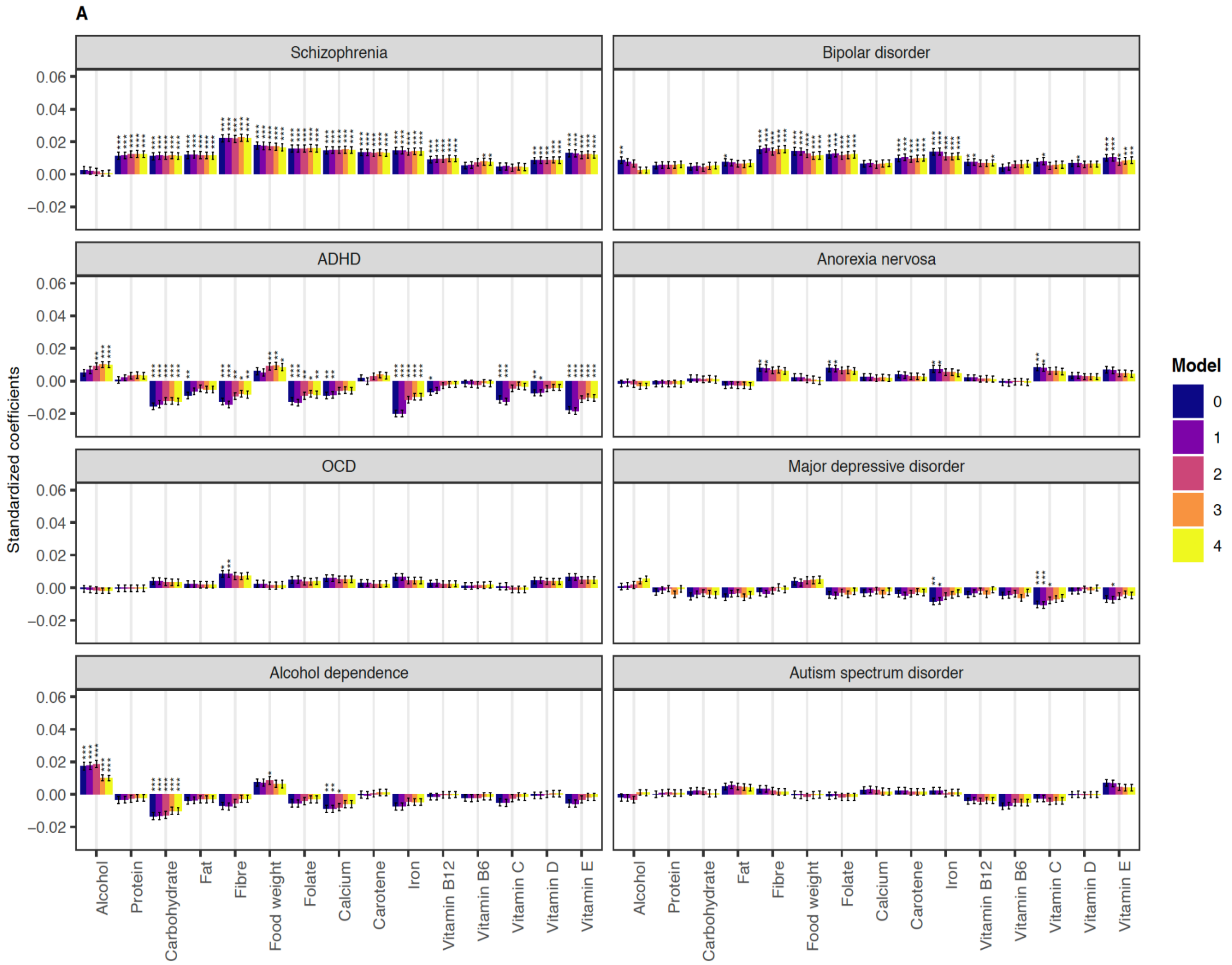
A
Schizophrenia
Bipolar disorder
ADHD
Anorexia nervosa
OCD
Major depressive disorder
Alcohol dependence
Autism spectrum disorder
Standardized coefficients
0.06
0.04
0.02
0.00
−0.02
Alcohol
Protein
Carbohydrate
Fat
Fibre
Food weight
Folate
Calcium
Carotene
Iron
Vitamin B12
Vitamin B6
Vitamin C
Vitamin D
Vitamin E
Model
0
1
2
3
4

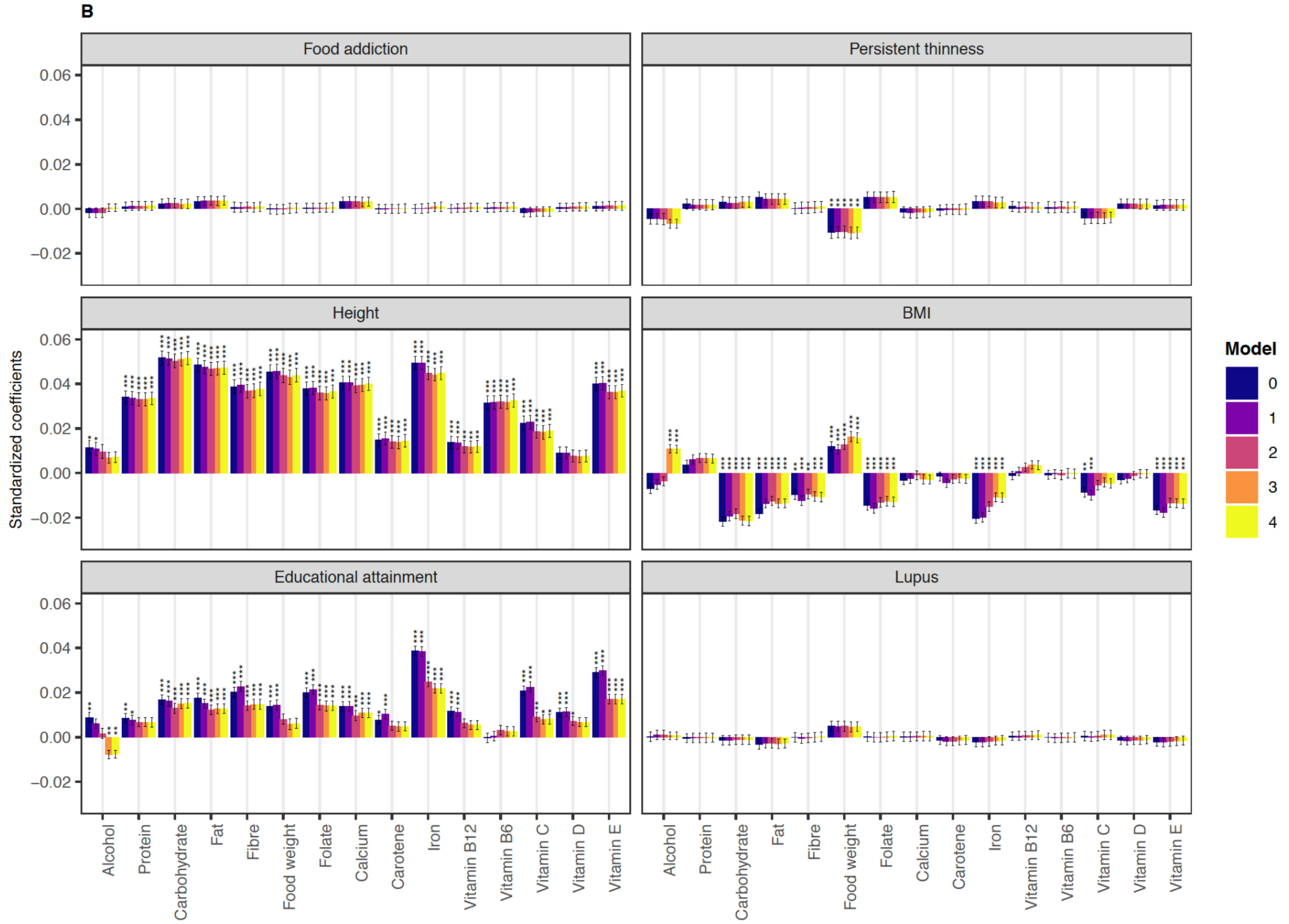

Supplementary Figure 6. Unscaled associations between polygenic scores for psychiatric, behavioural and anthropometric traits and nutrient intake - configuration 2 from Table 2. A) psychiatric disorders and B) psychiatric-related traits, height, BMI, educational attainment and lupus.

Results are shown from linear mixed-effects model analyses. Y axis shows the standardised β coefficient estimate (measured in units of standard deviation). PGSs and nutrient intakes were standardised with the figure showing the effect of a one standard deviation increase in PGS on nutrient intake. Colours represent the different models:

Model 0: Sex, age and PC 1-6

Model 1: Sex, age, PC 1-6, special diet and typical diet yesterday

Model 2: Model 1 + socioeconomic status and educational attainment
Model 3: Model 2 + diagnoses and medication that affect food intake, smoking and alcohol consumption
Model 4: Model 3 + physical activity
Error bars represent standard errors and asterisks indicate statistically significant estimates.
Bonferroni-corrected p value thresholds: * = $p < 0.05/132$, ** = $p < 0.01/132$, *** = $p < 0.001/132$

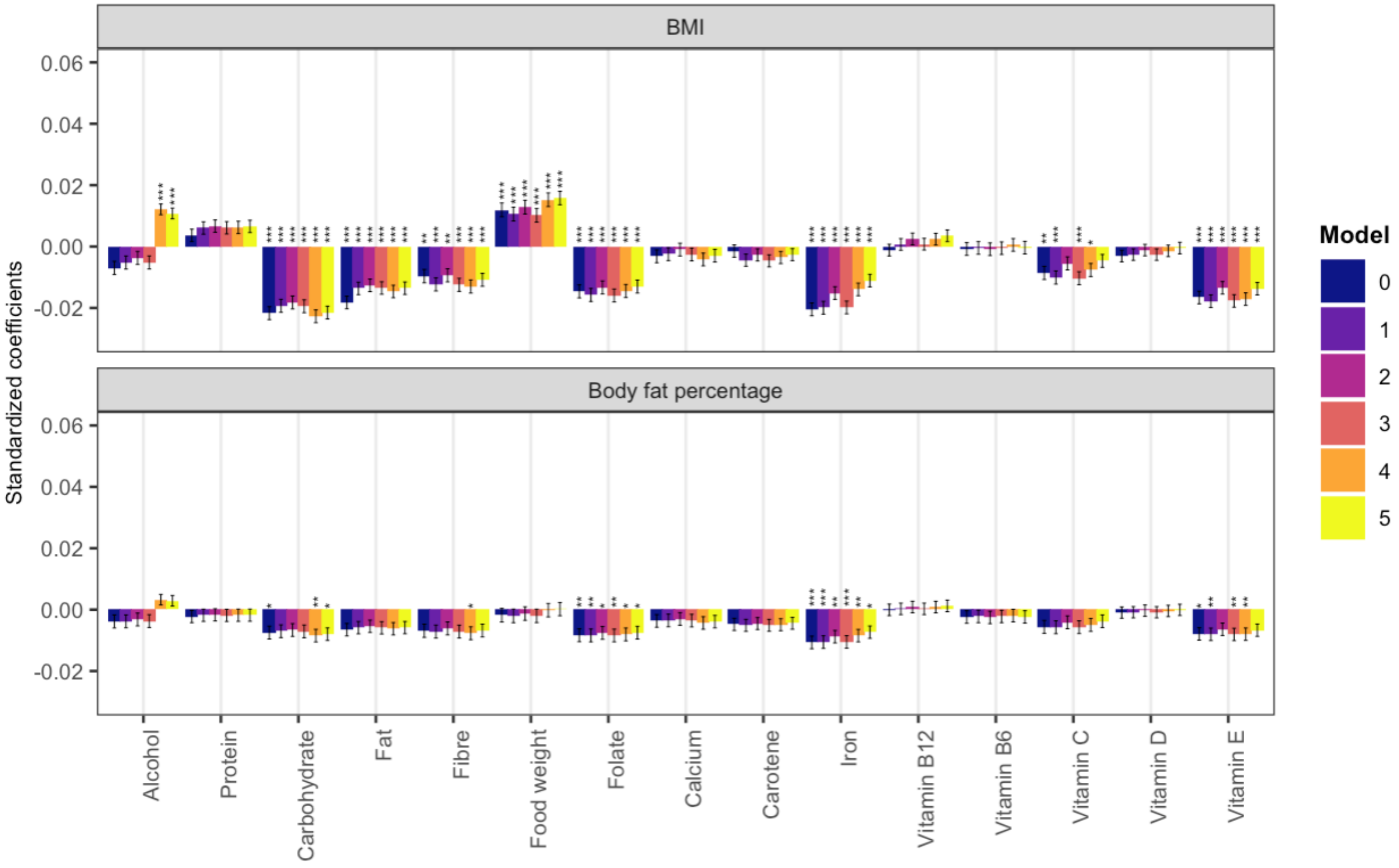

Supplementary Figure 7. Unscaled associations between polygenic scores for BMI and body fat percentage and nutrient intake.

Results are shown from linear mixed-effects model analyses. Y axis shows the standardised β coefficient estimate (measured in units of standard deviation). PGSs and nutrient intakes were standardised with the figure showing the effect of a one standard deviation increase in PGS on nutrient intake. Colours represent the different models:

Model 0: Sex, age and PC 1-6

Model 1: Sex, age, PC 1-6, special diet and typical diet yesterday

Model 2: Model 1 + socioeconomic status and educational attainment

Model 3: Model 1 + Physical activity

Model 4: Model 1 + diagnoses and medication that affect food intake, smoking and alcohol consumption

Model 5: All fixed effects

Error bars represent standard errors and asterisks indicate statistically significant estimates. Bonferroni-corrected p value thresholds: * = $p < 0.05/132$, ** = $p < 0.01/132$, *** = $p < 0.001/132$

Supplementary References

1. Crawley, H., Mills, A., Patel, S., Great Britain. & Ministry of Agriculture, Fisheries and Food. *Food portion sizes*. (H.M.S.O., 1993).
2. Roe, M., Pinchen, H., Church, S. & Finglas, P. McCance and Widdowson's The Composition of Foods Seventh Summary Edition and updated Composition of Foods Integrated Dataset : McCance and Widdowson's The Composition of Foods Seventh Summary Edition. *Nutr. Bull.* **40**, 36–39 (2015).
