# Supplementary material for: Association between polygenic propensity for psychiatric disorders and nutrient intake
Source: Commun Biol. 2021 Aug 26;4:965. doi: 10.1038/s42003-021-02469-4 (PMC8390493; doi:10.1038/s42003-021-02469-4)
Supplement: Supplementary file 3 — Description of Additional Supplementary Files [file 42003_2021_2469_MOESM3_ESM.pdf]

Description of Additional Supplementary Files

File name: Supplementary Data 1

Description: Table summarising the associations between polygenic scores for psychiatric traits and nutrient intake. Raw data for Supplementary Figure 1

Model 0: Sex, age and PC 1-6

Model 1: Sex, age, PC 1-6, special diet and typical diet yesterday

Model 2: Model 1 + socioeconomic status and educational attainment

Model 3: Model 1 + Physical activity

Model 4: Model 1 + diagnoses and medication that affect food intake, smoking and alcohol consumption

Model 5: All fixed effects

File name: Supplementary Data 2

Description: Table summarising the associations between polygenic scores for psychiatric traits and nutrient intake. Raw data for Supplementary Figure 2

Model 0: Sex, age and PC 1-6

Model 1: Sex, age, PC 1-6, special diet and typical diet yesterday

Model 2: Model 1 + socioeconomic status and educational attainment

Model 3: Model 2 + diagnoses and medication that affect food intake, smoking and alcohol consumption

Model 4: Model 3 + physical activity
