# Supplementary material for: Association between polygenic propensity for psychiatric disorders and nutrient intake
Source: Commun Biol. 2021 Aug 26;4:965. doi: 10.1038/s42003-021-02469-4 (PMC8390493; doi:10.1038/s42003-021-02469-4)
Supplement: Supplementary file 4 — Supplementary Data 1 [file 42003_2021_2469_MOESM4_ESM.pdf]

PRS	Nutrient Intake	Model	Estimate	SE	Standardised Estimate	Standardised SE	R-squared	R-squared SE	P-value
ADHD	Alcohol	0	0.1139	0.0483	0.0050	0.0021	0.4792	0.0020	0.0183
ADHD	Alcohol	1	0.1523	0.0477	0.0067	0.0021	0.4964	0.0019	0.0014
ADHD	Alcohol	2	0.2068	0.0478	0.0091	0.0021	0.4966	0.0019	0.0000
ADHD	Alcohol	3	0.1533	0.0477	0.0067	0.0021	0.4973	0.0019	0.0013
ADHD	Alcohol	4	0.2894	0.0393	0.0127	0.0017	0.4962	0.0020	0.0000
ADHD	Alcohol	5	0.2267	0.0393	0.0100	0.0017	0.4971	0.0020	0.0000
ADHD	Calcium	0	-3.4487	0.7972	-0.0089	0.0021	0.3396	0.0022	0.0000
ADHD	Calcium	1	-3.3511	0.7970	-0.0087	0.0021	0.3396	0.0022	0.0000
ADHD	Calcium	2	-2.3049	0.7988	-0.0060	0.0021	0.3395	0.0022	0.0039
ADHD	Calcium	3	-3.4357	0.7941	-0.0089	0.0021	0.3393	0.0021	0.0000
ADHD	Calcium	4	-3.2691	0.7941	-0.0085	0.0021	0.3396	0.0022	0.0000
ADHD	Calcium	5	-2.2546	0.7923	-0.0058	0.0020	0.3392	0.0021	0.0044
ADHD	Carbohydrate	0	-1.3577	0.1829	-0.0155	0.0021	0.4091	0.0019	0.0000
ADHD	Carbohydrate	1	-1.2531	0.1827	-0.0143	0.0021	0.4099	0.0019	0.0000
ADHD	Carbohydrate	2	-1.0787	0.1832	-0.0123	0.0021	0.4098	0.0019	0.0000
ADHD	Carbohydrate	3	-1.2934	0.1819	-0.0148	0.0021	0.4095	0.0019	0.0000
ADHD	Carbohydrate	4	-1.3186	0.1809	-0.0150	0.0021	0.4096	0.0019	0.0000
ADHD	Carbohydrate	5	-1.1099	0.1804	-0.0127	0.0021	0.4090	0.0019	0.0000
ADHD	Carotene	0	5.4340	5.6651	0.0019	0.0020	0.2547	0.0026	0.3375
ADHD	Carotene	1	-0.5861	5.6006	-0.0002	0.0020	0.2604	0.0026	0.9167
ADHD	Carotene	2	8.2011	5.6117	0.0029	0.0020	0.2604	0.0026	0.1439
ADHD	Carotene	3	-1.5961	5.5516	-0.0006	0.0019	0.2573	0.0026	0.7737
ADHD	Carotene	4	3.2866	5.6011	0.0011	0.0020	0.2604	0.0026	0.5574
ADHD	Carotene	5	9.3027	5.5614	0.0033	0.0019	0.2574	0.0026	0.0944
ADHD	Fibre	0	-0.0881	0.0151	-0.0125	0.0021	0.4361	0.0016	0.0000
ADHD	Fibre	1	-0.1018	0.0149	-0.0145	0.0021	0.4390	0.0016	0.0000
ADHD	Fibre	2	-0.0653	0.0149	-0.0093	0.0021	0.4388	0.0016	0.0000
ADHD	Fibre	3	-0.1040	0.0147	-0.0148	0.0021	0.4362	0.0016	0.0000
ADHD	Fibre	4	-0.0896	0.0148	-0.0128	0.0021	0.4393	0.0016	0.0000
ADHD	Fibre	5	-0.0584	0.0146	-0.0083	0.0021	0.4364	0.0016	0.0001
ADHD	Fat	0	-0.2871	0.0647	-0.0090	0.0020	0.3347	0.0020	0.0000
ADHD	Fat	1	-0.1984	0.0642	-0.0062	0.0020	0.3411	0.0020	0.0020
ADHD	Fat	2	-0.1454	0.0644	-0.0046	0.0020	0.3411	0.0020	0.0239
ADHD	Fat	3	-0.1968	0.0640	-0.0062	0.0020	0.3411	0.0020	0.0021
ADHD	Fat	4	-0.2299	0.0642	-0.0072	0.0020	0.3410	0.0020	0.0003
ADHD	Fat	5	-0.1696	0.0642	-0.0053	0.0020	0.3411	0.0020	0.0083

ADHD	Folate	0	-1.4782	0.2503	-0.0125	0.0021	0.4061	0.0017	0.0000
ADHD	Folate	1	-1.5937	0.2490	-0.0134	0.0021	0.4069	0.0017	0.0000
ADHD	Folate	2	-1.0775	0.2493	-0.0091	0.0021	0.4068	0.0017	0.0000
ADHD	Folate	3	-1.6283	0.2470	-0.0137	0.0021	0.4050	0.0017	0.0000
ADHD	Folate	4	-1.3636	0.2489	-0.0115	0.0021	0.4069	0.0017	0.0000
ADHD	Folate	5	-0.9937	0.2471	-0.0084	0.0021	0.4050	0.0017	0.0001
ADHD	Food weight	0	4.8273	1.7960	0.0060	0.0022	0.5365	0.0015	0.0072
ADHD	Food weight	1	4.2241	1.7946	0.0052	0.0022	0.5359	0.0015	0.0186
ADHD	Food weight	2	7.3301	1.7977	0.0091	0.0022	0.5359	0.0015	0.0000
ADHD	Food weight	3	3.6362	1.7710	0.0045	0.0022	0.5341	0.0015	0.0401
ADHD	Food weight	4	5.9037	1.7771	0.0073	0.0022	0.5357	0.0015	0.0009
ADHD	Food weight	5	6.8964	1.7566	0.0085	0.0022	0.5339	0.0015	0.0001
ADHD	Iron	0	-0.0938	0.0096	-0.0201	0.0021	0.3692	0.0017	0.0000
ADHD	Iron	1	-0.0927	0.0096	-0.0198	0.0021	0.3691	0.0017	0.0000
ADHD	Iron	2	-0.0539	0.0096	-0.0115	0.0021	0.3691	0.0017	0.0000
ADHD	Iron	3	-0.0926	0.0096	-0.0198	0.0021	0.3684	0.0017	0.0000
ADHD	Iron	4	-0.0725	0.0095	-0.0155	0.0020	0.3691	0.0017	0.0000
ADHD	Iron	5	-0.0458	0.0095	-0.0098	0.0020	0.3685	0.0017	0.0000
ADHD	Protein	0	0.0177	0.0539	0.0007	0.0020	0.2924	0.0023	0.7420
ADHD	Protein	1	0.0520	0.0537	0.0019	0.0020	0.2917	0.0023	0.3328
ADHD	Protein	2	0.0824	0.0539	0.0031	0.0020	0.2918	0.0023	0.1261
ADHD	Protein	3	0.0537	0.0536	0.0020	0.0020	0.2918	0.0023	0.3167
ADHD	Protein	4	0.0695	0.0538	0.0026	0.0020	0.2916	0.0023	0.1963
ADHD	Protein	5	0.0900	0.0537	0.0033	0.0020	0.2919	0.0023	0.0940
ADHD	Vitamin B12	0	-0.0324	0.0089	-0.0068	0.0019	0.1574	0.0030	0.0003
ADHD	Vitamin B12	1	-0.0279	0.0089	-0.0059	0.0019	0.1566	0.0030	0.0017
ADHD	Vitamin B12	2	-0.0131	0.0089	-0.0028	0.0019	0.1570	0.0030	0.1410
ADHD	Vitamin B12	3	-0.0272	0.0089	-0.0057	0.0019	0.1568	0.0030	0.0023
ADHD	Vitamin B12	4	-0.0210	0.0089	-0.0044	0.0019	0.1567	0.0030	0.0186
ADHD	Vitamin B12	5	-0.0099	0.0089	-0.0021	0.0019	0.1572	0.0030	0.2659
ADHD	Vitamin B6	0	-0.0012	0.0016	-0.0016	0.0020	0.3391	0.0018	0.4378
ADHD	Vitamin B6	1	-0.0014	0.0016	-0.0017	0.0020	0.3402	0.0018	0.3920
ADHD	Vitamin B6	2	-0.0017	0.0016	-0.0022	0.0020	0.3402	0.0018	0.2818
ADHD	Vitamin B6	3	-0.0015	0.0016	-0.0020	0.0020	0.3396	0.0018	0.3321
ADHD	Vitamin B6	4	-0.0001	0.0016	-0.0002	0.0020	0.3402	0.0018	0.9399
ADHD	Vitamin B6	5	-0.0012	0.0016	-0.0015	0.0020	0.3397	0.0018	0.4601
ADHD	Vitamin C	0	-1.2589	0.2340	-0.0114	0.0021	0.3963	0.0024	0.0000
ADHD	Vitamin C	1	-1.3886	0.2329	-0.0126	0.0021	0.3996	0.0024	0.0000

ADHD	Vitamin C	2	-0.5007	0.2320	-0.0045	0.0021	0.3995	0.0024	0.0309
ADHD	Vitamin C	3	-1.4200	0.2310	-0.0128	0.0021	0.3971	0.0024	0.0000
ADHD	Vitamin C	4	-1.0631	0.2325	-0.0096	0.0021	0.3996	0.0023	0.0000
ADHD	Vitamin C	5	-0.3782	0.2298	-0.0034	0.0021	0.3972	0.0024	0.0998
ADHD	Vitamin D	0	-0.0237	0.0060	-0.0074	0.0019	0.1266	0.0033	0.0001
ADHD	Vitamin D	1	-0.0232	0.0060	-0.0072	0.0019	0.1267	0.0033	0.0001
ADHD	Vitamin D	2	-0.0150	0.0060	-0.0047	0.0019	0.1270	0.0033	0.0120
ADHD	Vitamin D	3	-0.0230	0.0060	-0.0071	0.0019	0.1269	0.0033	0.0001
ADHD	Vitamin D	4	-0.0194	0.0060	-0.0060	0.0019	0.1268	0.0033	0.0011
ADHD	Vitamin D	5	-0.0133	0.0060	-0.0041	0.0019	0.1271	0.0033	0.0265
ADHD	Vitamin E	0	-0.0850	0.0097	-0.0178	0.0020	0.2960	0.0021	0.0000
ADHD	Vitamin E	1	-0.0887	0.0097	-0.0186	0.0020	0.2955	0.0021	0.0000
ADHD	Vitamin E	2	-0.0534	0.0096	-0.0112	0.0020	0.2956	0.0021	0.0000
ADHD	Vitamin E	3	-0.0886	0.0096	-0.0186	0.0020	0.2947	0.0021	0.0000
ADHD	Vitamin E	4	-0.0807	0.0096	-0.0169	0.0020	0.2956	0.0021	0.0000
ADHD	Vitamin E	5	-0.0495	0.0096	-0.0104	0.0020	0.2951	0.0021	0.0000
Alcohol dependence	Alcohol	0	0.3941	0.0510	0.0173	0.0022	0.4792	0.0020	0.0000
Alcohol dependence	Alcohol	1	0.4020	0.0503	0.0177	0.0022	0.4964	0.0019	0.0000
Alcohol dependence	Alcohol	2	0.4207	0.0503	0.0185	0.0022	0.4966	0.0019	0.0000
Alcohol dependence	Alcohol	3	0.4020	0.0503	0.0177	0.0022	0.4973	0.0019	0.0000
Alcohol dependence	Alcohol	4	0.2522	0.0414	0.0111	0.0018	0.4962	0.0020	0.0000
Alcohol dependence	Alcohol	5	0.2273	0.0414	0.0100	0.0018	0.4971	0.0020	0.0000
Alcohol dependence	Calcium	0	-3.4164	0.8415	-0.0088	0.0022	0.3396	0.0022	0.0000
Alcohol dependence	Calcium	1	-3.4128	0.8410	-0.0088	0.0022	0.3397	0.0022	0.0000
Alcohol dependence	Calcium	2	-3.0280	0.8404	-0.0078	0.0022	0.3395	0.0022	0.0003
Alcohol dependence	Calcium	3	-3.4001	0.8379	-0.0088	0.0022	0.3393	0.0021	0.0000
Alcohol dependence	Calcium	4	-2.7235	0.8373	-0.0070	0.0022	0.3396	0.0022	0.0011
Alcohol dependence	Calcium	5	-2.2996	0.8333	-0.0059	0.0022	0.3392	0.0021	0.0058
Alcohol dependence	Carbohydrate	0	-1.1803	0.1931	-0.0135	0.0022	0.4091	0.0019	0.0000
Alcohol dependence	Carbohydrate	1	-1.1688	0.1928	-0.0133	0.0022	0.4098	0.0019	0.0000
Alcohol dependence	Carbohydrate	2	-1.1076	0.1928	-0.0126	0.0022	0.4097	0.0019	0.0000
Alcohol dependence	Carbohydrate	3	-1.1754	0.1919	-0.0134	0.0022	0.4094	0.0019	0.0000
Alcohol dependence	Carbohydrate	4	-0.9771	0.1908	-0.0111	0.0022	0.4095	0.0019	0.0000
Alcohol dependence	Carbohydrate	5	-0.8930	0.1897	-0.0102	0.0022	0.4090	0.0019	0.0000
Alcohol dependence	Carotene	0	-0.4307	5.9797	-0.0002	0.0021	0.2547	0.0026	0.9426
Alcohol dependence	Carotene	1	-1.4101	5.9097	-0.0005	0.0021	0.2604	0.0026	0.8114
Alcohol dependence	Carotene	2	1.6006	5.9041	0.0006	0.0021	0.2604	0.0026	0.7863
Alcohol dependence	Carotene	3	-1.3648	5.8570	-0.0005	0.0020	0.2573	0.0026	0.8157

Alcohol dependence	Carotene	4	1.0478	5.9055	0.0004	0.0021	0.2604	0.0026	0.8592
Alcohol dependence	Carotene	5	3.5144	5.8487	0.0012	0.0020	0.2574	0.0026	0.5479
Alcohol dependence	Fibre	0	-0.0490	0.0159	-0.0070	0.0023	0.4361	0.0016	0.0021
Alcohol dependence	Fibre	1	-0.0514	0.0157	-0.0073	0.0022	0.4391	0.0016	0.0011
Alcohol dependence	Fibre	2	-0.0383	0.0157	-0.0055	0.0022	0.4388	0.0016	0.0144
Alcohol dependence	Fibre	3	-0.0510	0.0155	-0.0073	0.0022	0.4362	0.0016	0.0010
Alcohol dependence	Fibre	4	-0.0325	0.0156	-0.0046	0.0022	0.4393	0.0016	0.0374
Alcohol dependence	Fibre	5	-0.0198	0.0154	-0.0028	0.0022	0.4364	0.0016	0.1984
Alcohol dependence	Fat	0	-0.1259	0.0683	-0.0039	0.0021	0.3347	0.0020	0.0654
Alcohol dependence	Fat	1	-0.1130	0.0677	-0.0035	0.0021	0.3411	0.0020	0.0953
Alcohol dependence	Fat	2	-0.0957	0.0677	-0.0030	0.0021	0.3411	0.0020	0.1579
Alcohol dependence	Fat	3	-0.1099	0.0676	-0.0034	0.0021	0.3411	0.0020	0.1037
Alcohol dependence	Fat	4	-0.1172	0.0677	-0.0037	0.0021	0.3410	0.0020	0.0834
Alcohol dependence	Fat	5	-0.0933	0.0675	-0.0029	0.0021	0.3411	0.0020	0.1673
Alcohol dependence	Folate	0	-0.6424	0.2643	-0.0054	0.0022	0.4061	0.0017	0.0151
Alcohol dependence	Folate	1	-0.6642	0.2628	-0.0056	0.0022	0.4069	0.0017	0.0115
Alcohol dependence	Folate	2	-0.4724	0.2623	-0.0040	0.0022	0.4068	0.0017	0.0717
Alcohol dependence	Folate	3	-0.6585	0.2606	-0.0056	0.0022	0.4051	0.0017	0.0115
Alcohol dependence	Folate	4	-0.5178	0.2625	-0.0044	0.0022	0.4069	0.0017	0.0485
Alcohol dependence	Folate	5	-0.3586	0.2599	-0.0030	0.0022	0.4050	0.0017	0.1677
Alcohol dependence	Food weight	0	5.8972	1.8960	0.0073	0.0023	0.5365	0.0015	0.0019
Alcohol dependence	Food weight	1	5.8217	1.8940	0.0072	0.0023	0.5359	0.0015	0.0021
Alcohol dependence	Food weight	2	6.9481	1.8918	0.0086	0.0023	0.5359	0.0015	0.0002
Alcohol dependence	Food weight	3	5.7192	1.8689	0.0071	0.0023	0.5341	0.0015	0.0022
Alcohol dependence	Food weight	4	4.5911	1.8740	0.0057	0.0023	0.5357	0.0015	0.0143
Alcohol dependence	Food weight	5	5.0782	1.8478	0.0063	0.0023	0.5339	0.0015	0.0060
Alcohol dependence	Iron	0	-0.0345	0.0102	-0.0074	0.0022	0.3692	0.0017	0.0007
Alcohol dependence	Iron	1	-0.0344	0.0102	-0.0074	0.0022	0.3691	0.0017	0.0007
Alcohol dependence	Iron	2	-0.0205	0.0101	-0.0044	0.0022	0.3691	0.0017	0.0420
Alcohol dependence	Iron	3	-0.0337	0.0101	-0.0072	0.0022	0.3684	0.0017	0.0009
Alcohol dependence	Iron	4	-0.0322	0.0101	-0.0069	0.0022	0.3691	0.0017	0.0014
Alcohol dependence	Iron	5	-0.0218	0.0100	-0.0047	0.0021	0.3685	0.0017	0.0285
Alcohol dependence	Protein	0	-0.0906	0.0568	-0.0034	0.0021	0.2924	0.0023	0.1109
Alcohol dependence	Protein	1	-0.0863	0.0567	-0.0032	0.0021	0.2917	0.0023	0.1278
Alcohol dependence	Protein	2	-0.0718	0.0567	-0.0027	0.0021	0.2918	0.0023	0.2052
Alcohol dependence	Protein	3	-0.0832	0.0566	-0.0031	0.0021	0.2918	0.0023	0.1413
Alcohol dependence	Protein	4	-0.0697	0.0567	-0.0026	0.0021	0.2917	0.0023	0.2189
Alcohol dependence	Protein	5	-0.0566	0.0565	-0.0021	0.0021	0.2919	0.0023	0.3168

Alcohol dependence	Vitamin B12	0	-0.0068	0.0094	-0.0014	0.0020	0.1574	0.0030	0.4685
Alcohol dependence	Vitamin B12	1	-0.0064	0.0094	-0.0013	0.0020	0.1566	0.0030	0.4996
Alcohol dependence	Vitamin B12	2	-0.0008	0.0094	-0.0002	0.0020	0.1570	0.0030	0.9319
Alcohol dependence	Vitamin B12	3	-0.0059	0.0094	-0.0012	0.0020	0.1568	0.0030	0.5320
Alcohol dependence	Vitamin B12	4	-0.0051	0.0094	-0.0011	0.0020	0.1567	0.0030	0.5850
Alcohol dependence	Vitamin B12	5	-0.0007	0.0094	-0.0001	0.0020	0.1572	0.0030	0.9421
Alcohol dependence	Vitamin B6	0	-0.0017	0.0017	-0.0022	0.0022	0.3391	0.0018	0.3150
Alcohol dependence	Vitamin B6	1	-0.0018	0.0017	-0.0023	0.0022	0.3402	0.0018	0.2947
Alcohol dependence	Vitamin B6	2	-0.0016	0.0017	-0.0021	0.0021	0.3402	0.0018	0.3346
Alcohol dependence	Vitamin B6	3	-0.0018	0.0017	-0.0022	0.0021	0.3396	0.0018	0.2953
Alcohol dependence	Vitamin B6	4	-0.0009	0.0017	-0.0012	0.0021	0.3402	0.0018	0.5869
Alcohol dependence	Vitamin B6	5	-0.0010	0.0017	-0.0013	0.0021	0.3397	0.0018	0.5440
Alcohol dependence	Vitamin C	0	-0.5508	0.2471	-0.0050	0.0022	0.3963	0.0024	0.0258
Alcohol dependence	Vitamin C	1	-0.5747	0.2459	-0.0052	0.0022	0.3996	0.0024	0.0194
Alcohol dependence	Vitamin C	2	-0.2554	0.2441	-0.0023	0.0022	0.3995	0.0024	0.2954
Alcohol dependence	Vitamin C	3	-0.5716	0.2438	-0.0052	0.0022	0.3971	0.0024	0.0190
Alcohol dependence	Vitamin C	4	-0.4252	0.2452	-0.0038	0.0022	0.3996	0.0023	0.0829
Alcohol dependence	Vitamin C	5	-0.1575	0.2417	-0.0014	0.0022	0.3972	0.0024	0.5146
Alcohol dependence	Vitamin D	0	-0.0024	0.0063	-0.0007	0.0020	0.1266	0.0033	0.7028
Alcohol dependence	Vitamin D	1	-0.0024	0.0063	-0.0007	0.0020	0.1267	0.0033	0.7026
Alcohol dependence	Vitamin D	2	0.0006	0.0063	0.0002	0.0020	0.1270	0.0033	0.9237
Alcohol dependence	Vitamin D	3	-0.0023	0.0063	-0.0007	0.0020	0.1269	0.0033	0.7202
Alcohol dependence	Vitamin D	4	-0.0012	0.0063	-0.0004	0.0020	0.1268	0.0033	0.8483
Alcohol dependence	Vitamin D	5	0.0012	0.0063	0.0004	0.0020	0.1271	0.0033	0.8467
Alcohol dependence	Vitamin E	0	-0.0264	0.0102	-0.0055	0.0021	0.2960	0.0021	0.0097
Alcohol dependence	Vitamin E	1	-0.0270	0.0102	-0.0057	0.0021	0.2955	0.0021	0.0081
Alcohol dependence	Vitamin E	2	-0.0147	0.0101	-0.0031	0.0021	0.2956	0.0021	0.1478
Alcohol dependence	Vitamin E	3	-0.0262	0.0101	-0.0055	0.0021	0.2947	0.0021	0.0098
Alcohol dependence	Vitamin E	4	-0.0195	0.0102	-0.0041	0.0021	0.2956	0.0021	0.0554
Alcohol dependence	Vitamin E	5	-0.0075	0.0101	-0.0016	0.0021	0.2951	0.0021	0.4567
Anorexia Nervosa	Alcohol	0	-0.0346	0.0484	-0.0015	0.0021	0.4792	0.0020	0.4751
Anorexia Nervosa	Alcohol	1	-0.0247	0.0478	-0.0011	0.0021	0.4965	0.0019	0.6051
Anorexia Nervosa	Alcohol	2	-0.0398	0.0478	-0.0018	0.0021	0.4966	0.0019	0.4043
Anorexia Nervosa	Alcohol	3	-0.0227	0.0478	-0.0010	0.0021	0.4973	0.0019	0.6344
Anorexia Nervosa	Alcohol	4	-0.0887	0.0394	-0.0039	0.0017	0.4962	0.0020	0.0243
Anorexia Nervosa	Alcohol	5	-0.0708	0.0393	-0.0031	0.0017	0.4971	0.0020	0.0714
Anorexia Nervosa	Calcium	0	0.9764	0.7996	0.0025	0.0021	0.3396	0.0022	0.2221
Anorexia Nervosa	Calcium	1	0.9602	0.7992	0.0025	0.0021	0.3397	0.0022	0.2296

Anorexia Nervosa	Calcium	2	0.7296	0.7985	0.0019	0.0021	0.3395	0.0022	0.3608
Anorexia Nervosa	Calcium	3	0.7896	0.7962	0.0020	0.0021	0.3393	0.0021	0.3214
Anorexia Nervosa	Calcium	4	1.0859	0.7954	0.0028	0.0021	0.3397	0.0022	0.1722
Anorexia Nervosa	Calcium	5	0.6257	0.7915	0.0016	0.0020	0.3392	0.0021	0.4292
Anorexia Nervosa	Carbohydrate	0	0.1329	0.1835	0.0015	0.0021	0.4091	0.0019	0.4688
Anorexia Nervosa	Carbohydrate	1	0.1351	0.1832	0.0015	0.0021	0.4099	0.0019	0.4609
Anorexia Nervosa	Carbohydrate	2	0.0904	0.1831	0.0010	0.0021	0.4098	0.0019	0.6216
Anorexia Nervosa	Carbohydrate	3	0.1005	0.1823	0.0011	0.0021	0.4095	0.0019	0.5816
Anorexia Nervosa	Carbohydrate	4	0.1813	0.1812	0.0021	0.0021	0.4096	0.0019	0.3172
Anorexia Nervosa	Carbohydrate	5	0.0751	0.1802	0.0009	0.0021	0.4091	0.0019	0.6767
Anorexia Nervosa	Carotene	0	11.2952	5.6825	0.0039	0.0020	0.2547	0.0026	0.0468
Anorexia Nervosa	Carotene	1	10.2954	5.6160	0.0036	0.0020	0.2604	0.0026	0.0668
Anorexia Nervosa	Carotene	2	8.1020	5.6101	0.0028	0.0020	0.2604	0.0026	0.1487
Anorexia Nervosa	Carotene	3	8.6068	5.5661	0.0030	0.0019	0.2573	0.0026	0.1220
Anorexia Nervosa	Carotene	4	10.4414	5.6103	0.0037	0.0020	0.2604	0.0026	0.0627
Anorexia Nervosa	Carotene	5	6.6980	5.5562	0.0023	0.0019	0.2574	0.0026	0.2280
Anorexia Nervosa	Fibre	0	0.0568	0.0151	0.0081	0.0022	0.4361	0.0016	0.0002
Anorexia Nervosa	Fibre	1	0.0543	0.0149	0.0077	0.0021	0.4391	0.0016	0.0003
Anorexia Nervosa	Fibre	2	0.0453	0.0149	0.0065	0.0021	0.4388	0.0016	0.0023
Anorexia Nervosa	Fibre	3	0.0498	0.0148	0.0071	0.0021	0.4362	0.0016	0.0008
Anorexia Nervosa	Fibre	4	0.0576	0.0148	0.0082	0.0021	0.4393	0.0016	0.0001
Anorexia Nervosa	Fibre	5	0.0434	0.0146	0.0062	0.0021	0.4364	0.0016	0.0030
Anorexia Nervosa	Fat	0	-0.0804	0.0649	-0.0025	0.0020	0.3347	0.0020	0.2156
Anorexia Nervosa	Fat	1	-0.0708	0.0644	-0.0022	0.0020	0.3411	0.0020	0.2714
Anorexia Nervosa	Fat	2	-0.0869	0.0644	-0.0027	0.0020	0.3411	0.0020	0.1771
Anorexia Nervosa	Fat	3	-0.0750	0.0642	-0.0024	0.0020	0.3411	0.0020	0.2425
Anorexia Nervosa	Fat	4	-0.0725	0.0643	-0.0023	0.0020	0.3410	0.0020	0.2597
Anorexia Nervosa	Fat	5	-0.0946	0.0641	-0.0030	0.0020	0.3411	0.0020	0.1401
Anorexia Nervosa	Folate	0	0.9261	0.2510	0.0078	0.0021	0.4061	0.0017	0.0002
Anorexia Nervosa	Folate	1	0.9002	0.2497	0.0076	0.0021	0.4069	0.0017	0.0003
Anorexia Nervosa	Folate	2	0.7813	0.2492	0.0066	0.0021	0.4068	0.0017	0.0017
Anorexia Nervosa	Folate	3	0.8318	0.2476	0.0070	0.0021	0.4051	0.0017	0.0008
Anorexia Nervosa	Folate	4	0.9087	0.2493	0.0077	0.0021	0.4069	0.0017	0.0003
Anorexia Nervosa	Folate	5	0.7302	0.2468	0.0062	0.0021	0.4050	0.0017	0.0031
Anorexia Nervosa	Food weight	0	1.7748	1.8010	0.0022	0.0022	0.5365	0.0015	0.3244
Anorexia Nervosa	Food weight	1	1.7365	1.7990	0.0021	0.0022	0.5359	0.0015	0.3344
Anorexia Nervosa	Food weight	2	0.9942	1.7968	0.0012	0.0022	0.5359	0.0015	0.5801
Anorexia Nervosa	Food weight	3	1.2480	1.7752	0.0015	0.0022	0.5341	0.0015	0.4820

Anorexia Nervosa	Food weight	4	1.0521	1.7795	0.0013	0.0022	0.5357	0.0015	0.5543
Anorexia Nervosa	Food weight	5	0.0959	1.7545	0.0001	0.0022	0.5339	0.0015	0.9564
Anorexia Nervosa	Iron	0	0.0347	0.0097	0.0074	0.0021	0.3692	0.0017	0.0003
Anorexia Nervosa	Iron	1	0.0346	0.0097	0.0074	0.0021	0.3691	0.0017	0.0003
Anorexia Nervosa	Iron	2	0.0248	0.0096	0.0053	0.0021	0.3691	0.0017	0.0097
Anorexia Nervosa	Iron	3	0.0324	0.0096	0.0069	0.0021	0.3684	0.0017	0.0007
Anorexia Nervosa	Iron	4	0.0324	0.0096	0.0069	0.0020	0.3691	0.0017	0.0007
Anorexia Nervosa	Iron	5	0.0226	0.0095	0.0048	0.0020	0.3685	0.0017	0.0167
Anorexia Nervosa	Protein	0	-0.0473	0.0540	-0.0018	0.0020	0.2924	0.0023	0.3808
Anorexia Nervosa	Protein	1	-0.0457	0.0539	-0.0017	0.0020	0.2917	0.0023	0.3967
Anorexia Nervosa	Protein	2	-0.0476	0.0539	-0.0018	0.0020	0.2918	0.0023	0.3768
Anorexia Nervosa	Protein	3	-0.0547	0.0537	-0.0020	0.0020	0.2918	0.0023	0.3087
Anorexia Nervosa	Protein	4	-0.0432	0.0538	-0.0016	0.0020	0.2916	0.0023	0.4225
Anorexia Nervosa	Protein	5	-0.0538	0.0537	-0.0020	0.0020	0.2919	0.0023	0.3163
Anorexia Nervosa	Vitamin B12	0	0.0093	0.0090	0.0020	0.0019	0.1574	0.0030	0.2995
Anorexia Nervosa	Vitamin B12	1	0.0094	0.0089	0.0020	0.0019	0.1566	0.0030	0.2938
Anorexia Nervosa	Vitamin B12	2	0.0061	0.0089	0.0013	0.0019	0.1570	0.0030	0.4974
Anorexia Nervosa	Vitamin B12	3	0.0084	0.0089	0.0018	0.0019	0.1568	0.0030	0.3457
Anorexia Nervosa	Vitamin B12	4	0.0091	0.0089	0.0019	0.0019	0.1567	0.0030	0.3085
Anorexia Nervosa	Vitamin B12	5	0.0055	0.0089	0.0012	0.0019	0.1572	0.0030	0.5377
Anorexia Nervosa	Vitamin B6	0	-0.0007	0.0016	-0.0009	0.0021	0.3391	0.0018	0.6498
Anorexia Nervosa	Vitamin B6	1	-0.0009	0.0016	-0.0011	0.0020	0.3402	0.0018	0.5891
Anorexia Nervosa	Vitamin B6	2	-0.0004	0.0016	-0.0005	0.0020	0.3402	0.0018	0.7915
Anorexia Nervosa	Vitamin B6	3	-0.0012	0.0016	-0.0016	0.0020	0.3396	0.0018	0.4344
Anorexia Nervosa	Vitamin B6	4	-0.0007	0.0016	-0.0010	0.0020	0.3402	0.0018	0.6393
Anorexia Nervosa	Vitamin B6	5	-0.0007	0.0016	-0.0009	0.0020	0.3397	0.0018	0.6725
Anorexia Nervosa	Vitamin C	0	0.9236	0.2347	0.0083	0.0021	0.3963	0.0024	0.0001
Anorexia Nervosa	Vitamin C	1	0.8972	0.2336	0.0081	0.0021	0.3996	0.0024	0.0001
Anorexia Nervosa	Vitamin C	2	0.6781	0.2319	0.0061	0.0021	0.3995	0.0024	0.0035
Anorexia Nervosa	Vitamin C	3	0.8377	0.2316	0.0076	0.0021	0.3971	0.0024	0.0003
Anorexia Nervosa	Vitamin C	4	0.9015	0.2329	0.0081	0.0021	0.3996	0.0023	0.0001
Anorexia Nervosa	Vitamin C	5	0.6402	0.2295	0.0058	0.0021	0.3972	0.0024	0.0053
Anorexia Nervosa	Vitamin D	0	0.0109	0.0060	0.0034	0.0019	0.1266	0.0033	0.0684
Anorexia Nervosa	Vitamin D	1	0.0108	0.0060	0.0033	0.0019	0.1268	0.0033	0.0715
Anorexia Nervosa	Vitamin D	2	0.0089	0.0060	0.0027	0.0019	0.1270	0.0033	0.1387
Anorexia Nervosa	Vitamin D	3	0.0102	0.0060	0.0032	0.0019	0.1269	0.0033	0.0889
Anorexia Nervosa	Vitamin D	4	0.0107	0.0060	0.0033	0.0019	0.1268	0.0033	0.0722
Anorexia Nervosa	Vitamin D	5	0.0085	0.0060	0.0026	0.0019	0.1271	0.0033	0.1537

Anorexia Nervosa	Vitamin E	0	0.0323	0.0097	0.0068	0.0020	0.2960	0.0021	0.0009
Anorexia Nervosa	Vitamin E	1	0.0318	0.0097	0.0067	0.0020	0.2955	0.0021	0.0010
Anorexia Nervosa	Vitamin E	2	0.0225	0.0096	0.0047	0.0020	0.2956	0.0021	0.0194
Anorexia Nervosa	Vitamin E	3	0.0294	0.0096	0.0062	0.0020	0.2947	0.0021	0.0023
Anorexia Nervosa	Vitamin E	4	0.0323	0.0097	0.0068	0.0020	0.2956	0.0021	0.0008
Anorexia Nervosa	Vitamin E	5	0.0209	0.0095	0.0044	0.0020	0.2951	0.0021	0.0286
Autism spectrum disorder	Alcohol	0	-0.0433	0.0484	-0.0019	0.0021	0.4792	0.0020	0.3712
Autism spectrum disorder	Alcohol	1	-0.0507	0.0478	-0.0022	0.0021	0.4965	0.0019	0.2890
Autism spectrum disorder	Alcohol	2	-0.0739	0.0478	-0.0033	0.0021	0.4966	0.0019	0.1221
Autism spectrum disorder	Alcohol	3	-0.0512	0.0478	-0.0023	0.0021	0.4973	0.0019	0.2837
Autism spectrum disorder	Alcohol	4	0.0120	0.0394	0.0005	0.0017	0.4962	0.0020	0.7601
Autism spectrum disorder	Alcohol	5	0.0244	0.0393	0.0011	0.0017	0.4971	0.0020	0.5346
Autism spectrum disorder	Calcium	0	1.0172	0.7994	0.0026	0.0021	0.3396	0.0022	0.2032
Autism spectrum disorder	Calcium	1	1.1176	0.7989	0.0029	0.0021	0.3397	0.0022	0.1619
Autism spectrum disorder	Calcium	2	0.9585	0.7986	0.0025	0.0021	0.3395	0.0022	0.2301
Autism spectrum disorder	Calcium	3	1.1076	0.7960	0.0029	0.0021	0.3393	0.0021	0.1641
Autism spectrum disorder	Calcium	4	0.9397	0.7952	0.0024	0.0021	0.3396	0.0022	0.2373
Autism spectrum disorder	Calcium	5	0.6405	0.7916	0.0017	0.0020	0.3392	0.0021	0.4184
Autism spectrum disorder	Carbohydrate	0	0.1546	0.1834	0.0018	0.0021	0.4091	0.0019	0.3991
Autism spectrum disorder	Carbohydrate	1	0.2060	0.1831	0.0023	0.0021	0.4099	0.0019	0.2607
Autism spectrum disorder	Carbohydrate	2	0.1656	0.1831	0.0019	0.0021	0.4098	0.0019	0.3660
Autism spectrum disorder	Carbohydrate	3	0.2099	0.1823	0.0024	0.0021	0.4095	0.0019	0.2496
Autism spectrum disorder	Carbohydrate	4	0.1313	0.1812	0.0015	0.0021	0.4096	0.0019	0.4686
Autism spectrum disorder	Carbohydrate	5	0.0534	0.1802	0.0006	0.0021	0.4091	0.0019	0.7671
Autism spectrum disorder	Carotene	0	6.9054	5.6811	0.0024	0.0020	0.2547	0.0026	0.2242
Autism spectrum disorder	Carotene	1	6.6029	5.6151	0.0023	0.0020	0.2604	0.0026	0.2396
Autism spectrum disorder	Carotene	2	4.1997	5.6114	0.0015	0.0020	0.2604	0.0026	0.4542
Autism spectrum disorder	Carotene	3	6.8484	5.5651	0.0024	0.0019	0.2573	0.0026	0.2185
Autism spectrum disorder	Carotene	4	7.2363	5.6097	0.0025	0.0020	0.2604	0.0026	0.1971
Autism spectrum disorder	Carotene	5	4.6782	5.5578	0.0016	0.0019	0.2574	0.0026	0.3999
Autism spectrum disorder	Fibre	0	0.0239	0.0151	0.0034	0.0022	0.4361	0.0016	0.1134
Autism spectrum disorder	Fibre	1	0.0236	0.0149	0.0034	0.0021	0.4391	0.0016	0.1137
Autism spectrum disorder	Fibre	2	0.0149	0.0149	0.0021	0.0021	0.4388	0.0016	0.3162
Autism spectrum disorder	Fibre	3	0.0240	0.0148	0.0034	0.0021	0.4362	0.0016	0.1040
Autism spectrum disorder	Fibre	4	0.0218	0.0148	0.0031	0.0021	0.4393	0.0016	0.1404
Autism spectrum disorder	Fibre	5	0.0107	0.0146	0.0015	0.0021	0.4364	0.0016	0.4651
Autism spectrum disorder	Fat	0	0.1577	0.0649	0.0049	0.0020	0.3347	0.0020	0.0151
Autism spectrum disorder	Fat	1	0.1782	0.0644	0.0056	0.0020	0.3411	0.0020	0.0056

Autism spectrum disorder	Fat	2	0.1563	0.0644	0.0049	0.0020	0.3411	0.0020	0.0151
Autism spectrum disorder	Fat	3	0.1701	0.0642	0.0053	0.0020	0.3411	0.0020	0.0081
Autism spectrum disorder	Fat	4	0.1643	0.0643	0.0052	0.0020	0.3410	0.0020	0.0106
Autism spectrum disorder	Fat	5	0.1321	0.0642	0.0041	0.0020	0.3411	0.0020	0.0395
Autism spectrum disorder	Folate	0	-0.1006	0.2510	-0.0008	0.0021	0.4061	0.0017	0.6884
Autism spectrum disorder	Folate	1	-0.0885	0.2496	-0.0007	0.0021	0.4069	0.0017	0.7228
Autism spectrum disorder	Folate	2	-0.1874	0.2492	-0.0016	0.0021	0.4068	0.0017	0.4522
Autism spectrum disorder	Folate	3	-0.0846	0.2475	-0.0007	0.0021	0.4051	0.0017	0.7327
Autism spectrum disorder	Folate	4	-0.0642	0.2492	-0.0005	0.0021	0.4069	0.0017	0.7968
Autism spectrum disorder	Folate	5	-0.1797	0.2469	-0.0015	0.0021	0.4050	0.0017	0.4666
Autism spectrum disorder	Food weight	0	-0.1209	1.8001	-0.0001	0.0022	0.5365	0.0015	0.9465
Autism spectrum disorder	Food weight	1	-0.3457	1.7983	-0.0004	0.0022	0.5359	0.0015	0.8476
Autism spectrum disorder	Food weight	2	-1.2028	1.7967	-0.0015	0.0022	0.5359	0.0015	0.5032
Autism spectrum disorder	Food weight	3	-0.0675	1.7745	-0.0001	0.0022	0.5341	0.0015	0.9697
Autism spectrum disorder	Food weight	4	0.2380	1.7789	0.0003	0.0022	0.5357	0.0015	0.8936
Autism spectrum disorder	Food weight	5	-0.1600	1.7546	-0.0002	0.0022	0.5339	0.0015	0.9273
Autism spectrum disorder	Iron	0	0.0105	0.0097	0.0023	0.0021	0.3691	0.0017	0.2761
Autism spectrum disorder	Iron	1	0.0113	0.0097	0.0024	0.0021	0.3691	0.0017	0.2416
Autism spectrum disorder	Iron	2	0.0014	0.0096	0.0003	0.0021	0.3691	0.0017	0.8814
Autism spectrum disorder	Iron	3	0.0110	0.0096	0.0024	0.0021	0.3684	0.0017	0.2514
Autism spectrum disorder	Iron	4	0.0151	0.0096	0.0032	0.0020	0.3691	0.0017	0.1145
Autism spectrum disorder	Iron	5	0.0056	0.0095	0.0012	0.0020	0.3685	0.0017	0.5542
Autism spectrum disorder	Protein	0	0.0057	0.0540	0.0002	0.0020	0.2924	0.0023	0.9160
Autism spectrum disorder	Protein	1	0.0191	0.0539	0.0007	0.0020	0.2917	0.0023	0.7235
Autism spectrum disorder	Protein	2	0.0275	0.0539	0.0010	0.0020	0.2918	0.0023	0.6096
Autism spectrum disorder	Protein	3	0.0154	0.0537	0.0006	0.0020	0.2918	0.0023	0.7741
Autism spectrum disorder	Protein	4	0.0191	0.0538	0.0007	0.0020	0.2917	0.0023	0.7229
Autism spectrum disorder	Protein	5	0.0213	0.0537	0.0008	0.0020	0.2919	0.0023	0.6912
Autism spectrum disorder	Vitamin B12	0	-0.0195	0.0090	-0.0041	0.0019	0.1574	0.0030	0.0292
Autism spectrum disorder	Vitamin B12	1	-0.0175	0.0089	-0.0037	0.0019	0.1566	0.0030	0.0505
Autism spectrum disorder	Vitamin B12	2	-0.0197	0.0089	-0.0042	0.0019	0.1570	0.0030	0.0276
Autism spectrum disorder	Vitamin B12	3	-0.0178	0.0089	-0.0038	0.0019	0.1568	0.0030	0.0468
Autism spectrum disorder	Vitamin B12	4	-0.0156	0.0089	-0.0033	0.0019	0.1567	0.0030	0.0812
Autism spectrum disorder	Vitamin B12	5	-0.0181	0.0089	-0.0038	0.0019	0.1572	0.0030	0.0427
Autism spectrum disorder	Vitamin B6	0	-0.0057	0.0016	-0.0072	0.0021	0.3390	0.0018	0.0004
Autism spectrum disorder	Vitamin B6	1	-0.0054	0.0016	-0.0068	0.0020	0.3402	0.0018	0.0008
Autism spectrum disorder	Vitamin B6	2	-0.0041	0.0016	-0.0052	0.0020	0.3402	0.0018	0.0108
Autism spectrum disorder	Vitamin B6	3	-0.0053	0.0016	-0.0068	0.0020	0.3396	0.0018	0.0008

Autism spectrum disorder	Vitamin B6	4	-0.0052	0.0016	-0.0067	0.0020	0.3402	0.0018	0.0011
Autism spectrum disorder	Vitamin B6	5	-0.0040	0.0016	-0.0051	0.0020	0.3397	0.0018	0.0117
Autism spectrum disorder	Vitamin C	0	-0.2774	0.2346	-0.0025	0.0021	0.3963	0.0024	0.2370
Autism spectrum disorder	Vitamin C	1	-0.2708	0.2335	-0.0024	0.0021	0.3996	0.0024	0.2461
Autism spectrum disorder	Vitamin C	2	-0.4862	0.2319	-0.0044	0.0021	0.3995	0.0024	0.0361
Autism spectrum disorder	Vitamin C	3	-0.2527	0.2315	-0.0023	0.0021	0.3971	0.0024	0.2751
Autism spectrum disorder	Vitamin C	4	-0.2131	0.2328	-0.0019	0.0021	0.3996	0.0023	0.3601
Autism spectrum disorder	Vitamin C	5	-0.4332	0.2296	-0.0039	0.0021	0.3972	0.0024	0.0592
Autism spectrum disorder	Vitamin D	0	-0.0005	0.0060	-0.0001	0.0019	0.1266	0.0033	0.9395
Autism spectrum disorder	Vitamin D	1	0.0002	0.0060	0.0001	0.0019	0.1267	0.0033	0.9731
Autism spectrum disorder	Vitamin D	2	-0.0014	0.0060	-0.0004	0.0019	0.1270	0.0033	0.8113
Autism spectrum disorder	Vitamin D	3	0.0001	0.0060	0.0000	0.0019	0.1269	0.0033	0.9821
Autism spectrum disorder	Vitamin D	4	0.0011	0.0060	0.0003	0.0019	0.1268	0.0033	0.8564
Autism spectrum disorder	Vitamin D	5	-0.0007	0.0060	-0.0002	0.0019	0.1271	0.0033	0.9013
Autism spectrum disorder	Vitamin E	0	0.0331	0.0097	0.0069	0.0020	0.2960	0.0021	0.0007
Autism spectrum disorder	Vitamin E	1	0.0326	0.0097	0.0068	0.0020	0.2956	0.0021	0.0008
Autism spectrum disorder	Vitamin E	2	0.0218	0.0096	0.0046	0.0020	0.2956	0.0021	0.0233
Autism spectrum disorder	Vitamin E	3	0.0318	0.0096	0.0067	0.0020	0.2948	0.0021	0.0009
Autism spectrum disorder	Vitamin E	4	0.0324	0.0097	0.0068	0.0020	0.2956	0.0021	0.0008
Autism spectrum disorder	Vitamin E	5	0.0197	0.0095	0.0041	0.0020	0.2952	0.0021	0.0390
Bipolar disorder	Alcohol	0	0.1965	0.0497	0.0086	0.0022	0.4792	0.0020	0.0001
Bipolar disorder	Alcohol	1	0.1702	0.0491	0.0075	0.0022	0.4965	0.0019	0.0005
Bipolar disorder	Alcohol	2	0.1473	0.0490	0.0065	0.0022	0.4966	0.0019	0.0027
Bipolar disorder	Alcohol	3	0.1734	0.0491	0.0076	0.0022	0.4973	0.0019	0.0004
Bipolar disorder	Alcohol	4	0.0348	0.0404	0.0015	0.0018	0.4962	0.0020	0.3889
Bipolar disorder	Alcohol	5	0.0590	0.0403	0.0026	0.0018	0.4971	0.0020	0.1436
Bipolar disorder	Calcium	0	2.5148	0.8204	0.0065	0.0021	0.3396	0.0022	0.0022
Bipolar disorder	Calcium	1	2.6081	0.8200	0.0067	0.0021	0.3397	0.0022	0.0015
Bipolar disorder	Calcium	2	2.3012	0.8196	0.0060	0.0021	0.3395	0.0022	0.0050
Bipolar disorder	Calcium	3	2.7371	0.8169	0.0071	0.0021	0.3393	0.0021	0.0008
Bipolar disorder	Calcium	4	2.9420	0.8163	0.0076	0.0021	0.3397	0.0022	0.0003
Bipolar disorder	Calcium	5	2.6410	0.8125	0.0068	0.0021	0.3392	0.0021	0.0012
Bipolar disorder	Carbohydrate	0	0.4034	0.1883	0.0046	0.0021	0.4092	0.0019	0.0321
Bipolar disorder	Carbohydrate	1	0.4289	0.1880	0.0049	0.0021	0.4099	0.0019	0.0225
Bipolar disorder	Carbohydrate	2	0.3649	0.1880	0.0042	0.0021	0.4098	0.0019	0.0522
Bipolar disorder	Carbohydrate	3	0.4649	0.1871	0.0053	0.0021	0.4095	0.0019	0.0130
Bipolar disorder	Carbohydrate	4	0.5457	0.1860	0.0062	0.0021	0.4096	0.0019	0.0034
Bipolar disorder	Carbohydrate	5	0.4723	0.1850	0.0054	0.0021	0.4091	0.0019	0.0107

Bipolar disorder	Carotene	0	27.6176	5.8286	0.0097	0.0020	0.2547	0.0026	0.0000
Bipolar disorder	Carotene	1	29.5350	5.7604	0.0103	0.0020	0.2604	0.0026	0.0000
Bipolar disorder	Carotene	2	26.2926	5.7565	0.0092	0.0020	0.2604	0.0026	0.0000
Bipolar disorder	Carotene	3	30.4008	5.7090	0.0106	0.0020	0.2573	0.0026	0.0000
Bipolar disorder	Carotene	4	30.8575	5.7561	0.0108	0.0020	0.2603	0.0026	0.0000
Bipolar disorder	Carotene	5	28.3362	5.7022	0.0099	0.0020	0.2574	0.0026	0.0000
Bipolar disorder	Fibre	0	0.1059	0.0155	0.0151	0.0022	0.4361	0.0016	0.0000
Bipolar disorder	Fibre	1	0.1113	0.0153	0.0159	0.0022	0.4391	0.0016	0.0000
Bipolar disorder	Fibre	2	0.0984	0.0153	0.0140	0.0022	0.4388	0.0016	0.0000
Bipolar disorder	Fibre	3	0.1137	0.0152	0.0162	0.0022	0.4362	0.0016	0.0000
Bipolar disorder	Fibre	4	0.1206	0.0152	0.0172	0.0022	0.4393	0.0016	0.0000
Bipolar disorder	Fibre	5	0.1082	0.0150	0.0154	0.0021	0.4364	0.0016	0.0000
Bipolar disorder	Fat	0	0.2352	0.0666	0.0074	0.0021	0.3347	0.0020	0.0004
Bipolar disorder	Fat	1	0.2270	0.0660	0.0071	0.0021	0.3411	0.0020	0.0006
Bipolar disorder	Fat	2	0.2038	0.0661	0.0064	0.0021	0.3411	0.0020	0.0020
Bipolar disorder	Fat	3	0.2416	0.0659	0.0076	0.0021	0.3411	0.0020	0.0002
Bipolar disorder	Fat	4	0.2251	0.0660	0.0071	0.0021	0.3410	0.0020	0.0006
Bipolar disorder	Fat	5	0.2133	0.0658	0.0067	0.0021	0.3411	0.0020	0.0012
Bipolar disorder	Folate	0	1.4521	0.2576	0.0122	0.0022	0.4061	0.0017	0.0000
Bipolar disorder	Folate	1	1.5160	0.2562	0.0128	0.0022	0.4069	0.0017	0.0000
Bipolar disorder	Folate	2	1.3566	0.2558	0.0114	0.0022	0.4068	0.0017	0.0000
Bipolar disorder	Folate	3	1.5648	0.2541	0.0132	0.0021	0.4051	0.0017	0.0000
Bipolar disorder	Folate	4	1.5619	0.2559	0.0132	0.0022	0.4069	0.0017	0.0000
Bipolar disorder	Folate	5	1.4462	0.2534	0.0122	0.0021	0.4050	0.0017	0.0000
Bipolar disorder	Food weight	0	11.4014	1.8484	0.0141	0.0023	0.5365	0.0015	0.0000
Bipolar disorder	Food weight	1	11.3592	1.8465	0.0140	0.0023	0.5359	0.0015	0.0000
Bipolar disorder	Food weight	2	10.3012	1.8448	0.0127	0.0023	0.5359	0.0015	0.0000
Bipolar disorder	Food weight	3	11.6904	1.8220	0.0145	0.0023	0.5341	0.0015	0.0000
Bipolar disorder	Food weight	4	9.7815	1.8269	0.0121	0.0023	0.5357	0.0015	0.0000
Bipolar disorder	Food weight	5	9.3580	1.8018	0.0116	0.0022	0.5339	0.0015	0.0000
Bipolar disorder	Iron	0	0.0647	0.0099	0.0138	0.0021	0.3692	0.0017	0.0000
Bipolar disorder	Iron	1	0.0652	0.0099	0.0140	0.0021	0.3691	0.0017	0.0000
Bipolar disorder	Iron	2	0.0514	0.0098	0.0110	0.0021	0.3691	0.0017	0.0000
Bipolar disorder	Iron	3	0.0670	0.0099	0.0143	0.0021	0.3683	0.0017	0.0000
Bipolar disorder	Iron	4	0.0619	0.0098	0.0132	0.0021	0.3691	0.0017	0.0000
Bipolar disorder	Iron	5	0.0521	0.0097	0.0111	0.0021	0.3685	0.0017	0.0000
Bipolar disorder	Protein	0	0.1452	0.0554	0.0054	0.0021	0.2924	0.0023	0.0088
Bipolar disorder	Protein	1	0.1497	0.0553	0.0056	0.0021	0.2917	0.0023	0.0068

Bipolar disorder	Protein	2	0.1526	0.0553	0.0057	0.0021	0.2918	0.0023	0.0058
Bipolar disorder	Protein	3	0.1595	0.0551	0.0059	0.0020	0.2918	0.0023	0.0038
Bipolar disorder	Protein	4	0.1523	0.0553	0.0056	0.0020	0.2916	0.0023	0.0058
Bipolar disorder	Protein	5	0.1633	0.0551	0.0061	0.0020	0.2919	0.0023	0.0030
Bipolar disorder	Vitamin B12	0	0.0349	0.0092	0.0074	0.0019	0.1574	0.0030	0.0001
Bipolar disorder	Vitamin B12	1	0.0360	0.0092	0.0076	0.0019	0.1566	0.0030	0.0001
Bipolar disorder	Vitamin B12	2	0.0319	0.0092	0.0067	0.0019	0.1570	0.0030	0.0005
Bipolar disorder	Vitamin B12	3	0.0365	0.0092	0.0077	0.0019	0.1568	0.0030	0.0001
Bipolar disorder	Vitamin B12	4	0.0358	0.0092	0.0076	0.0019	0.1567	0.0030	0.0001
Bipolar disorder	Vitamin B12	5	0.0328	0.0091	0.0069	0.0019	0.1571	0.0030	0.0003
Bipolar disorder	Vitamin B6	0	0.0032	0.0017	0.0041	0.0021	0.3391	0.0018	0.0513
Bipolar disorder	Vitamin B6	1	0.0037	0.0016	0.0047	0.0021	0.3402	0.0018	0.0246
Bipolar disorder	Vitamin B6	2	0.0047	0.0016	0.0060	0.0021	0.3402	0.0018	0.0041
Bipolar disorder	Vitamin B6	3	0.0040	0.0016	0.0051	0.0021	0.3396	0.0018	0.0148
Bipolar disorder	Vitamin B6	4	0.0039	0.0016	0.0050	0.0021	0.3402	0.0018	0.0180
Bipolar disorder	Vitamin B6	5	0.0051	0.0016	0.0065	0.0021	0.3397	0.0018	0.0017
Bipolar disorder	Vitamin C	0	0.8297	0.2409	0.0075	0.0022	0.3963	0.0024	0.0006
Bipolar disorder	Vitamin C	1	0.8925	0.2397	0.0081	0.0022	0.3996	0.0024	0.0002
Bipolar disorder	Vitamin C	2	0.5796	0.2381	0.0052	0.0022	0.3995	0.0024	0.0149
Bipolar disorder	Vitamin C	3	0.9199	0.2376	0.0083	0.0021	0.3971	0.0024	0.0001
Bipolar disorder	Vitamin C	4	0.9442	0.2390	0.0085	0.0022	0.3996	0.0023	0.0001
Bipolar disorder	Vitamin C	5	0.6627	0.2357	0.0060	0.0021	0.3972	0.0024	0.0049
Bipolar disorder	Vitamin D	0	0.0214	0.0061	0.0066	0.0019	0.1265	0.0033	0.0005
Bipolar disorder	Vitamin D	1	0.0220	0.0061	0.0068	0.0019	0.1267	0.0033	0.0003
Bipolar disorder	Vitamin D	2	0.0196	0.0061	0.0061	0.0019	0.1270	0.0033	0.0014
Bipolar disorder	Vitamin D	3	0.0224	0.0061	0.0070	0.0019	0.1269	0.0033	0.0003
Bipolar disorder	Vitamin D	4	0.0223	0.0061	0.0069	0.0019	0.1268	0.0033	0.0003
Bipolar disorder	Vitamin D	5	0.0205	0.0061	0.0064	0.0019	0.1271	0.0033	0.0008
Bipolar disorder	Vitamin E	0	0.0484	0.0100	0.0102	0.0021	0.2960	0.0021	0.0000
Bipolar disorder	Vitamin E	1	0.0492	0.0099	0.0103	0.0021	0.2955	0.0021	0.0000
Bipolar disorder	Vitamin E	2	0.0357	0.0099	0.0075	0.0021	0.2956	0.0021	0.0003
Bipolar disorder	Vitamin E	3	0.0514	0.0099	0.0108	0.0021	0.2947	0.0021	0.0000
Bipolar disorder	Vitamin E	4	0.0527	0.0099	0.0111	0.0021	0.2956	0.0021	0.0000
Bipolar disorder	Vitamin E	5	0.0410	0.0098	0.0086	0.0021	0.2951	0.0021	0.0000
BMI	Alcohol	0	-0.1582	0.0489	-0.0070	0.0022	0.4792	0.0020	0.0012
BMI	Alcohol	1	-0.1183	0.0483	-0.0052	0.0021	0.4965	0.0019	0.0143
BMI	Alcohol	2	-0.0841	0.0483	-0.0037	0.0021	0.4966	0.0019	0.0817
BMI	Alcohol	3	-0.1178	0.0483	-0.0052	0.0021	0.4973	0.0019	0.0147

BMI	Alcohol	4	0.2754	0.0398	0.0121	0.0018	0.4962	0.0020	0.0000
BMI	Alcohol	5	0.2452	0.0398	0.0108	0.0017	0.4971	0.0020	0.0000
BMI	Calcium	0	-1.2311	0.8066	-0.0032	0.0021	0.3396	0.0022	0.1270
BMI	Calcium	1	-0.9602	0.8070	-0.0025	0.0021	0.3397	0.0022	0.2341
BMI	Calcium	2	-0.3826	0.8070	-0.0010	0.0021	0.3395	0.0022	0.6354
BMI	Calcium	3	-0.9940	0.8040	-0.0026	0.0021	0.3393	0.0021	0.2163
BMI	Calcium	4	-1.6285	0.8042	-0.0042	0.0021	0.3396	0.0022	0.0429
BMI	Calcium	5	-1.1459	0.8007	-0.0030	0.0021	0.3392	0.0021	0.1524
BMI	Carbohydrate	0	-1.9025	0.1850	-0.0217	0.0021	0.4091	0.0019	0.0000
BMI	Carbohydrate	1	-1.6930	0.1849	-0.0193	0.0021	0.4098	0.0019	0.0000
BMI	Carbohydrate	2	-1.5975	0.1850	-0.0182	0.0021	0.4097	0.0019	0.0000
BMI	Carbohydrate	3	-1.7054	0.1841	-0.0195	0.0021	0.4094	0.0019	0.0000
BMI	Carbohydrate	4	-1.9926	0.1832	-0.0227	0.0021	0.4095	0.0019	0.0000
BMI	Carbohydrate	5	-1.8850	0.1822	-0.0215	0.0021	0.4090	0.0019	0.0000
BMI	Carotene	0	-4.1785	5.7321	-0.0015	0.0020	0.2547	0.0026	0.4660
BMI	Carotene	1	-12.7084	5.6712	-0.0044	0.0020	0.2604	0.0026	0.0250
BMI	Carotene	2	-7.7635	5.6700	-0.0027	0.0020	0.2604	0.0026	0.1709
BMI	Carotene	3	-13.3139	5.6212	-0.0047	0.0020	0.2573	0.0026	0.0179
BMI	Carotene	4	-10.2307	5.6730	-0.0036	0.0020	0.2604	0.0026	0.0713
BMI	Carotene	5	-7.5043	5.6210	-0.0026	0.0020	0.2574	0.0026	0.1819
BMI	Fibre	0	-0.0676	0.0152	-0.0096	0.0022	0.4361	0.0016	0.0000
BMI	Fibre	1	-0.0865	0.0151	-0.0123	0.0021	0.4390	0.0016	0.0000
BMI	Fibre	2	-0.0657	0.0150	-0.0094	0.0021	0.4387	0.0016	0.0000
BMI	Fibre	3	-0.0875	0.0149	-0.0125	0.0021	0.4361	0.0016	0.0000
BMI	Fibre	4	-0.0913	0.0150	-0.0130	0.0021	0.4393	0.0016	0.0000
BMI	Fibre	5	-0.0761	0.0148	-0.0108	0.0021	0.4364	0.0016	0.0000
BMI	Fat	0	-0.5809	0.0655	-0.0182	0.0021	0.3347	0.0020	0.0000
BMI	Fat	1	-0.4345	0.0650	-0.0136	0.0020	0.3411	0.0020	0.0000
BMI	Fat	2	-0.4027	0.0650	-0.0126	0.0020	0.3411	0.0020	0.0000
BMI	Fat	3	-0.4303	0.0648	-0.0135	0.0020	0.3411	0.0020	0.0000
BMI	Fat	4	-0.4666	0.0650	-0.0146	0.0020	0.3410	0.0020	0.0000
BMI	Fat	5	-0.4325	0.0649	-0.0136	0.0020	0.3411	0.0020	0.0000
BMI	Folate	0	-1.7323	0.2532	-0.0146	0.0021	0.4061	0.0017	0.0000
BMI	Folate	1	-1.8711	0.2521	-0.0158	0.0021	0.4069	0.0017	0.0000
BMI	Folate	2	-1.5806	0.2518	-0.0133	0.0021	0.4068	0.0017	0.0000
BMI	Folate	3	-1.8911	0.2500	-0.0159	0.0021	0.4050	0.0017	0.0000
BMI	Folate	4	-1.7181	0.2520	-0.0145	0.0021	0.4068	0.0017	0.0000
BMI	Folate	5	-1.5469	0.2497	-0.0130	0.0021	0.4049	0.0017	0.0000

BMI	Food weight	0	9.6689	1.8167	0.0120	0.0022	0.5365	0.0015	0.0000
BMI	Food weight	1	8.5572	1.8163	0.0106	0.0022	0.5359	0.0015	0.0000
BMI	Food weight	2	10.3910	1.8155	0.0129	0.0022	0.5359	0.0015	0.0000
BMI	Food weight	3	8.2259	1.7924	0.0102	0.0022	0.5342	0.0015	0.0000
BMI	Food weight	4	12.3331	1.7989	0.0153	0.0022	0.5357	0.0015	0.0000
BMI	Food weight	5	12.7833	1.7744	0.0158	0.0022	0.5339	0.0015	0.0000
BMI	Iron	0	-0.0956	0.0098	-0.0205	0.0021	0.3691	0.0017	0.0000
BMI	Iron	1	-0.0932	0.0098	-0.0199	0.0021	0.3691	0.0017	0.0000
BMI	Iron	2	-0.0708	0.0097	-0.0151	0.0021	0.3691	0.0017	0.0000
BMI	Iron	3	-0.0928	0.0097	-0.0198	0.0021	0.3683	0.0017	0.0000
BMI	Iron	4	-0.0653	0.0097	-0.0140	0.0021	0.3691	0.0017	0.0000
BMI	Iron	5	-0.0518	0.0096	-0.0111	0.0020	0.3685	0.0017	0.0000
BMI	Protein	0	0.0983	0.0545	0.0036	0.0020	0.2924	0.0023	0.0713
BMI	Protein	1	0.1631	0.0544	0.0060	0.0020	0.2917	0.0023	0.0027
BMI	Protein	2	0.1794	0.0544	0.0067	0.0020	0.2918	0.0023	0.0010
BMI	Protein	3	0.1651	0.0543	0.0061	0.0020	0.2918	0.0023	0.0024
BMI	Protein	4	0.1681	0.0544	0.0062	0.0020	0.2916	0.0023	0.0020
BMI	Protein	5	0.1770	0.0543	0.0066	0.0020	0.2919	0.0023	0.0011
BMI	Vitamin B12	0	-0.0055	0.0090	-0.0012	0.0019	0.1574	0.0030	0.5434
BMI	Vitamin B12	1	0.0033	0.0090	0.0007	0.0019	0.1566	0.0030	0.7145
BMI	Vitamin B12	2	0.0118	0.0090	0.0025	0.0019	0.1570	0.0030	0.1929
BMI	Vitamin B12	3	0.0037	0.0090	0.0008	0.0019	0.1568	0.0030	0.6847
BMI	Vitamin B12	4	0.0113	0.0090	0.0024	0.0019	0.1567	0.0030	0.2091
BMI	Vitamin B12	5	0.0166	0.0090	0.0035	0.0019	0.1572	0.0030	0.0660
BMI	Vitamin B6	0	-0.0006	0.0016	-0.0008	0.0021	0.3391	0.0018	0.6997
BMI	Vitamin B6	1	-0.0003	0.0016	-0.0004	0.0021	0.3402	0.0018	0.8308
BMI	Vitamin B6	2	-0.0007	0.0016	-0.0009	0.0021	0.3402	0.0018	0.6714
BMI	Vitamin B6	3	-0.0004	0.0016	-0.0005	0.0021	0.3396	0.0018	0.7943
BMI	Vitamin B6	4	0.0004	0.0016	0.0005	0.0021	0.3402	0.0018	0.7931
BMI	Vitamin B6	5	-0.0003	0.0016	-0.0004	0.0020	0.3397	0.0018	0.8616
BMI	Vitamin C	0	-0.9523	0.2368	-0.0086	0.0021	0.3963	0.0024	0.0001
BMI	Vitamin C	1	-1.1162	0.2358	-0.0101	0.0021	0.3996	0.0024	0.0000
BMI	Vitamin C	2	-0.6067	0.2344	-0.0055	0.0021	0.3995	0.0024	0.0096
BMI	Vitamin C	3	-1.1408	0.2338	-0.0103	0.0021	0.3971	0.0024	0.0000
BMI	Vitamin C	4	-0.8449	0.2354	-0.0076	0.0021	0.3996	0.0023	0.0003
BMI	Vitamin C	5	-0.5165	0.2322	-0.0047	0.0021	0.3972	0.0024	0.0261
BMI	Vitamin D	0	-0.0100	0.0060	-0.0031	0.0019	0.1266	0.0033	0.0988
BMI	Vitamin D	1	-0.0084	0.0060	-0.0026	0.0019	0.1268	0.0033	0.1628

BMI	Vitamin D	2	-0.0037	0.0060	-0.0012	0.0019	0.1270	0.0033	0.5394
BMI	Vitamin D	3	-0.0084	0.0060	-0.0026	0.0019	0.1269	0.0033	0.1638
BMI	Vitamin D	4	-0.0045	0.0060	-0.0014	0.0019	0.1268	0.0033	0.4526
BMI	Vitamin D	5	-0.0016	0.0060	-0.0005	0.0019	0.1271	0.0033	0.7949
BMI	Vitamin E	0	-0.0791	0.0098	-0.0166	0.0021	0.2960	0.0021	0.0000
BMI	Vitamin E	1	-0.0848	0.0098	-0.0178	0.0021	0.2955	0.0021	0.0000
BMI	Vitamin E	2	-0.0641	0.0097	-0.0134	0.0020	0.2956	0.0021	0.0000
BMI	Vitamin E	3	-0.0844	0.0097	-0.0177	0.0020	0.2947	0.0021	0.0000
BMI	Vitamin E	4	-0.0816	0.0098	-0.0171	0.0020	0.2956	0.0021	0.0000
BMI	Vitamin E	5	-0.0655	0.0097	-0.0137	0.0020	0.2951	0.0021	0.0000
Educational attainment	Alcohol	0	0.2015	0.0504	0.0089	0.0022	0.4792	0.0020	0.0001
Educational attainment	Alcohol	1	0.1373	0.0498	0.0060	0.0022	0.4965	0.0019	0.0058
Educational attainment	Alcohol	2	0.0381	0.0502	0.0017	0.0022	0.4966	0.0019	0.4483
Educational attainment	Alcohol	3	0.1378	0.0498	0.0061	0.0022	0.4973	0.0019	0.0056
Educational attainment	Alcohol	4	-0.2785	0.0410	-0.0123	0.0018	0.4962	0.0020	0.0000
Educational attainment	Alcohol	5	-0.1748	0.0413	-0.0077	0.0018	0.4970	0.0020	0.0000
Educational attainment	Calcium	0	5.3160	0.8320	0.0137	0.0022	0.3396	0.0022	0.0000
Educational attainment	Calcium	1	5.3479	0.8317	0.0138	0.0022	0.3397	0.0022	0.0000
Educational attainment	Calcium	2	3.7451	0.8387	0.0097	0.0022	0.3395	0.0022	0.0000
Educational attainment	Calcium	3	5.3077	0.8288	0.0137	0.0021	0.3393	0.0021	0.0000
Educational attainment	Calcium	4	6.0451	0.8289	0.0156	0.0021	0.3397	0.0022	0.0000
Educational attainment	Calcium	5	4.1939	0.8317	0.0108	0.0022	0.3392	0.0021	0.0000
Educational attainment	Carbohydrate	0	1.4718	0.1909	0.0168	0.0022	0.4092	0.0019	0.0000
Educational attainment	Carbohydrate	1	1.4156	0.1906	0.0161	0.0022	0.4099	0.0019	0.0000
Educational attainment	Carbohydrate	2	1.1361	0.1923	0.0130	0.0022	0.4098	0.0019	0.0000
Educational attainment	Carbohydrate	3	1.4327	0.1898	0.0163	0.0022	0.4095	0.0019	0.0000
Educational attainment	Carbohydrate	4	1.7397	0.1889	0.0198	0.0022	0.4096	0.0019	0.0000
Educational attainment	Carbohydrate	5	1.3309	0.1894	0.0152	0.0022	0.4091	0.0019	0.0000
Educational attainment	Carotene	0	22.3627	5.9125	0.0078	0.0021	0.2548	0.0026	0.0002
Educational attainment	Carotene	1	29.6276	5.8442	0.0104	0.0020	0.2604	0.0026	0.0000
Educational attainment	Carotene	2	14.6732	5.8919	0.0051	0.0021	0.2604	0.0026	0.0128
Educational attainment	Carotene	3	29.2274	5.7938	0.0102	0.0020	0.2573	0.0026	0.0000
Educational attainment	Carotene	4	26.9428	5.8465	0.0094	0.0020	0.2604	0.0026	0.0000
Educational attainment	Carotene	5	13.7026	5.8380	0.0048	0.0020	0.2574	0.0026	0.0189
Educational attainment	Fibre	0	0.1418	0.0157	0.0202	0.0022	0.4361	0.0016	0.0000
Educational attainment	Fibre	1	0.1598	0.0155	0.0228	0.0022	0.4390	0.0016	0.0000
Educational attainment	Fibre	2	0.0993	0.0156	0.0141	0.0022	0.4388	0.0016	0.0000
Educational attainment	Fibre	3	0.1581	0.0154	0.0225	0.0022	0.4362	0.0016	0.0000

Educational attainment	Fibre	4	0.1646	0.0154	0.0235	0.0022	0.4393	0.0016	0.0000
Educational attainment	Fibre	5	0.1037	0.0154	0.0148	0.0022	0.4364	0.0016	0.0000
Educational attainment	Fat	0	0.5635	0.0675	0.0177	0.0021	0.3348	0.0020	0.0000
Educational attainment	Fat	1	0.4797	0.0670	0.0151	0.0021	0.3411	0.0020	0.0000
Educational attainment	Fat	2	0.3880	0.0676	0.0122	0.0021	0.3412	0.0020	0.0000
Educational attainment	Fat	3	0.4774	0.0668	0.0150	0.0021	0.3411	0.0020	0.0000
Educational attainment	Fat	4	0.5142	0.0670	0.0161	0.0021	0.3410	0.0020	0.0000
Educational attainment	Fat	5	0.4106	0.0674	0.0129	0.0021	0.3411	0.0020	0.0000
Educational attainment	Folate	0	2.3659	0.2612	0.0199	0.0022	0.4062	0.0017	0.0000
Educational attainment	Folate	1	2.5394	0.2598	0.0214	0.0022	0.4070	0.0017	0.0000
Educational attainment	Folate	2	1.7213	0.2617	0.0145	0.0022	0.4068	0.0017	0.0000
Educational attainment	Folate	3	2.5169	0.2577	0.0212	0.0022	0.4051	0.0017	0.0000
Educational attainment	Folate	4	2.3695	0.2598	0.0200	0.0022	0.4069	0.0017	0.0000
Educational attainment	Folate	5	1.6647	0.2594	0.0140	0.0022	0.4050	0.0017	0.0000
Educational attainment	Food weight	0	11.2671	1.8743	0.0139	0.0023	0.5365	0.0015	0.0000
Educational attainment	Food weight	1	11.7199	1.8726	0.0145	0.0023	0.5359	0.0015	0.0000
Educational attainment	Food weight	2	6.5253	1.8877	0.0081	0.0023	0.5359	0.0015	0.0005
Educational attainment	Food weight	3	11.9948	1.8481	0.0148	0.0023	0.5341	0.0015	0.0000
Educational attainment	Food weight	4	7.6348	1.8549	0.0094	0.0023	0.5357	0.0015	0.0000
Educational attainment	Food weight	5	5.0270	1.8441	0.0062	0.0023	0.5339	0.0015	0.0064
Educational attainment	Iron	0	0.1805	0.0101	0.0386	0.0022	0.3692	0.0017	0.0000
Educational attainment	Iron	1	0.1804	0.0101	0.0386	0.0022	0.3691	0.0017	0.0000
Educational attainment	Iron	2	0.1158	0.0101	0.0248	0.0022	0.3691	0.0017	0.0000
Educational attainment	Iron	3	0.1783	0.0100	0.0381	0.0021	0.3684	0.0017	0.0000
Educational attainment	Iron	4	0.1507	0.0100	0.0322	0.0021	0.3691	0.0017	0.0000
Educational attainment	Iron	5	0.1022	0.0099	0.0219	0.0021	0.3685	0.0017	0.0000
Educational attainment	Protein	0	0.2324	0.0562	0.0086	0.0021	0.2924	0.0023	0.0000
Educational attainment	Protein	1	0.2091	0.0561	0.0078	0.0021	0.2917	0.0023	0.0002
Educational attainment	Protein	2	0.1820	0.0566	0.0068	0.0021	0.2918	0.0023	0.0013
Educational attainment	Protein	3	0.2015	0.0559	0.0075	0.0021	0.2919	0.0023	0.0003
Educational attainment	Protein	4	0.2025	0.0561	0.0075	0.0021	0.2917	0.0023	0.0003
Educational attainment	Protein	5	0.1802	0.0564	0.0067	0.0021	0.2919	0.0023	0.0014
Educational attainment	Vitamin B12	0	0.0552	0.0093	0.0117	0.0020	0.1574	0.0030	0.0000
Educational attainment	Vitamin B12	1	0.0527	0.0093	0.0111	0.0020	0.1566	0.0030	0.0000
Educational attainment	Vitamin B12	2	0.0297	0.0094	0.0063	0.0020	0.1570	0.0030	0.0015
Educational attainment	Vitamin B12	3	0.0515	0.0093	0.0109	0.0020	0.1568	0.0030	0.0000
Educational attainment	Vitamin B12	4	0.0435	0.0093	0.0092	0.0020	0.1567	0.0030	0.0000
Educational attainment	Vitamin B12	5	0.0257	0.0094	0.0054	0.0020	0.1572	0.0030	0.0061

Educational attainment	Vitamin B6	0	-0.0003	0.0017	-0.0004	0.0021	0.3391	0.0018	0.8590
Educational attainment	Vitamin B6	1	0.0004	0.0017	0.0005	0.0021	0.3402	0.0018	0.8212
Educational attainment	Vitamin B6	2	0.0024	0.0017	0.0031	0.0021	0.3402	0.0018	0.1459
Educational attainment	Vitamin B6	3	0.0003	0.0017	0.0004	0.0021	0.3396	0.0018	0.8673
Educational attainment	Vitamin B6	4	-0.0005	0.0017	-0.0006	0.0021	0.3402	0.0018	0.7724
Educational attainment	Vitamin B6	5	0.0020	0.0017	0.0026	0.0021	0.3397	0.0018	0.2210
Educational attainment	Vitamin C	0	2.3076	0.2442	0.0209	0.0022	0.3964	0.0024	0.0000
Educational attainment	Vitamin C	1	2.4917	0.2431	0.0225	0.0022	0.3996	0.0024	0.0000
Educational attainment	Vitamin C	2	0.9933	0.2436	0.0090	0.0022	0.3995	0.0024	0.0000
Educational attainment	Vitamin C	3	2.4702	0.2410	0.0223	0.0022	0.3971	0.0024	0.0000
Educational attainment	Vitamin C	4	2.1965	0.2427	0.0199	0.0022	0.3996	0.0023	0.0000
Educational attainment	Vitamin C	5	0.8980	0.2412	0.0081	0.0022	0.3972	0.0024	0.0002
Educational attainment	Vitamin D	0	0.0362	0.0062	0.0112	0.0019	0.1266	0.0033	0.0000
Educational attainment	Vitamin D	1	0.0365	0.0062	0.0113	0.0019	0.1268	0.0033	0.0000
Educational attainment	Vitamin D	2	0.0234	0.0063	0.0073	0.0020	0.1270	0.0033	0.0002
Educational attainment	Vitamin D	3	0.0361	0.0062	0.0112	0.0019	0.1269	0.0033	0.0000
Educational attainment	Vitamin D	4	0.0322	0.0062	0.0100	0.0019	0.1268	0.0033	0.0000
Educational attainment	Vitamin D	5	0.0217	0.0063	0.0067	0.0019	0.1271	0.0033	0.0005
Educational attainment	Vitamin E	0	0.1385	0.0101	0.0291	0.0021	0.2960	0.0021	0.0000
Educational attainment	Vitamin E	1	0.1425	0.0101	0.0299	0.0021	0.2955	0.0021	0.0000
Educational attainment	Vitamin E	2	0.0815	0.0101	0.0171	0.0021	0.2956	0.0021	0.0000
Educational attainment	Vitamin E	3	0.1405	0.0100	0.0295	0.0021	0.2947	0.0021	0.0000
Educational attainment	Vitamin E	4	0.1389	0.0101	0.0291	0.0021	0.2956	0.0021	0.0000
Educational attainment	Vitamin E	5	0.0810	0.0100	0.0170	0.0021	0.2951	0.0021	0.0000
Food addiction	Alcohol	0	-0.0378	0.0487	-0.0017	0.0021	0.4792	0.0020	0.4380
Food addiction	Alcohol	1	-0.0406	0.0481	-0.0018	0.0021	0.4965	0.0019	0.3989
Food addiction	Alcohol	2	-0.0392	0.0481	-0.0017	0.0021	0.4966	0.0019	0.4145
Food addiction	Alcohol	3	-0.0410	0.0481	-0.0018	0.0021	0.4973	0.0019	0.3944
Food addiction	Alcohol	4	0.0125	0.0396	0.0005	0.0017	0.4962	0.0020	0.7527
Food addiction	Alcohol	5	0.0099	0.0395	0.0004	0.0017	0.4971	0.0020	0.8012
Food addiction	Calcium	0	1.2404	0.8045	0.0032	0.0021	0.3396	0.0022	0.1231
Food addiction	Calcium	1	1.2746	0.8040	0.0033	0.0021	0.3397	0.0022	0.1129
Food addiction	Calcium	2	1.3142	0.8031	0.0034	0.0021	0.3395	0.0022	0.1018
Food addiction	Calcium	3	1.3360	0.8010	0.0035	0.0021	0.3393	0.0021	0.0953
Food addiction	Calcium	4	1.1713	0.8001	0.0030	0.0021	0.3396	0.0022	0.1432
Food addiction	Calcium	5	1.2643	0.7960	0.0033	0.0021	0.3392	0.0021	0.1122
Food addiction	Carbohydrate	0	0.2015	0.1846	0.0023	0.0021	0.4092	0.0019	0.2751
Food addiction	Carbohydrate	1	0.2183	0.1843	0.0025	0.0021	0.4099	0.0019	0.2363

Food addiction	Carbohydrate	2	0.2247	0.1842	0.0026	0.0021	0.4098	0.0019	0.2225
Food addiction	Carbohydrate	3	0.2325	0.1835	0.0027	0.0021	0.4095	0.0019	0.2051
Food addiction	Carbohydrate	4	0.1794	0.1823	0.0020	0.0021	0.4096	0.0019	0.3252
Food addiction	Carbohydrate	5	0.2002	0.1812	0.0023	0.0021	0.4091	0.0019	0.2694
Food addiction	Carotene	0	-0.0774	5.7155	0.0000	0.0020	0.2547	0.0026	0.9892
Food addiction	Carotene	1	-0.1294	5.6486	0.0000	0.0020	0.2604	0.0026	0.9817
Food addiction	Carotene	2	0.1336	5.6413	0.0000	0.0020	0.2604	0.0026	0.9811
Food addiction	Carotene	3	0.3458	5.5980	0.0001	0.0020	0.2573	0.0026	0.9508
Food addiction	Carotene	4	-0.0612	5.6425	0.0000	0.0020	0.2604	0.0026	0.9914
Food addiction	Carotene	5	0.5455	5.5863	0.0002	0.0020	0.2574	0.0026	0.9222
Food addiction	Fibre	0	0.0055	0.0152	0.0008	0.0022	0.4361	0.0016	0.7191
Food addiction	Fibre	1	0.0055	0.0150	0.0008	0.0021	0.4391	0.0016	0.7154
Food addiction	Fibre	2	0.0067	0.0150	0.0010	0.0021	0.4388	0.0016	0.6531
Food addiction	Fibre	3	0.0067	0.0149	0.0010	0.0021	0.4362	0.0016	0.6505
Food addiction	Fibre	4	0.0040	0.0149	0.0006	0.0021	0.4393	0.0016	0.7869
Food addiction	Fibre	5	0.0061	0.0147	0.0009	0.0021	0.4364	0.0016	0.6785
Food addiction	Fat	0	0.1073	0.0653	0.0034	0.0020	0.3347	0.0020	0.1003
Food addiction	Fat	1	0.1140	0.0648	0.0036	0.0020	0.3411	0.0020	0.0783
Food addiction	Fat	2	0.1161	0.0647	0.0036	0.0020	0.3411	0.0020	0.0730
Food addiction	Fat	3	0.1185	0.0646	0.0037	0.0020	0.3411	0.0020	0.0666
Food addiction	Fat	4	0.1115	0.0647	0.0035	0.0020	0.3410	0.0020	0.0847
Food addiction	Fat	5	0.1178	0.0645	0.0037	0.0020	0.3411	0.0020	0.0678
Food addiction	Folate	0	0.0376	0.2526	0.0003	0.0021	0.4061	0.0017	0.8818
Food addiction	Folate	1	0.0427	0.2512	0.0004	0.0021	0.4069	0.0017	0.8650
Food addiction	Folate	2	0.0632	0.2507	0.0005	0.0021	0.4068	0.0017	0.8009
Food addiction	Folate	3	0.0642	0.2491	0.0005	0.0021	0.4051	0.0017	0.7967
Food addiction	Folate	4	0.0476	0.2508	0.0004	0.0021	0.4069	0.0017	0.8493
Food addiction	Folate	5	0.0817	0.2483	0.0007	0.0021	0.4050	0.0017	0.7421
Food addiction	Food weight	0	-0.1379	1.8126	-0.0002	0.0022	0.5365	0.0015	0.9394
Food addiction	Food weight	1	-0.2058	1.8107	-0.0003	0.0022	0.5359	0.0015	0.9095
Food addiction	Food weight	2	-0.1056	1.8079	-0.0001	0.0022	0.5359	0.0015	0.9534
Food addiction	Food weight	3	-0.0779	1.7866	-0.0001	0.0022	0.5341	0.0015	0.9652
Food addiction	Food weight	4	0.2036	1.7908	0.0003	0.0022	0.5357	0.0015	0.9095
Food addiction	Food weight	5	0.3636	1.7652	0.0004	0.0022	0.5339	0.0015	0.8368
Food addiction	Iron	0	0.0002	0.0097	0.0000	0.0021	0.3692	0.0017	0.9853
Food addiction	Iron	1	0.0004	0.0097	0.0001	0.0021	0.3691	0.0017	0.9635
Food addiction	Iron	2	0.0019	0.0096	0.0004	0.0021	0.3691	0.0017	0.8463
Food addiction	Iron	3	0.0010	0.0097	0.0002	0.0021	0.3684	0.0017	0.9189

Food addiction	Iron	4	0.0030	0.0096	0.0006	0.0021	0.3691	0.0017	0.7536
Food addiction	Iron	5	0.0043	0.0095	0.0009	0.0020	0.3685	0.0017	0.6486
Food addiction	Protein	0	0.0285	0.0543	0.0011	0.0020	0.2924	0.0023	0.6001
Food addiction	Protein	1	0.0332	0.0542	0.0012	0.0020	0.2917	0.0023	0.5405
Food addiction	Protein	2	0.0353	0.0542	0.0013	0.0020	0.2918	0.0023	0.5151
Food addiction	Protein	3	0.0367	0.0541	0.0014	0.0020	0.2918	0.0023	0.4975
Food addiction	Protein	4	0.0314	0.0542	0.0012	0.0020	0.2916	0.0023	0.5625
Food addiction	Protein	5	0.0363	0.0540	0.0013	0.0020	0.2919	0.0023	0.5008
Food addiction	Vitamin B12	0	0.0009	0.0090	0.0002	0.0019	0.1574	0.0030	0.9212
Food addiction	Vitamin B12	1	0.0017	0.0090	0.0004	0.0019	0.1566	0.0030	0.8526
Food addiction	Vitamin B12	2	0.0024	0.0090	0.0005	0.0019	0.1570	0.0030	0.7886
Food addiction	Vitamin B12	3	0.0018	0.0090	0.0004	0.0019	0.1568	0.0030	0.8383
Food addiction	Vitamin B12	4	0.0023	0.0090	0.0005	0.0019	0.1567	0.0030	0.7995
Food addiction	Vitamin B12	5	0.0030	0.0090	0.0006	0.0019	0.1572	0.0030	0.7409
Food addiction	Vitamin B6	0	0.0004	0.0016	0.0005	0.0021	0.3391	0.0018	0.8144
Food addiction	Vitamin B6	1	0.0005	0.0016	0.0006	0.0021	0.3402	0.0018	0.7592
Food addiction	Vitamin B6	2	0.0006	0.0016	0.0007	0.0021	0.3402	0.0018	0.7201
Food addiction	Vitamin B6	3	0.0006	0.0016	0.0008	0.0020	0.3396	0.0018	0.7057
Food addiction	Vitamin B6	4	0.0005	0.0016	0.0006	0.0021	0.3402	0.0018	0.7593
Food addiction	Vitamin B6	5	0.0007	0.0016	0.0009	0.0020	0.3397	0.0018	0.6753
Food addiction	Vitamin C	0	-0.1781	0.2362	-0.0016	0.0021	0.3963	0.0024	0.4507
Food addiction	Vitamin C	1	-0.1752	0.2350	-0.0016	0.0021	0.3996	0.0024	0.4561
Food addiction	Vitamin C	2	-0.1432	0.2333	-0.0013	0.0021	0.3995	0.0024	0.5394
Food addiction	Vitamin C	3	-0.1627	0.2330	-0.0015	0.0021	0.3971	0.0024	0.4849
Food addiction	Vitamin C	4	-0.1575	0.2343	-0.0014	0.0021	0.3996	0.0023	0.5014
Food addiction	Vitamin C	5	-0.1250	0.2309	-0.0011	0.0021	0.3972	0.0024	0.5882
Food addiction	Vitamin D	0	0.0020	0.0060	0.0006	0.0019	0.1266	0.0033	0.7449
Food addiction	Vitamin D	1	0.0022	0.0060	0.0007	0.0019	0.1267	0.0033	0.7139
Food addiction	Vitamin D	2	0.0026	0.0060	0.0008	0.0019	0.1270	0.0033	0.6682
Food addiction	Vitamin D	3	0.0024	0.0060	0.0007	0.0019	0.1269	0.0033	0.6958
Food addiction	Vitamin D	4	0.0024	0.0060	0.0008	0.0019	0.1268	0.0033	0.6853
Food addiction	Vitamin D	5	0.0028	0.0060	0.0009	0.0019	0.1271	0.0033	0.6365
Food addiction	Vitamin E	0	0.0054	0.0098	0.0011	0.0020	0.2960	0.0021	0.5795
Food addiction	Vitamin E	1	0.0053	0.0097	0.0011	0.0020	0.2955	0.0021	0.5898
Food addiction	Vitamin E	2	0.0064	0.0097	0.0013	0.0020	0.2956	0.0021	0.5075
Food addiction	Vitamin E	3	0.0061	0.0097	0.0013	0.0020	0.2947	0.0021	0.5322
Food addiction	Vitamin E	4	0.0051	0.0097	0.0011	0.0020	0.2956	0.0021	0.5965
Food addiction	Vitamin E	5	0.0067	0.0096	0.0014	0.0020	0.2951	0.0021	0.4846

Height	Alcohol	0	0.2602	0.0698	0.0114	0.0031	0.4792	0.0020	0.0002
Height	Alcohol	1	0.2480	0.0689	0.0109	0.0030	0.4965	0.0019	0.0003
Height	Alcohol	2	0.2181	0.0688	0.0096	0.0030	0.4966	0.0019	0.0015
Height	Alcohol	3	0.2535	0.0689	0.0112	0.0030	0.4973	0.0019	0.0002
Height	Alcohol	4	0.1222	0.0567	0.0054	0.0025	0.4962	0.0020	0.0311
Height	Alcohol	5	0.1607	0.0566	0.0071	0.0025	0.4971	0.0020	0.0045
Height	Calcium	0	15.6772	1.1509	0.0405	0.0030	0.3396	0.0022	0.0000
Height	Calcium	1	15.6412	1.1502	0.0405	0.0030	0.3397	0.0022	0.0000
Height	Calcium	2	15.1204	1.1495	0.0391	0.0030	0.3395	0.0022	0.0000
Height	Calcium	3	15.8330	1.1459	0.0410	0.0030	0.3393	0.0021	0.0000
Height	Calcium	4	15.8957	1.1447	0.0411	0.0030	0.3397	0.0022	0.0000
Height	Calcium	5	15.5145	1.1393	0.0401	0.0029	0.3392	0.0021	0.0000
Height	Carbohydrate	0	4.5335	0.2640	0.0517	0.0030	0.4091	0.0019	0.0000
Height	Carbohydrate	1	4.4957	0.2636	0.0513	0.0030	0.4099	0.0019	0.0000
Height	Carbohydrate	2	4.4085	0.2636	0.0503	0.0030	0.4097	0.0019	0.0000
Height	Carbohydrate	3	4.5459	0.2624	0.0519	0.0030	0.4094	0.0019	0.0000
Height	Carbohydrate	4	4.6040	0.2608	0.0525	0.0030	0.4096	0.0019	0.0000
Height	Carbohydrate	5	4.5236	0.2593	0.0516	0.0030	0.4090	0.0019	0.0000
Height	Carotene	0	42.2495	8.1802	0.0148	0.0029	0.2548	0.0026	0.0000
Height	Carotene	1	44.3939	8.0844	0.0155	0.0028	0.2604	0.0026	0.0000
Height	Carotene	2	39.9640	8.0774	0.0140	0.0028	0.2604	0.0026	0.0000
Height	Carotene	3	46.1614	8.0123	0.0161	0.0028	0.2573	0.0026	0.0000
Height	Carotene	4	43.1065	8.0763	0.0151	0.0028	0.2604	0.0026	0.0000
Height	Carotene	5	41.2187	7.9992	0.0144	0.0028	0.2574	0.0026	0.0000
Height	Fibre	0	0.2723	0.0218	0.0388	0.0031	0.4361	0.0016	0.0000
Height	Fibre	1	0.2769	0.0215	0.0395	0.0031	0.4390	0.0016	0.0000
Height	Fibre	2	0.2580	0.0214	0.0367	0.0031	0.4388	0.0016	0.0000
Height	Fibre	3	0.2813	0.0213	0.0401	0.0030	0.4361	0.0016	0.0000
Height	Fibre	4	0.2785	0.0213	0.0397	0.0030	0.4393	0.0016	0.0000
Height	Fibre	5	0.2648	0.0210	0.0377	0.0030	0.4364	0.0016	0.0000
Height	Fat	0	1.5465	0.0934	0.0485	0.0029	0.3347	0.0020	0.0000
Height	Fat	1	1.5150	0.0926	0.0475	0.0029	0.3411	0.0020	0.0000
Height	Fat	2	1.4873	0.0926	0.0467	0.0029	0.3412	0.0020	0.0000
Height	Fat	3	1.5253	0.0924	0.0479	0.0029	0.3411	0.0020	0.0000
Height	Fat	4	1.5291	0.0925	0.0480	0.0029	0.3410	0.0020	0.0000
Height	Fat	5	1.5082	0.0923	0.0473	0.0029	0.3411	0.0020	0.0000
Height	Folate	0	4.4914	0.3614	0.0379	0.0030	0.4061	0.0017	0.0000
Height	Folate	1	4.5303	0.3595	0.0382	0.0030	0.4069	0.0017	0.0000

Height	Folate	2	4.2634	0.3588	0.0359	0.0030	0.4068	0.0017	0.0000
Height	Folate	3	4.6126	0.3565	0.0389	0.0030	0.4050	0.0017	0.0000
Height	Folate	4	4.4726	0.3589	0.0377	0.0030	0.4069	0.0017	0.0000
Height	Folate	5	4.3375	0.3554	0.0366	0.0030	0.4049	0.0017	0.0000
Height	Food weight	0	36.6905	2.5932	0.0454	0.0032	0.5365	0.0015	0.0000
Height	Food weight	1	36.9078	2.5905	0.0456	0.0032	0.5359	0.0015	0.0000
Height	Food weight	2	35.3068	2.5878	0.0437	0.0032	0.5359	0.0015	0.0000
Height	Food weight	3	37.6361	2.5560	0.0465	0.0032	0.5341	0.0015	0.0000
Height	Food weight	4	35.5594	2.5624	0.0440	0.0032	0.5357	0.0015	0.0000
Height	Food weight	5	35.4488	2.5267	0.0438	0.0031	0.5338	0.0015	0.0000
Height	Iron	0	0.2309	0.0139	0.0494	0.0030	0.3692	0.0017	0.0000
Height	Iron	1	0.2305	0.0139	0.0493	0.0030	0.3691	0.0017	0.0000
Height	Iron	2	0.2100	0.0138	0.0449	0.0030	0.3691	0.0017	0.0000
Height	Iron	3	0.2329	0.0138	0.0498	0.0030	0.3684	0.0017	0.0000
Height	Iron	4	0.2216	0.0138	0.0474	0.0029	0.3691	0.0017	0.0000
Height	Iron	5	0.2096	0.0136	0.0448	0.0029	0.3685	0.0017	0.0000
Height	Protein	0	0.9176	0.0777	0.0340	0.0029	0.2924	0.0023	0.0000
Height	Protein	1	0.9055	0.0775	0.0336	0.0029	0.2917	0.0023	0.0000
Height	Protein	2	0.8909	0.0775	0.0330	0.0029	0.2918	0.0023	0.0000
Height	Protein	3	0.9166	0.0773	0.0340	0.0029	0.2918	0.0023	0.0000
Height	Protein	4	0.9031	0.0775	0.0335	0.0029	0.2916	0.0023	0.0000
Height	Protein	5	0.9042	0.0773	0.0335	0.0029	0.2919	0.0023	0.0000
Height	Vitamin B12	0	0.0653	0.0129	0.0138	0.0027	0.1574	0.0030	0.0000
Height	Vitamin B12	1	0.0637	0.0129	0.0135	0.0027	0.1566	0.0030	0.0000
Height	Vitamin B12	2	0.0560	0.0129	0.0118	0.0027	0.1570	0.0030	0.0000
Height	Vitamin B12	3	0.0646	0.0129	0.0137	0.0027	0.1568	0.0030	0.0000
Height	Vitamin B12	4	0.0608	0.0128	0.0129	0.0027	0.1567	0.0030	0.0000
Height	Vitamin B12	5	0.0560	0.0128	0.0118	0.0027	0.1572	0.0030	0.0000
Height	Vitamin B6	0	0.0248	0.0023	0.0316	0.0030	0.3390	0.0018	0.0000
Height	Vitamin B6	1	0.0248	0.0023	0.0317	0.0029	0.3402	0.0018	0.0000
Height	Vitamin B6	2	0.0250	0.0023	0.0319	0.0029	0.3402	0.0018	0.0000
Height	Vitamin B6	3	0.0253	0.0023	0.0323	0.0029	0.3396	0.0018	0.0000
Height	Vitamin B6	4	0.0245	0.0023	0.0313	0.0029	0.3402	0.0018	0.0000
Height	Vitamin B6	5	0.0255	0.0023	0.0325	0.0029	0.3396	0.0018	0.0000
Height	Vitamin C	0	2.4836	0.3380	0.0224	0.0031	0.3964	0.0024	0.0000
Height	Vitamin C	1	2.5272	0.3364	0.0228	0.0030	0.3996	0.0024	0.0000
Height	Vitamin C	2	2.0590	0.3341	0.0186	0.0030	0.3995	0.0024	0.0000
Height	Vitamin C	3	2.5906	0.3335	0.0234	0.0030	0.3971	0.0024	0.0000

Height	Vitamin C	4	2.4227	0.3354	0.0219	0.0030	0.3996	0.0023	0.0000
Height	Vitamin C	5	2.0951	0.3306	0.0189	0.0030	0.3972	0.0024	0.0000
Height	Vitamin D	0	0.0290	0.0086	0.0090	0.0027	0.1266	0.0033	0.0008
Height	Vitamin D	1	0.0289	0.0086	0.0090	0.0027	0.1268	0.0033	0.0008
Height	Vitamin D	2	0.0246	0.0086	0.0076	0.0027	0.1270	0.0033	0.0043
Height	Vitamin D	3	0.0295	0.0086	0.0092	0.0027	0.1269	0.0033	0.0006
Height	Vitamin D	4	0.0274	0.0086	0.0085	0.0027	0.1268	0.0033	0.0015
Height	Vitamin D	5	0.0247	0.0086	0.0077	0.0027	0.1271	0.0033	0.0040
Height	Vitamin E	0	0.1903	0.0140	0.0399	0.0029	0.2961	0.0021	0.0000
Height	Vitamin E	1	0.1916	0.0139	0.0402	0.0029	0.2956	0.0021	0.0000
Height	Vitamin E	2	0.1728	0.0138	0.0363	0.0029	0.2956	0.0021	0.0000
Height	Vitamin E	3	0.1940	0.0139	0.0407	0.0029	0.2948	0.0021	0.0000
Height	Vitamin E	4	0.1905	0.0139	0.0400	0.0029	0.2957	0.0021	0.0000
Height	Vitamin E	5	0.1761	0.0137	0.0369	0.0029	0.2952	0.0021	0.0000
Lupus	Alcohol	0	0.0066	0.0495	0.0003	0.0022	0.4792	0.0020	0.8938
Lupus	Alcohol	1	0.0211	0.0489	0.0009	0.0021	0.4965	0.0019	0.6652
Lupus	Alcohol	2	0.0225	0.0488	0.0010	0.0021	0.4966	0.0019	0.6446
Lupus	Alcohol	3	0.0223	0.0488	0.0010	0.0021	0.4973	0.0019	0.6478
Lupus	Alcohol	4	0.0175	0.0402	0.0008	0.0018	0.4962	0.0020	0.6633
Lupus	Alcohol	5	0.0161	0.0401	0.0007	0.0018	0.4971	0.0020	0.6883
Lupus	Calcium	0	0.1014	0.8171	0.0003	0.0021	0.3396	0.0022	0.9013
Lupus	Calcium	1	0.0891	0.8166	0.0002	0.0021	0.3397	0.0022	0.9131
Lupus	Calcium	2	0.1313	0.8157	0.0003	0.0021	0.3395	0.0022	0.8721
Lupus	Calcium	3	0.1584	0.8136	0.0004	0.0021	0.3393	0.0021	0.8456
Lupus	Calcium	4	0.1180	0.8128	0.0003	0.0021	0.3396	0.0022	0.8846
Lupus	Calcium	5	0.2030	0.8086	0.0005	0.0021	0.3392	0.0021	0.8017
Lupus	Carbohydrate	0	-0.1239	0.1875	-0.0014	0.0021	0.4091	0.0019	0.5088
Lupus	Carbohydrate	1	-0.1139	0.1872	-0.0013	0.0021	0.4099	0.0019	0.5429
Lupus	Carbohydrate	2	-0.1072	0.1871	-0.0012	0.0021	0.4098	0.0019	0.5668
Lupus	Carbohydrate	3	-0.1054	0.1863	-0.0012	0.0021	0.4095	0.0019	0.5718
Lupus	Carbohydrate	4	-0.1063	0.1852	-0.0012	0.0021	0.4096	0.0019	0.5659
Lupus	Carbohydrate	5	-0.0937	0.1841	-0.0011	0.0021	0.4091	0.0019	0.6109
Lupus	Carotene	0	-3.8869	5.8061	-0.0014	0.0020	0.2547	0.0026	0.5032
Lupus	Carotene	1	-5.4738	5.7382	-0.0019	0.0020	0.2604	0.0026	0.3401
Lupus	Carotene	2	-5.1585	5.7307	-0.0018	0.0020	0.2604	0.0026	0.3680
Lupus	Carotene	3	-4.7600	5.6870	-0.0017	0.0020	0.2573	0.0026	0.4026
Lupus	Carotene	4	-4.3812	5.7324	-0.0015	0.0020	0.2604	0.0026	0.4447
Lupus	Carotene	5	-3.6416	5.6754	-0.0013	0.0020	0.2574	0.0026	0.5211

Lupus	Fibre	0	-0.0004	0.0154	-0.0001	0.0022	0.4361	0.0016	0.9810
Lupus	Fibre	1	-0.0043	0.0152	-0.0006	0.0022	0.4391	0.0016	0.7781
Lupus	Fibre	2	-0.0029	0.0152	-0.0004	0.0022	0.4388	0.0016	0.8494
Lupus	Fibre	3	-0.0027	0.0151	-0.0004	0.0022	0.4362	0.0016	0.8566
Lupus	Fibre	4	-0.0008	0.0151	-0.0001	0.0022	0.4393	0.0016	0.9588
Lupus	Fibre	5	0.0012	0.0149	0.0002	0.0021	0.4364	0.0016	0.9335
Lupus	Fat	0	-0.1055	0.0663	-0.0033	0.0021	0.3347	0.0020	0.1118
Lupus	Fat	1	-0.0880	0.0658	-0.0028	0.0021	0.3411	0.0020	0.1810
Lupus	Fat	2	-0.0867	0.0657	-0.0027	0.0021	0.3411	0.0020	0.1870
Lupus	Fat	3	-0.0847	0.0656	-0.0027	0.0021	0.3411	0.0020	0.1965
Lupus	Fat	4	-0.0957	0.0657	-0.0030	0.0021	0.3410	0.0020	0.1455
Lupus	Fat	5	-0.0914	0.0655	-0.0029	0.0021	0.3411	0.0020	0.1631
Lupus	Folate	0	0.0185	0.2566	0.0002	0.0022	0.4061	0.0017	0.9424
Lupus	Folate	1	-0.0204	0.2552	-0.0002	0.0022	0.4069	0.0017	0.9364
Lupus	Folate	2	-0.0012	0.2546	0.0000	0.0021	0.4068	0.0017	0.9961
Lupus	Folate	3	0.0075	0.2530	0.0001	0.0021	0.4051	0.0017	0.9763
Lupus	Folate	4	0.0207	0.2548	0.0002	0.0021	0.4069	0.0017	0.9354
Lupus	Folate	5	0.0509	0.2522	0.0004	0.0021	0.4050	0.0017	0.8402
Lupus	Food weight	0	3.9908	1.8406	0.0049	0.0023	0.5365	0.0015	0.0301
Lupus	Food weight	1	3.9027	1.8386	0.0048	0.0023	0.5359	0.0015	0.0338
Lupus	Food weight	2	3.9947	1.8358	0.0049	0.0023	0.5359	0.0015	0.0296
Lupus	Food weight	3	4.0520	1.8143	0.0050	0.0022	0.5341	0.0015	0.0255
Lupus	Food weight	4	3.6901	1.8187	0.0046	0.0022	0.5357	0.0015	0.0425
Lupus	Food weight	5	3.7991	1.7927	0.0047	0.0022	0.0410	0.0015	0.0341
Lupus	Iron	0	-0.0105	0.0099	-0.0022	0.0021	0.3692	0.0017	0.2898
Lupus	Iron	1	-0.0105	0.0099	-0.0022	0.0021	0.3691	0.0017	0.2902
Lupus	Iron	2	-0.0090	0.0098	-0.0019	0.0021	0.3691	0.0017	0.3594
Lupus	Iron	3	-0.0093	0.0098	-0.0020	0.0021	0.3684	0.0017	0.3424
Lupus	Iron	4	-0.0081	0.0098	-0.0017	0.0021	0.3691	0.0017	0.4051
Lupus	Iron	5	-0.0066	0.0097	-0.0014	0.0021	0.3685	0.0017	0.4929
Lupus	Protein	0	-0.0139	0.0552	-0.0005	0.0020	0.2924	0.0023	0.8018
Lupus	Protein	1	-0.0094	0.0551	-0.0003	0.0020	0.2917	0.0023	0.8646
Lupus	Protein	2	-0.0081	0.0550	-0.0003	0.0020	0.2918	0.0023	0.8824
Lupus	Protein	3	-0.0046	0.0549	-0.0002	0.0020	0.2918	0.0023	0.9327
Lupus	Protein	4	-0.0098	0.0550	-0.0004	0.0020	0.2916	0.0023	0.8582
Lupus	Protein	5	-0.0056	0.0548	-0.0002	0.0020	0.2919	0.0023	0.9181
Lupus	Vitamin B12	0	0.0022	0.0092	0.0005	0.0019	0.1574	0.0030	0.8104
Lupus	Vitamin B12	1	0.0026	0.0091	0.0006	0.0019	0.1566	0.0030	0.7732

Lupus	Vitamin B12	2	0.0032	0.0091	0.0007	0.0019	0.1570	0.0030	0.7217
Lupus	Vitamin B12	3	0.0034	0.0091	0.0007	0.0019	0.1568	0.0030	0.7082
Lupus	Vitamin B12	4	0.0034	0.0091	0.0007	0.0019	0.1567	0.0030	0.7093
Lupus	Vitamin B12	5	0.0043	0.0091	0.0009	0.0019	0.1572	0.0030	0.6362
Lupus	Vitamin B6	0	-0.0002	0.0016	-0.0002	0.0021	0.3391	0.0018	0.9234
Lupus	Vitamin B6	1	-0.0003	0.0016	-0.0004	0.0021	0.3402	0.0018	0.8414
Lupus	Vitamin B6	2	-0.0003	0.0016	-0.0004	0.0021	0.3402	0.0018	0.8485
Lupus	Vitamin B6	3	-0.0002	0.0016	-0.0002	0.0021	0.3396	0.0018	0.9190
Lupus	Vitamin B6	4	-0.0002	0.0016	-0.0002	0.0021	0.3402	0.0018	0.9195
Lupus	Vitamin B6	5	-0.0001	0.0016	-0.0001	0.0021	0.3397	0.0018	0.9659
Lupus	Vitamin C	0	0.0543	0.2399	0.0005	0.0022	0.3963	0.0024	0.8209
Lupus	Vitamin C	1	0.0134	0.2387	0.0001	0.0022	0.3996	0.0024	0.9553
Lupus	Vitamin C	2	0.0479	0.2369	0.0004	0.0021	0.3995	0.0024	0.8397
Lupus	Vitamin C	3	0.0394	0.2367	0.0004	0.0021	0.3971	0.0024	0.8679
Lupus	Vitamin C	4	0.0809	0.2380	0.0007	0.0022	0.3996	0.0023	0.7339
Lupus	Vitamin C	5	0.1178	0.2345	0.0011	0.0021	0.3972	0.0024	0.6153
Lupus	Vitamin D	0	-0.0048	0.0061	-0.0015	0.0019	0.1266	0.0033	0.4300
Lupus	Vitamin D	1	-0.0049	0.0061	-0.0015	0.0019	0.1268	0.0033	0.4187
Lupus	Vitamin D	2	-0.0046	0.0061	-0.0014	0.0019	0.1270	0.0033	0.4472
Lupus	Vitamin D	3	-0.0045	0.0061	-0.0014	0.0019	0.1269	0.0033	0.4625
Lupus	Vitamin D	4	-0.0044	0.0061	-0.0014	0.0019	0.1268	0.0033	0.4718
Lupus	Vitamin D	5	-0.0039	0.0061	-0.0012	0.0019	0.1271	0.0033	0.5230
Lupus	Vitamin E	0	-0.0101	0.0099	-0.0021	0.0021	0.2960	0.0021	0.3088
Lupus	Vitamin E	1	-0.0109	0.0099	-0.0023	0.0021	0.2955	0.0021	0.2687
Lupus	Vitamin E	2	-0.0097	0.0098	-0.0020	0.0021	0.2956	0.0021	0.3223
Lupus	Vitamin E	3	-0.0098	0.0098	-0.0021	0.0021	0.2947	0.0021	0.3189
Lupus	Vitamin E	4	-0.0091	0.0099	-0.0019	0.0021	0.2956	0.0021	0.3574
Lupus	Vitamin E	5	-0.0074	0.0098	-0.0016	0.0020	0.2951	0.0021	0.4458
Major depressive disorder	Alcohol	0	0.0179	0.0483	0.0008	0.0021	0.4792	0.0020	0.7111
Major depressive disorder	Alcohol	1	0.0273	0.0477	0.0012	0.0021	0.4965	0.0019	0.5671
Major depressive disorder	Alcohol	2	0.0391	0.0476	0.0017	0.0021	0.4966	0.0019	0.4118
Major depressive disorder	Alcohol	3	0.0281	0.0476	0.0012	0.0021	0.4973	0.0019	0.5551
Major depressive disorder	Alcohol	4	0.1463	0.0393	0.0064	0.0017	0.4962	0.0020	0.0002
Major depressive disorder	Alcohol	5	0.1230	0.0392	0.0054	0.0017	0.4971	0.0020	0.0017
Major depressive disorder	Calcium	0	-1.2795	0.7967	-0.0033	0.0021	0.3396	0.0022	0.1083
Major depressive disorder	Calcium	1	-1.1130	0.7965	-0.0029	0.0021	0.3397	0.0022	0.1623
Major depressive disorder	Calcium	2	-0.6674	0.7961	-0.0017	0.0021	0.3395	0.0022	0.4018
Major depressive disorder	Calcium	3	-1.1052	0.7935	-0.0029	0.0021	0.3393	0.0021	0.1637

Major depressive disorder	Calcium	4	-1.2025	0.7936	-0.0031	0.0021	0.3397	0.0022	0.1297
Major depressive disorder	Calcium	5	-0.8462	0.7897	-0.0022	0.0020	0.3392	0.0021	0.2840
Major depressive disorder	Carbohydrate	0	-0.4699	0.1828	-0.0054	0.0021	0.4091	0.0019	0.0102
Major depressive disorder	Carbohydrate	1	-0.3575	0.1826	-0.0041	0.0021	0.4099	0.0019	0.0502
Major depressive disorder	Carbohydrate	2	-0.2919	0.1826	-0.0033	0.0021	0.4098	0.0019	0.1099
Major depressive disorder	Carbohydrate	3	-0.3604	0.1817	-0.0041	0.0021	0.4095	0.0019	0.0474
Major depressive disorder	Carbohydrate	4	-0.4413	0.1808	-0.0050	0.0021	0.4096	0.0019	0.0147
Major depressive disorder	Carbohydrate	5	-0.3763	0.1798	-0.0043	0.0021	0.4090	0.0019	0.0364
Major depressive disorder	Carotene	0	-10.4537	5.6611	-0.0037	0.0020	0.2548	0.0026	0.0648
Major depressive disorder	Carotene	1	-13.8882	5.5965	-0.0049	0.0020	0.2604	0.0026	0.0131
Major depressive disorder	Carotene	2	-10.8455	5.5922	-0.0038	0.0020	0.2604	0.0026	0.0525
Major depressive disorder	Carotene	3	-13.4905	5.5466	-0.0047	0.0019	0.2573	0.0026	0.0150
Major depressive disorder	Carotene	4	-10.4128	5.5969	-0.0036	0.0020	0.2604	0.0026	0.0628
Major depressive disorder	Carotene	5	-8.4363	5.5432	-0.0029	0.0019	0.2574	0.0026	0.1280
Major depressive disorder	Fibre	0	-0.0190	0.0151	-0.0027	0.0021	0.4361	0.0016	0.2068
Major depressive disorder	Fibre	1	-0.0259	0.0149	-0.0037	0.0021	0.4391	0.0016	0.0809
Major depressive disorder	Fibre	2	-0.0124	0.0148	-0.0018	0.0021	0.4388	0.0016	0.4029
Major depressive disorder	Fibre	3	-0.0249	0.0147	-0.0035	0.0021	0.4362	0.0016	0.0911
Major depressive disorder	Fibre	4	-0.0181	0.0148	-0.0026	0.0021	0.4393	0.0016	0.2206
Major depressive disorder	Fibre	5	-0.0080	0.0146	-0.0011	0.0021	0.4364	0.0016	0.5834
Major depressive disorder	Fat	0	-0.1854	0.0647	-0.0058	0.0020	0.3347	0.0020	0.0041
Major depressive disorder	Fat	1	-0.1164	0.0642	-0.0037	0.0020	0.3411	0.0020	0.0695
Major depressive disorder	Fat	2	-0.1048	0.0642	-0.0033	0.0020	0.3412	0.0020	0.1024
Major depressive disorder	Fat	3	-0.1225	0.0640	-0.0038	0.0020	0.3411	0.0020	0.0556
Major depressive disorder	Fat	4	-0.1493	0.0642	-0.0047	0.0020	0.3410	0.0020	0.0200
Major depressive disorder	Fat	5	-0.1430	0.0640	-0.0045	0.0020	0.3411	0.0020	0.0254
Major depressive disorder	Folate	0	-0.5167	0.2502	-0.0044	0.0021	0.4061	0.0017	0.0389
Major depressive disorder	Folate	1	-0.5577	0.2489	-0.0047	0.0021	0.4069	0.0017	0.0250
Major depressive disorder	Folate	2	-0.3486	0.2484	-0.0029	0.0021	0.4068	0.0017	0.1606
Major depressive disorder	Folate	3	-0.5421	0.2468	-0.0046	0.0021	0.4051	0.0017	0.0281
Major depressive disorder	Folate	4	-0.3931	0.2487	-0.0033	0.0021	0.4069	0.0017	0.1141
Major depressive disorder	Folate	5	-0.2560	0.2463	-0.0022	0.0021	0.4050	0.0017	0.2986
Major depressive disorder	Food weight	0	3.1565	1.7949	0.0039	0.0022	0.5365	0.0015	0.0786
Major depressive disorder	Food weight	1	2.6022	1.7934	0.0032	0.0022	0.5359	0.0015	0.1468
Major depressive disorder	Food weight	2	3.7078	1.7916	0.0046	0.0022	0.5359	0.0015	0.0385
Major depressive disorder	Food weight	3	2.8266	1.7696	0.0035	0.0022	0.5341	0.0015	0.1102
Major depressive disorder	Food weight	4	3.5629	1.7758	0.0044	0.0022	0.5357	0.0015	0.0448
Major depressive disorder	Food weight	5	4.1135	1.7510	0.0051	0.0022	0.5339	0.0015	0.0188

Major depressive disorder	Iron	0	-0.0390	0.0096	-0.0084	0.0021	0.3692	0.0017	0.0001
Major depressive disorder	Iron	1	-0.0376	0.0096	-0.0080	0.0021	0.3691	0.0017	0.0001
Major depressive disorder	Iron	2	-0.0240	0.0096	-0.0051	0.0020	0.3691	0.0017	0.0121
Major depressive disorder	Iron	3	-0.0370	0.0096	-0.0079	0.0021	0.3684	0.0017	0.0001
Major depressive disorder	Iron	4	-0.0223	0.0095	-0.0048	0.0020	0.3691	0.0017	0.0192
Major depressive disorder	Iron	5	-0.0147	0.0094	-0.0032	0.0020	0.3685	0.0017	0.1188
Major depressive disorder	Protein	0	-0.0696	0.0538	-0.0026	0.0020	0.2924	0.0023	0.1960
Major depressive disorder	Protein	1	-0.0364	0.0537	-0.0014	0.0020	0.2917	0.0023	0.4974
Major depressive disorder	Protein	2	-0.0150	0.0537	-0.0006	0.0020	0.2918	0.0023	0.7805
Major depressive disorder	Protein	3	-0.0371	0.0536	-0.0014	0.0020	0.2918	0.0023	0.4880
Major depressive disorder	Protein	4	-0.0323	0.0537	-0.0012	0.0020	0.2917	0.0023	0.5482
Major depressive disorder	Protein	5	-0.0206	0.0536	-0.0008	0.0020	0.2919	0.0023	0.6999
Major depressive disorder	Vitamin B12	0	-0.0202	0.0089	-0.0043	0.0019	0.1574	0.0030	0.0238
Major depressive disorder	Vitamin B12	1	-0.0155	0.0089	-0.0033	0.0019	0.1566	0.0030	0.0819
Major depressive disorder	Vitamin B12	2	-0.0093	0.0089	-0.0020	0.0019	0.1570	0.0030	0.2957
Major depressive disorder	Vitamin B12	3	-0.0152	0.0089	-0.0032	0.0019	0.1568	0.0030	0.0871
Major depressive disorder	Vitamin B12	4	-0.0100	0.0089	-0.0021	0.0019	0.1567	0.0030	0.2625
Major depressive disorder	Vitamin B12	5	-0.0061	0.0089	-0.0013	0.0019	0.1572	0.0030	0.4903
Major depressive disorder	Vitamin B6	0	-0.0038	0.0016	-0.0048	0.0020	0.3391	0.0018	0.0182
Major depressive disorder	Vitamin B6	1	-0.0035	0.0016	-0.0044	0.0020	0.3402	0.0018	0.0295
Major depressive disorder	Vitamin B6	2	-0.0028	0.0016	-0.0036	0.0020	0.3402	0.0018	0.0802
Major depressive disorder	Vitamin B6	3	-0.0034	0.0016	-0.0043	0.0020	0.3396	0.0018	0.0333
Major depressive disorder	Vitamin B6	4	-0.0027	0.0016	-0.0035	0.0020	0.3402	0.0018	0.0901
Major depressive disorder	Vitamin B6	5	-0.0023	0.0016	-0.0030	0.0020	0.3397	0.0018	0.1417
Major depressive disorder	Vitamin C	0	-1.1313	0.2339	-0.0102	0.0021	0.3963	0.0024	0.0000
Major depressive disorder	Vitamin C	1	-1.1871	0.2328	-0.0107	0.0021	0.3996	0.0024	0.0000
Major depressive disorder	Vitamin C	2	-0.8631	0.2312	-0.0078	0.0021	0.3995	0.0024	0.0002
Major depressive disorder	Vitamin C	3	-1.1572	0.2308	-0.0105	0.0021	0.3971	0.0024	0.0000
Major depressive disorder	Vitamin C	4	-0.9192	0.2323	-0.0083	0.0021	0.3996	0.0023	0.0001
Major depressive disorder	Vitamin C	5	-0.6890	0.2291	-0.0062	0.0021	0.3972	0.0024	0.0026
Major depressive disorder	Vitamin D	0	-0.0071	0.0060	-0.0022	0.0019	0.1266	0.0033	0.2364
Major depressive disorder	Vitamin D	1	-0.0061	0.0060	-0.0019	0.0019	0.1268	0.0033	0.3077
Major depressive disorder	Vitamin D	2	-0.0029	0.0060	-0.0009	0.0019	0.1270	0.0033	0.6245
Major depressive disorder	Vitamin D	3	-0.0059	0.0060	-0.0018	0.0019	0.1269	0.0033	0.3204
Major depressive disorder	Vitamin D	4	-0.0030	0.0060	-0.0009	0.0019	0.1268	0.0033	0.6168
Major depressive disorder	Vitamin D	5	-0.0010	0.0060	-0.0003	0.0019	0.1271	0.0033	0.8660
Major depressive disorder	Vitamin E	0	-0.0335	0.0097	-0.0070	0.0020	0.2960	0.0021	0.0005
Major depressive disorder	Vitamin E	1	-0.0359	0.0097	-0.0075	0.0020	0.2956	0.0021	0.0002

Major depressive disorder	Vitamin E	2	-0.0247	0.0096	-0.0052	0.0020	0.2956	0.0021	0.0100
Major depressive disorder	Vitamin E	3	-0.0357	0.0096	-0.0075	0.0020	0.2948	0.0021	0.0002
Major depressive disorder	Vitamin E	4	-0.0299	0.0096	-0.0063	0.0020	0.2957	0.0021	0.0019
Major depressive disorder	Vitamin E	5	-0.0222	0.0095	-0.0046	0.0020	0.2952	0.0021	0.0200
OCD	Alcohol	0	-0.0177	0.0486	-0.0008	0.0021	0.4792	0.0020	0.7159
OCD	Alcohol	1	-0.0236	0.0479	-0.0010	0.0021	0.4965	0.0019	0.6217
OCD	Alcohol	2	-0.0349	0.0479	-0.0015	0.0021	0.4966	0.0019	0.4659
OCD	Alcohol	3	-0.0218	0.0479	-0.0010	0.0021	0.4973	0.0019	0.6490
OCD	Alcohol	4	-0.0613	0.0394	-0.0027	0.0017	0.4962	0.0020	0.1199
OCD	Alcohol	5	-0.0425	0.0394	-0.0019	0.0017	0.4971	0.0020	0.2808
OCD	Calcium	0	2.2635	0.8012	0.0059	0.0021	0.3396	0.0022	0.0047
OCD	Calcium	1	2.2881	0.8008	0.0059	0.0021	0.3397	0.0022	0.0043
OCD	Calcium	2	2.0102	0.8001	0.0052	0.0021	0.3395	0.0022	0.0120
OCD	Calcium	3	2.3399	0.7978	0.0061	0.0021	0.3393	0.0021	0.0034
OCD	Calcium	4	2.2956	0.7969	0.0059	0.0021	0.3396	0.0022	0.0040
OCD	Calcium	5	2.0255	0.7930	0.0052	0.0021	0.3392	0.0021	0.0106
OCD	Carbohydrate	0	0.3445	0.1839	0.0039	0.0021	0.4092	0.0019	0.0610
OCD	Carbohydrate	1	0.3518	0.1836	0.0040	0.0021	0.4099	0.0019	0.0553
OCD	Carbohydrate	2	0.2994	0.1835	0.0034	0.0021	0.4098	0.0019	0.1029
OCD	Carbohydrate	3	0.3596	0.1827	0.0041	0.0021	0.4095	0.0019	0.0491
OCD	Carbohydrate	4	0.3608	0.1816	0.0041	0.0021	0.4096	0.0019	0.0470
OCD	Carbohydrate	5	0.2927	0.1806	0.0033	0.0021	0.4091	0.0019	0.1051
OCD	Carotene	0	8.2268	5.6926	0.0029	0.0020	0.2547	0.0026	0.1484
OCD	Carotene	1	8.5789	5.6259	0.0030	0.0020	0.2604	0.0026	0.1273
OCD	Carotene	2	6.2300	5.6202	0.0022	0.0020	0.2604	0.0026	0.2676
OCD	Carotene	3	9.1079	5.5755	0.0032	0.0019	0.2573	0.0026	0.1024
OCD	Carotene	4	8.2988	5.6199	0.0029	0.0020	0.2604	0.0026	0.1398
OCD	Carotene	5	6.6712	5.5655	0.0023	0.0019	0.2574	0.0026	0.2307
OCD	Fibre	0	0.0591	0.0151	0.0084	0.0022	0.4361	0.0016	0.0001
OCD	Fibre	1	0.0603	0.0149	0.0086	0.0021	0.4390	0.0016	0.0001
OCD	Fibre	2	0.0505	0.0149	0.0072	0.0021	0.4388	0.0016	0.0007
OCD	Fibre	3	0.0617	0.0148	0.0088	0.0021	0.4362	0.0016	0.0000
OCD	Fibre	4	0.0594	0.0148	0.0085	0.0021	0.4393	0.0016	0.0001
OCD	Fibre	5	0.0509	0.0146	0.0072	0.0021	0.4364	0.0016	0.0005
OCD	Fat	0	0.0678	0.0650	0.0021	0.0020	0.3347	0.0020	0.2971
OCD	Fat	1	0.0673	0.0645	0.0021	0.0020	0.3411	0.0020	0.2967
OCD	Fat	2	0.0540	0.0645	0.0017	0.0020	0.3411	0.0020	0.4025
OCD	Fat	3	0.0704	0.0643	0.0022	0.0020	0.3411	0.0020	0.2735

OCD	Fat	4	0.0683	0.0644	0.0021	0.0020	0.3410	0.0020	0.2892
OCD	Fat	5	0.0559	0.0643	0.0018	0.0020	0.3411	0.0020	0.3848
OCD	Folate	0	0.5669	0.2516	0.0048	0.0021	0.4061	0.0017	0.0242
OCD	Folate	1	0.5814	0.2502	0.0049	0.0021	0.4069	0.0017	0.0201
OCD	Folate	2	0.4516	0.2497	0.0038	0.0021	0.4068	0.0017	0.0705
OCD	Folate	3	0.6065	0.2481	0.0051	0.0021	0.4050	0.0017	0.0145
OCD	Folate	4	0.5591	0.2498	0.0047	0.0021	0.4069	0.0017	0.0252
OCD	Folate	5	0.4674	0.2474	0.0039	0.0021	0.4050	0.0017	0.0588
OCD	Food weight	0	1.8289	1.8054	0.0023	0.0022	0.5365	0.0015	0.3111
OCD	Food weight	1	1.8192	1.8035	0.0022	0.0022	0.5359	0.0015	0.3131
OCD	Food weight	2	1.1113	1.8013	0.0014	0.0022	0.5359	0.0015	0.5373
OCD	Food weight	3	1.9960	1.7796	0.0025	0.0022	0.5341	0.0015	0.2620
OCD	Food weight	4	1.5019	1.7838	0.0019	0.0022	0.5357	0.0015	0.3998
OCD	Food weight	5	1.2559	1.7587	0.0016	0.0022	0.5339	0.0015	0.4752
OCD	Iron	0	0.0309	0.0097	0.0066	0.0021	0.3692	0.0017	0.0014
OCD	Iron	1	0.0311	0.0097	0.0066	0.0021	0.3691	0.0017	0.0013
OCD	Iron	2	0.0211	0.0096	0.0045	0.0021	0.3691	0.0017	0.0281
OCD	Iron	3	0.0318	0.0096	0.0068	0.0021	0.3684	0.0017	0.0010
OCD	Iron	4	0.0276	0.0096	0.0059	0.0020	0.3691	0.0017	0.0039
OCD	Iron	5	0.0207	0.0095	0.0044	0.0020	0.3685	0.0017	0.0292
OCD	Protein	0	-0.0092	0.0541	-0.0003	0.0020	0.2924	0.0023	0.8643
OCD	Protein	1	-0.0076	0.0540	-0.0003	0.0020	0.2917	0.0023	0.8886
OCD	Protein	2	-0.0107	0.0540	-0.0004	0.0020	0.2918	0.0023	0.8430
OCD	Protein	3	-0.0043	0.0538	-0.0002	0.0020	0.2918	0.0023	0.9362
OCD	Protein	4	-0.0080	0.0539	-0.0003	0.0020	0.2916	0.0023	0.8816
OCD	Protein	5	-0.0071	0.0538	-0.0003	0.0020	0.2919	0.0023	0.8952
OCD	Vitamin B12	0	0.0134	0.0090	0.0028	0.0019	0.1574	0.0030	0.1356
OCD	Vitamin B12	1	0.0138	0.0090	0.0029	0.0019	0.1566	0.0030	0.1227
OCD	Vitamin B12	2	0.0104	0.0089	0.0022	0.0019	0.1570	0.0030	0.2461
OCD	Vitamin B12	3	0.0143	0.0089	0.0030	0.0019	0.1568	0.0030	0.1096
OCD	Vitamin B12	4	0.0132	0.0089	0.0028	0.0019	0.1567	0.0030	0.1389
OCD	Vitamin B12	5	0.0110	0.0089	0.0023	0.0019	0.1572	0.0030	0.2166
OCD	Vitamin B6	0	0.0008	0.0016	0.0011	0.0021	0.3391	0.0018	0.6069
OCD	Vitamin B6	1	0.0009	0.0016	0.0012	0.0020	0.3402	0.0018	0.5568
OCD	Vitamin B6	2	0.0013	0.0016	0.0016	0.0020	0.3402	0.0018	0.4305
OCD	Vitamin B6	3	0.0011	0.0016	0.0014	0.0020	0.3396	0.0018	0.4997
OCD	Vitamin B6	4	0.0009	0.0016	0.0011	0.0020	0.3402	0.0018	0.5959
OCD	Vitamin B6	5	0.0014	0.0016	0.0017	0.0020	0.3397	0.0018	0.3954

OCD	Vitamin C	0	0.0836	0.2352	0.0008	0.0021	0.3963	0.0024	0.7223
OCD	Vitamin C	1	0.0974	0.2341	0.0009	0.0021	0.3996	0.0024	0.6773
OCD	Vitamin C	2	-0.1318	0.2324	-0.0012	0.0021	0.3995	0.0024	0.5706
OCD	Vitamin C	3	0.1207	0.2321	0.0011	0.0021	0.3971	0.0024	0.6029
OCD	Vitamin C	4	0.0693	0.2334	0.0006	0.0021	0.3996	0.0023	0.7664
OCD	Vitamin C	5	-0.1128	0.2300	-0.0010	0.0021	0.3972	0.0024	0.6240
OCD	Vitamin D	0	0.0142	0.0060	0.0044	0.0019	0.1266	0.0033	0.0178
OCD	Vitamin D	1	0.0144	0.0060	0.0045	0.0019	0.1268	0.0033	0.0163
OCD	Vitamin D	2	0.0125	0.0060	0.0039	0.0019	0.1270	0.0033	0.0367
OCD	Vitamin D	3	0.0147	0.0060	0.0046	0.0019	0.1269	0.0033	0.0142
OCD	Vitamin D	4	0.0140	0.0060	0.0044	0.0019	0.1268	0.0033	0.0191
OCD	Vitamin D	5	0.0128	0.0060	0.0040	0.0019	0.1271	0.0033	0.0325
OCD	Vitamin E	0	0.0321	0.0097	0.0067	0.0020	0.2960	0.0021	0.0010
OCD	Vitamin E	1	0.0322	0.0097	0.0068	0.0020	0.2955	0.0021	0.0009
OCD	Vitamin E	2	0.0233	0.0096	0.0049	0.0020	0.2956	0.0021	0.0158
OCD	Vitamin E	3	0.0330	0.0096	0.0069	0.0020	0.2947	0.0021	0.0006
OCD	Vitamin E	4	0.0311	0.0097	0.0065	0.0020	0.2956	0.0021	0.0013
OCD	Vitamin E	5	0.0235	0.0096	0.0049	0.0020	0.2951	0.0021	0.0142
Persistent thinness	Alcohol	0	-0.1009	0.0571	-0.0044	0.0025	0.4792	0.0020	0.0772
Persistent thinness	Alcohol	1	-0.1016	0.0563	-0.0045	0.0025	0.4965	0.0019	0.0713
Persistent thinness	Alcohol	2	-0.1041	0.0563	-0.0046	0.0025	0.4966	0.0019	0.0644
Persistent thinness	Alcohol	3	-0.1012	0.0563	-0.0045	0.0025	0.4973	0.0019	0.0724
Persistent thinness	Alcohol	4	-0.1489	0.0464	-0.0066	0.0020	0.4962	0.0020	0.0013
Persistent thinness	Alcohol	5	-0.1504	0.0463	-0.0066	0.0020	0.4971	0.0020	0.0012
Persistent thinness	Calcium	0	-0.5861	0.9423	-0.0015	0.0024	0.3396	0.0022	0.5340
Persistent thinness	Calcium	1	-0.6485	0.9417	-0.0017	0.0024	0.3397	0.0022	0.4910
Persistent thinness	Calcium	2	-0.6161	0.9407	-0.0016	0.0024	0.3395	0.0022	0.5125
Persistent thinness	Calcium	3	-0.6243	0.9382	-0.0016	0.0024	0.3393	0.0021	0.5058
Persistent thinness	Calcium	4	-0.5765	0.9372	-0.0015	0.0024	0.3396	0.0022	0.5385
Persistent thinness	Calcium	5	-0.5098	0.9324	-0.0013	0.0024	0.3392	0.0021	0.5845
Persistent thinness	Carbohydrate	0	0.2671	0.2162	0.0030	0.0025	0.4091	0.0019	0.2168
Persistent thinness	Carbohydrate	1	0.2291	0.2159	0.0026	0.0025	0.4099	0.0019	0.2887
Persistent thinness	Carbohydrate	2	0.2327	0.2158	0.0027	0.0025	0.4098	0.0019	0.2808
Persistent thinness	Carbohydrate	3	0.2341	0.2149	0.0027	0.0025	0.4095	0.0019	0.2761
Persistent thinness	Carbohydrate	4	0.2618	0.2136	0.0030	0.0024	0.4096	0.0019	0.2203
Persistent thinness	Carbohydrate	5	0.2725	0.2123	0.0031	0.0024	0.4091	0.0019	0.1994
Persistent thinness	Carotene	0	-1.8563	6.6950	-0.0006	0.0023	0.2547	0.0026	0.7816
Persistent thinness	Carotene	1	-0.9941	6.6168	-0.0003	0.0023	0.2604	0.0026	0.8806

Persistent thinness	Carotene	2	-0.8488	6.6082	-0.0003	0.0023	0.2604	0.0026	0.8978
Persistent thinness	Carotene	3	-0.5459	6.5575	-0.0002	0.0023	0.2573	0.0026	0.9337
Persistent thinness	Carotene	4	-1.0602	6.6097	-0.0004	0.0023	0.2604	0.0026	0.8726
Persistent thinness	Carotene	5	-0.3965	6.5438	-0.0001	0.0023	0.2574	0.0026	0.9517
Persistent thinness	Fibre	0	0.0018	0.0178	0.0003	0.0025	0.4361	0.0016	0.9207
Persistent thinness	Fibre	1	0.0035	0.0176	0.0005	0.0025	0.4391	0.0016	0.8426
Persistent thinness	Fibre	2	0.0040	0.0175	0.0006	0.0025	0.4388	0.0016	0.8209
Persistent thinness	Fibre	3	0.0045	0.0174	0.0006	0.0025	0.4362	0.0016	0.7982
Persistent thinness	Fibre	4	0.0048	0.0175	0.0007	0.0025	0.4393	0.0016	0.7845
Persistent thinness	Fibre	5	0.0065	0.0172	0.0009	0.0025	0.4364	0.0016	0.7064
Persistent thinness	Fat	0	0.1627	0.0765	0.0051	0.0024	0.3347	0.0020	0.0334
Persistent thinness	Fat	1	0.1419	0.0759	0.0045	0.0024	0.3411	0.0020	0.0613
Persistent thinness	Fat	2	0.1399	0.0758	0.0044	0.0024	0.3411	0.0020	0.0649
Persistent thinness	Fat	3	0.1401	0.0757	0.0044	0.0024	0.3411	0.0020	0.0640
Persistent thinness	Fat	4	0.1417	0.0758	0.0044	0.0024	0.3410	0.0020	0.0615
Persistent thinness	Fat	5	0.1392	0.0756	0.0044	0.0024	0.3411	0.0020	0.0654
Persistent thinness	Folate	0	0.6066	0.2959	0.0051	0.0025	0.4061	0.0017	0.0404
Persistent thinness	Folate	1	0.6133	0.2943	0.0052	0.0025	0.4069	0.0017	0.0372
Persistent thinness	Folate	2	0.6240	0.2936	0.0053	0.0025	0.4068	0.0017	0.0335
Persistent thinness	Folate	3	0.6275	0.2918	0.0053	0.0025	0.4051	0.0017	0.0315
Persistent thinness	Folate	4	0.6043	0.2938	0.0051	0.0025	0.4069	0.0017	0.0397
Persistent thinness	Folate	5	0.6335	0.2908	0.0053	0.0025	0.4050	0.0017	0.0294
Persistent thinness	Food weight	0	-8.6122	2.1229	-0.0107	0.0026	0.5365	0.0015	0.0000
Persistent thinness	Food weight	1	-8.4314	2.1207	-0.0104	0.0026	0.5359	0.0015	0.0001
Persistent thinness	Food weight	2	-8.3910	2.1174	-0.0104	0.0026	0.5359	0.0015	0.0001
Persistent thinness	Food weight	3	-8.2609	2.0925	-0.0102	0.0026	0.5341	0.0015	0.0001
Persistent thinness	Food weight	4	-8.8021	2.0975	-0.0109	0.0026	0.5357	0.0015	0.0000
Persistent thinness	Food weight	5	-8.5674	2.0674	-0.0106	0.0026	0.5339	0.0015	0.0000
Persistent thinness	Iron	0	0.0157	0.0114	0.0034	0.0024	0.3692	0.0017	0.1689
Persistent thinness	Iron	1	0.0152	0.0114	0.0032	0.0024	0.3691	0.0017	0.1837
Persistent thinness	Iron	2	0.0152	0.0113	0.0032	0.0024	0.3691	0.0017	0.1789
Persistent thinness	Iron	3	0.0154	0.0113	0.0033	0.0024	0.3684	0.0017	0.1736
Persistent thinness	Iron	4	0.0122	0.0113	0.0026	0.0024	0.3691	0.0017	0.2787
Persistent thinness	Iron	5	0.0129	0.0111	0.0028	0.0024	0.3685	0.0017	0.2478
Persistent thinness	Protein	0	0.0582	0.0636	0.0022	0.0024	0.2924	0.0023	0.3607
Persistent thinness	Protein	1	0.0473	0.0635	0.0018	0.0024	0.2917	0.0023	0.4565
Persistent thinness	Protein	2	0.0497	0.0635	0.0018	0.0024	0.2918	0.0023	0.4337
Persistent thinness	Protein	3	0.0478	0.0633	0.0018	0.0023	0.2918	0.0023	0.4500

Persistent thinness	Protein	4	0.0462	0.0634	0.0017	0.0024	0.2917	0.0023	0.4662
Persistent thinness	Protein	5	0.0495	0.0632	0.0018	0.0023	0.2919	0.0023	0.4340
Persistent thinness	Vitamin B12	0	0.0053	0.0106	0.0011	0.0022	0.1574	0.0030	0.6135
Persistent thinness	Vitamin B12	1	0.0037	0.0105	0.0008	0.0022	0.1566	0.0030	0.7232
Persistent thinness	Vitamin B12	2	0.0039	0.0105	0.0008	0.0022	0.1570	0.0030	0.7100
Persistent thinness	Vitamin B12	3	0.0039	0.0105	0.0008	0.0022	0.1568	0.0030	0.7114
Persistent thinness	Vitamin B12	4	0.0028	0.0105	0.0006	0.0022	0.1567	0.0030	0.7874
Persistent thinness	Vitamin B12	5	0.0033	0.0105	0.0007	0.0022	0.1572	0.0030	0.7497
Persistent thinness	Vitamin B6	0	0.0006	0.0019	0.0008	0.0024	0.3391	0.0018	0.7438
Persistent thinness	Vitamin B6	1	0.0005	0.0019	0.0006	0.0024	0.3402	0.0018	0.8032
Persistent thinness	Vitamin B6	2	0.0006	0.0019	0.0008	0.0024	0.3402	0.0018	0.7366
Persistent thinness	Vitamin B6	3	0.0006	0.0019	0.0007	0.0024	0.3396	0.0018	0.7691
Persistent thinness	Vitamin B6	4	0.0004	0.0019	0.0005	0.0024	0.3402	0.0018	0.8308
Persistent thinness	Vitamin B6	5	0.0006	0.0019	0.0008	0.0024	0.3397	0.0018	0.7372
Persistent thinness	Vitamin C	0	-0.4657	0.2766	-0.0042	0.0025	0.3963	0.0024	0.0923
Persistent thinness	Vitamin C	1	-0.4541	0.2753	-0.0041	0.0025	0.3996	0.0024	0.0991
Persistent thinness	Vitamin C	2	-0.4457	0.2733	-0.0040	0.0025	0.3995	0.0024	0.1029
Persistent thinness	Vitamin C	3	-0.4320	0.2729	-0.0039	0.0025	0.3971	0.0024	0.1135
Persistent thinness	Vitamin C	4	-0.4667	0.2744	-0.0042	0.0025	0.3996	0.0023	0.0890
Persistent thinness	Vitamin C	5	-0.4286	0.2704	-0.0039	0.0024	0.3972	0.0024	0.1130
Persistent thinness	Vitamin D	0	0.0075	0.0070	0.0023	0.0022	0.1266	0.0033	0.2864
Persistent thinness	Vitamin D	1	0.0071	0.0070	0.0022	0.0022	0.1268	0.0033	0.3123
Persistent thinness	Vitamin D	2	0.0072	0.0070	0.0022	0.0022	0.1270	0.0033	0.3075
Persistent thinness	Vitamin D	3	0.0072	0.0070	0.0023	0.0022	0.1269	0.0033	0.3035
Persistent thinness	Vitamin D	4	0.0067	0.0070	0.0021	0.0022	0.1268	0.0033	0.3416
Persistent thinness	Vitamin D	5	0.0070	0.0070	0.0022	0.0022	0.1271	0.0033	0.3213
Persistent thinness	Vitamin E	0	0.0076	0.0114	0.0016	0.0024	0.2960	0.0021	0.5036
Persistent thinness	Vitamin E	1	0.0083	0.0114	0.0017	0.0024	0.2955	0.0021	0.4675
Persistent thinness	Vitamin E	2	0.0080	0.0113	0.0017	0.0024	0.2956	0.0021	0.4810
Persistent thinness	Vitamin E	3	0.0084	0.0113	0.0018	0.0024	0.2947	0.0021	0.4593
Persistent thinness	Vitamin E	4	0.0081	0.0114	0.0017	0.0024	0.2956	0.0021	0.4744
Persistent thinness	Vitamin E	5	0.0084	0.0112	0.0018	0.0024	0.2951	0.0021	0.4576
Schizophrenia	Alcohol	0	0.0559	0.0504	0.0025	0.0022	0.4792	0.0020	0.2672
Schizophrenia	Alcohol	1	0.0466	0.0497	0.0020	0.0022	0.4965	0.0019	0.3487
Schizophrenia	Alcohol	2	0.0338	0.0497	0.0015	0.0022	0.4966	0.0019	0.4956
Schizophrenia	Alcohol	3	0.0498	0.0497	0.0022	0.0022	0.4973	0.0019	0.3160
Schizophrenia	Alcohol	4	0.0091	0.0409	0.0004	0.0018	0.4962	0.0020	0.8244
Schizophrenia	Alcohol	5	0.0141	0.0409	0.0006	0.0018	0.4971	0.0020	0.7293

Schizophrenia	Calcium	0	5.6831	0.8311	0.0147	0.0021	0.3396	0.0022	0.0000
Schizophrenia	Calcium	1	5.7534	0.8306	0.0149	0.0021	0.3397	0.0022	0.0000
Schizophrenia	Calcium	2	5.7551	0.8300	0.0149	0.0021	0.3395	0.0022	0.0000
Schizophrenia	Calcium	3	5.6744	0.8275	0.0147	0.0021	0.3393	0.0021	0.0000
Schizophrenia	Calcium	4	5.9462	0.8267	0.0154	0.0021	0.3397	0.0022	0.0000
Schizophrenia	Calcium	5	5.7678	0.8228	0.0149	0.0021	0.3392	0.0021	0.0000
Schizophrenia	Carbohydrate	0	0.9689	0.1907	0.0111	0.0022	0.4092	0.0019	0.0000
Schizophrenia	Carbohydrate	1	1.0014	0.1904	0.0114	0.0022	0.4099	0.0019	0.0000
Schizophrenia	Carbohydrate	2	0.9870	0.1904	0.0113	0.0022	0.4098	0.0019	0.0000
Schizophrenia	Carbohydrate	3	0.9826	0.1895	0.0112	0.0022	0.4095	0.0019	0.0000
Schizophrenia	Carbohydrate	4	1.0445	0.1884	0.0119	0.0021	0.4096	0.0019	0.0000
Schizophrenia	Carbohydrate	5	0.9867	0.1874	0.0113	0.0021	0.4091	0.0019	0.0000
Schizophrenia	Carotene	0	38.6177	5.9050	0.0135	0.0021	0.2547	0.0026	0.0000
Schizophrenia	Carotene	1	38.5790	5.8360	0.0135	0.0020	0.2604	0.0026	0.0000
Schizophrenia	Carotene	2	37.5222	5.8310	0.0131	0.0020	0.2604	0.0026	0.0000
Schizophrenia	Carotene	3	37.9546	5.7840	0.0133	0.0020	0.2573	0.0026	0.0000
Schizophrenia	Carotene	4	39.7246	5.8305	0.0139	0.0020	0.2604	0.0026	0.0000
Schizophrenia	Carotene	5	37.5200	5.7753	0.0131	0.0020	0.2574	0.0026	0.0000
Schizophrenia	Fibre	0	0.1556	0.0157	0.0222	0.0022	0.4361	0.0016	0.0000
Schizophrenia	Fibre	1	0.1565	0.0155	0.0223	0.0022	0.4391	0.0016	0.0000
Schizophrenia	Fibre	2	0.1534	0.0155	0.0219	0.0022	0.4388	0.0016	0.0000
Schizophrenia	Fibre	3	0.1545	0.0153	0.0220	0.0022	0.4362	0.0016	0.0000
Schizophrenia	Fibre	4	0.1637	0.0154	0.0233	0.0022	0.4393	0.0016	0.0000
Schizophrenia	Fibre	5	0.1560	0.0152	0.0222	0.0022	0.4364	0.0016	0.0000
Schizophrenia	Fat	0	0.3755	0.0675	0.0118	0.0021	0.3347	0.0020	0.0000
Schizophrenia	Fat	1	0.3863	0.0669	0.0121	0.0021	0.3411	0.0020	0.0000
Schizophrenia	Fat	2	0.3741	0.0669	0.0117	0.0021	0.3411	0.0020	0.0000
Schizophrenia	Fat	3	0.3865	0.0667	0.0121	0.0021	0.3411	0.0020	0.0000
Schizophrenia	Fat	4	0.3782	0.0669	0.0119	0.0021	0.3410	0.0020	0.0000
Schizophrenia	Fat	5	0.3650	0.0667	0.0115	0.0021	0.3411	0.0020	0.0000
Schizophrenia	Folate	0	1.8569	0.2609	0.0157	0.0022	0.4061	0.0017	0.0000
Schizophrenia	Folate	1	1.8747	0.2595	0.0158	0.0022	0.4069	0.0017	0.0000
Schizophrenia	Folate	2	1.8614	0.2590	0.0157	0.0022	0.4068	0.0017	0.0000
Schizophrenia	Folate	3	1.8498	0.2574	0.0156	0.0022	0.4051	0.0017	0.0000
Schizophrenia	Folate	4	1.9448	0.2591	0.0164	0.0022	0.4069	0.0017	0.0000
Schizophrenia	Folate	5	1.8758	0.2566	0.0158	0.0022	0.4050	0.0017	0.0000
Schizophrenia	Food weight	0	14.3061	1.8721	0.0177	0.0023	0.5365	0.0015	0.0000
Schizophrenia	Food weight	1	14.1833	1.8701	0.0175	0.0023	0.5359	0.0015	0.0000

Schizophrenia	Food weight	2	13.9172	1.8681	0.0172	0.0023	0.5359	0.0015	0.0000
Schizophrenia	Food weight	3	13.9261	1.8454	0.0172	0.0023	0.5341	0.0015	0.0000
Schizophrenia	Food weight	4	13.8751	1.8500	0.0172	0.0023	0.5357	0.0015	0.0000
Schizophrenia	Food weight	5	13.3168	1.8243	0.0165	0.0023	0.5339	0.0015	0.0000
Schizophrenia	Iron	0	0.0677	0.0101	0.0145	0.0022	0.3691	0.0017	0.0000
Schizophrenia	Iron	1	0.0682	0.0101	0.0146	0.0021	0.3691	0.0017	0.0000
Schizophrenia	Iron	2	0.0643	0.0100	0.0138	0.0021	0.3691	0.0017	0.0000
Schizophrenia	Iron	3	0.0679	0.0100	0.0145	0.0021	0.3684	0.0017	0.0000
Schizophrenia	Iron	4	0.0707	0.0099	0.0151	0.0021	0.3691	0.0017	0.0000
Schizophrenia	Iron	5	0.0656	0.0098	0.0140	0.0021	0.3685	0.0017	0.0000
Schizophrenia	Protein	0	0.3065	0.0561	0.0114	0.0021	0.2924	0.0023	0.0000
Schizophrenia	Protein	1	0.3150	0.0560	0.0117	0.0021	0.2917	0.0023	0.0000
Schizophrenia	Protein	2	0.3303	0.0560	0.0122	0.0021	0.2918	0.0023	0.0000
Schizophrenia	Protein	3	0.3124	0.0559	0.0116	0.0021	0.2918	0.0023	0.0000
Schizophrenia	Protein	4	0.3232	0.0560	0.0120	0.0021	0.2916	0.0023	0.0000
Schizophrenia	Protein	5	0.3318	0.0558	0.0123	0.0021	0.2919	0.0023	0.0000
Schizophrenia	Vitamin B12	0	0.0432	0.0093	0.0091	0.0020	0.1574	0.0030	0.0000
Schizophrenia	Vitamin B12	1	0.0446	0.0093	0.0094	0.0020	0.1566	0.0030	0.0000
Schizophrenia	Vitamin B12	2	0.0447	0.0093	0.0094	0.0020	0.1570	0.0030	0.0000
Schizophrenia	Vitamin B12	3	0.0443	0.0093	0.0094	0.0020	0.1568	0.0030	0.0000
Schizophrenia	Vitamin B12	4	0.0462	0.0093	0.0098	0.0020	0.1567	0.0030	0.0000
Schizophrenia	Vitamin B12	5	0.0454	0.0093	0.0096	0.0020	0.1572	0.0030	0.0000
Schizophrenia	Vitamin B6	0	0.0042	0.0017	0.0054	0.0021	0.3391	0.0018	0.0117
Schizophrenia	Vitamin B6	1	0.0045	0.0017	0.0057	0.0021	0.3402	0.0018	0.0074
Schizophrenia	Vitamin B6	2	0.0057	0.0017	0.0073	0.0021	0.3402	0.0018	0.0005
Schizophrenia	Vitamin B6	3	0.0043	0.0017	0.0055	0.0021	0.3396	0.0018	0.0092
Schizophrenia	Vitamin B6	4	0.0049	0.0017	0.0063	0.0021	0.3402	0.0018	0.0032
Schizophrenia	Vitamin B6	5	0.0059	0.0017	0.0075	0.0021	0.3397	0.0018	0.0004
Schizophrenia	Vitamin C	0	0.5161	0.2440	0.0047	0.0022	0.3963	0.0024	0.0344
Schizophrenia	Vitamin C	1	0.5304	0.2428	0.0048	0.0022	0.3996	0.0024	0.0289
Schizophrenia	Vitamin C	2	0.4494	0.2411	0.0041	0.0022	0.3995	0.0024	0.0624
Schizophrenia	Vitamin C	3	0.5050	0.2408	0.0046	0.0022	0.3971	0.0024	0.0360
Schizophrenia	Vitamin C	4	0.6185	0.2421	0.0056	0.0022	0.3996	0.0023	0.0106
Schizophrenia	Vitamin C	5	0.4745	0.2387	0.0043	0.0022	0.3972	0.0024	0.0468
Schizophrenia	Vitamin D	0	0.0272	0.0062	0.0085	0.0019	0.1266	0.0033	0.0000
Schizophrenia	Vitamin D	1	0.0277	0.0062	0.0086	0.0019	0.1268	0.0033	0.0000
Schizophrenia	Vitamin D	2	0.0274	0.0062	0.0085	0.0019	0.1270	0.0033	0.0000
Schizophrenia	Vitamin D	3	0.0275	0.0062	0.0085	0.0019	0.1269	0.0033	0.0000

Schizophrenia	Vitamin D	4	0.0287	0.0062	0.0089	0.0019	0.1268	0.0033	0.0000
Schizophrenia	Vitamin D	5	0.0279	0.0062	0.0087	0.0019	0.1271	0.0033	0.0000
Schizophrenia	Vitamin E	0	0.0614	0.0101	0.0129	0.0021	0.2960	0.0021	0.0000
Schizophrenia	Vitamin E	1	0.0612	0.0101	0.0128	0.0021	0.2955	0.0021	0.0000
Schizophrenia	Vitamin E	2	0.0561	0.0100	0.0118	0.0021	0.2956	0.0021	0.0000
Schizophrenia	Vitamin E	3	0.0605	0.0100	0.0127	0.0021	0.2947	0.0021	0.0000
Schizophrenia	Vitamin E	4	0.0641	0.0100	0.0135	0.0021	0.2956	0.0021	0.0000
Schizophrenia	Vitamin E	5	0.0569	0.0099	0.0119	0.0021	0.2952	0.0021	0.0000
