# Supplementary material for: Association between polygenic propensity for psychiatric disorders and nutrient intake
Source: Commun Biol. 2021 Aug 26;4:965. doi: 10.1038/s42003-021-02469-4 (PMC8390493; doi:10.1038/s42003-021-02469-4)
Supplement: Supplementary file 5 — Supplementary Data 2 [file 42003_2021_2469_MOESM5_ESM.pdf]

PRS	Nutrient Intake	Model	Estimate	SE	Standardised Es	Standardised SI	R-squared	R-squared SE	P-value
ADHD	Alcohol	0	0.114	0.048	0.005	0.002	0.479	0.002	0.018
ADHD	Alcohol	1	0.152	0.048	0.007	0.002	0.496	0.002	0.001
ADHD	Alcohol	2	0.207	0.048	0.009	0.002	0.497	0.002	0.000
ADHD	Alcohol	3	0.229	0.039	0.010	0.002	0.496	0.002	0.000
ADHD	Alcohol	4	0.227	0.039	0.010	0.002	0.497	0.002	0.000
ADHD	Calcium	0	-3.449	0.797	-0.009	0.002	0.340	0.002	0.000
ADHD	Calcium	1	-3.351	0.797	-0.009	0.002	0.340	0.002	0.000
ADHD	Calcium	2	-2.305	0.799	-0.006	0.002	0.340	0.002	0.004
ADHD	Calcium	3	-2.125	0.795	-0.006	0.002	0.340	0.002	0.008
ADHD	Calcium	4	-2.255	0.792	-0.006	0.002	0.339	0.002	0.004
ADHD	Carbohydrate	0	-1.358	0.183	-0.016	0.002	0.409	0.002	0.000
ADHD	Carbohydrate	1	-1.253	0.183	-0.014	0.002	0.410	0.002	0.000
ADHD	Carbohydrate	2	-1.079	0.183	-0.012	0.002	0.410	0.002	0.000
ADHD	Carbohydrate	3	-1.068	0.181	-0.012	0.002	0.409	0.002	0.000
ADHD	Carbohydrate	4	-1.110	0.180	-0.013	0.002	0.409	0.002	0.000
ADHD	Carotene	0	5.434	5.665	0.002	0.002	0.255	0.003	0.338
ADHD	Carotene	1	-0.586	5.601	0.000	0.002	0.260	0.003	0.917
ADHD	Carotene	2	8.201	5.612	0.003	0.002	0.260	0.003	0.144
ADHD	Carotene	3	10.854	5.610	0.004	0.002	0.260	0.003	0.053
ADHD	Carotene	4	9.303	5.561	0.003	0.002	0.257	0.003	0.094
ADHD	Fibre	0	-0.088	0.015	-0.013	0.002	0.436	0.002	0.000
ADHD	Fibre	1	-0.102	0.015	-0.015	0.002	0.439	0.002	0.000
ADHD	Fibre	2	-0.065	0.015	-0.009	0.002	0.439	0.002	0.000
ADHD	Fibre	3	-0.055	0.015	-0.008	0.002	0.439	0.002	0.000
ADHD	Fibre	4	-0.058	0.015	-0.008	0.002	0.436	0.002	0.000
ADHD	Fat	0	-0.287	0.065	-0.009	0.002	0.335	0.002	0.000
ADHD	Fat	1	-0.198	0.064	-0.006	0.002	0.341	0.002	0.002
ADHD	Fat	2	-0.145	0.064	-0.005	0.002	0.341	0.002	0.024
ADHD	Fat	3	-0.169	0.064	-0.005	0.002	0.341	0.002	0.009
ADHD	Fat	4	-0.170	0.064	-0.005	0.002	0.341	0.002	0.008
ADHD	Folate	0	-1.478	0.250	-0.013	0.002	0.406	0.002	0.000
ADHD	Folate	1	-1.594	0.249	-0.013	0.002	0.407	0.002	0.000
ADHD	Folate	2	-1.078	0.249	-0.009	0.002	0.407	0.002	0.000
ADHD	Folate	3	-0.934	0.249	-0.008	0.002	0.407	0.002	0.000
ADHD	Folate	4	-0.994	0.247	-0.008	0.002	0.405	0.002	0.000
ADHD	Food weight	0	4.827	1.796	0.006	0.002	0.537	0.001	0.007
ADHD	Food weight	1	4.224	1.795	0.005	0.002	0.536	0.001	0.019
ADHD	Food weight	2	7.330	1.798	0.009	0.002	0.536	0.001	0.000
ADHD	Food weight	3	7.600	1.781	0.009	0.002	0.536	0.001	0.000

ADHD	Food weight	4	6.896	1.757	0.009	0.002	0.534	0.001	0.000
ADHD	Iron	0	-0.094	0.010	-0.020	0.002	0.369	0.002	0.000
ADHD	Iron	1	-0.093	0.010	-0.020	0.002	0.369	0.002	0.000
ADHD	Iron	2	-0.054	0.010	-0.012	0.002	0.369	0.002	0.000
ADHD	Iron	3	-0.044	0.010	-0.010	0.002	0.369	0.002	0.000
ADHD	Iron	4	-0.046	0.010	-0.010	0.002	0.369	0.002	0.000
ADHD	Protein	0	0.018	0.054	0.001	0.002	0.292	0.002	0.742
ADHD	Protein	1	0.052	0.054	0.002	0.002	0.292	0.002	0.333
ADHD	Protein	2	0.082	0.054	0.003	0.002	0.292	0.002	0.126
ADHD	Protein	3	0.093	0.054	0.003	0.002	0.292	0.002	0.086
ADHD	Protein	4	0.090	0.054	0.003	0.002	0.292	0.002	0.094
ADHD	Vitamin B12	0	-0.032	0.009	-0.007	0.002	0.157	0.003	0.000
ADHD	Vitamin B12	1	-0.028	0.009	-0.006	0.002	0.157	0.003	0.002
ADHD	Vitamin B12	2	-0.013	0.009	-0.003	0.002	0.157	0.003	0.141
ADHD	Vitamin B12	3	-0.010	0.009	-0.002	0.002	0.157	0.003	0.270
ADHD	Vitamin B12	4	-0.010	0.009	-0.002	0.002	0.157	0.003	0.266
ADHD	Vitamin B6	0	-0.001	0.002	-0.002	0.002	0.339	0.002	0.438
ADHD	Vitamin B6	1	-0.001	0.002	-0.002	0.002	0.340	0.002	0.392
ADHD	Vitamin B6	2	-0.002	0.002	-0.002	0.002	0.340	0.002	0.282
ADHD	Vitamin B6	3	-0.001	0.002	-0.001	0.002	0.340	0.002	0.580
ADHD	Vitamin B6	4	-0.001	0.002	-0.002	0.002	0.340	0.002	0.460
ADHD	Vitamin C	0	-1.259	0.234	-0.011	0.002	0.396	0.002	0.000
ADHD	Vitamin C	1	-1.389	0.233	-0.013	0.002	0.400	0.002	0.000
ADHD	Vitamin C	2	-0.501	0.232	-0.005	0.002	0.399	0.002	0.031
ADHD	Vitamin C	3	-0.317	0.232	-0.003	0.002	0.399	0.002	0.172
ADHD	Vitamin C	4	-0.378	0.230	-0.003	0.002	0.397	0.002	0.100
ADHD	Vitamin D	0	-0.024	0.006	-0.007	0.002	0.127	0.003	0.000
ADHD	Vitamin D	1	-0.023	0.006	-0.007	0.002	0.127	0.003	0.000
ADHD	Vitamin D	2	-0.015	0.006	-0.005	0.002	0.127	0.003	0.012
ADHD	Vitamin D	3	-0.013	0.006	-0.004	0.002	0.127	0.003	0.030
ADHD	Vitamin D	4	-0.013	0.006	-0.004	0.002	0.127	0.003	0.027
ADHD	Vitamin E	0	-0.085	0.010	-0.018	0.002	0.296	0.002	0.000
ADHD	Vitamin E	1	-0.089	0.010	-0.019	0.002	0.296	0.002	0.000
ADHD	Vitamin E	2	-0.053	0.010	-0.011	0.002	0.296	0.002	0.000
ADHD	Vitamin E	3	-0.048	0.010	-0.010	0.002	0.296	0.002	0.000
ADHD	Vitamin E	4	-0.050	0.010	-0.010	0.002	0.295	0.002	0.000
Alcohol depende	Alcohol	0	0.394	0.051	0.017	0.002	0.479	0.002	0.000
Alcohol depende	Alcohol	1	0.402	0.050	0.018	0.002	0.496	0.002	0.000
Alcohol depende	Alcohol	2	0.421	0.050	0.019	0.002	0.497	0.002	0.000
Alcohol depende	Alcohol	3	0.229	0.041	0.010	0.002	0.496	0.002	0.000
Alcohol depende	Alcohol	4	0.227	0.041	0.010	0.002	0.497	0.002	0.000

Alcohol depende Calcium	0	-3.416	0.842	-0.009	0.002	0.340	0.002	0.000
Alcohol depende Calcium	1	-3.413	0.841	-0.009	0.002	0.340	0.002	0.000
Alcohol depende Calcium	2	-3.028	0.840	-0.008	0.002	0.340	0.002	0.000
Alcohol depende Calcium	3	-2.289	0.836	-0.006	0.002	0.340	0.002	0.006
Alcohol depende Calcium	4	-2.300	0.833	-0.006	0.002	0.339	0.002	0.006
Alcohol depende Carbohydrate	0	-1.180	0.193	-0.014	0.002	0.409	0.002	0.000
Alcohol depende Carbohydrate	1	-1.169	0.193	-0.013	0.002	0.410	0.002	0.000
Alcohol depende Carbohydrate	2	-1.108	0.193	-0.013	0.002	0.410	0.002	0.000
Alcohol depende Carbohydrate	3	-0.885	0.191	-0.010	0.002	0.409	0.002	0.000
Alcohol depende Carbohydrate	4	-0.893	0.190	-0.010	0.002	0.409	0.002	0.000
Alcohol depende Carotene	0	-0.431	5.980	0.000	0.002	0.255	0.003	0.943
Alcohol depende Carotene	1	-1.410	5.910	-0.001	0.002	0.260	0.003	0.811
Alcohol depende Carotene	2	1.601	5.904	0.001	0.002	0.260	0.003	0.786
Alcohol depende Carotene	3	3.726	5.901	0.001	0.002	0.260	0.003	0.528
Alcohol depende Carotene	4	3.514	5.849	0.001	0.002	0.257	0.003	0.548
Alcohol depende Fibre	0	-0.049	0.016	-0.007	0.002	0.436	0.002	0.002
Alcohol depende Fibre	1	-0.051	0.016	-0.007	0.002	0.439	0.002	0.001
Alcohol depende Fibre	2	-0.038	0.016	-0.006	0.002	0.439	0.002	0.014
Alcohol depende Fibre	3	-0.019	0.016	-0.003	0.002	0.439	0.002	0.212
Alcohol depende Fibre	4	-0.020	0.015	-0.003	0.002	0.436	0.002	0.198
Alcohol depende Fat	0	-0.126	0.068	-0.004	0.002	0.335	0.002	0.065
Alcohol depende Fat	1	-0.113	0.068	-0.004	0.002	0.341	0.002	0.095
Alcohol depende Fat	2	-0.096	0.068	-0.003	0.002	0.341	0.002	0.158
Alcohol depende Fat	3	-0.095	0.068	-0.003	0.002	0.341	0.002	0.159
Alcohol depende Fat	4	-0.093	0.068	-0.003	0.002	0.341	0.002	0.167
Alcohol depende Folate	0	-0.642	0.264	-0.005	0.002	0.406	0.002	0.015
Alcohol depende Folate	1	-0.664	0.263	-0.006	0.002	0.407	0.002	0.012
Alcohol depende Folate	2	-0.472	0.262	-0.004	0.002	0.407	0.002	0.072
Alcohol depende Folate	3	-0.352	0.262	-0.003	0.002	0.407	0.002	0.179
Alcohol depende Folate	4	-0.359	0.260	-0.003	0.002	0.405	0.002	0.168
Alcohol depende Food weight	0	5.897	1.896	0.007	0.002	0.537	0.001	0.002
Alcohol depende Food weight	1	5.822	1.894	0.007	0.002	0.536	0.001	0.002
Alcohol depende Food weight	2	6.948	1.892	0.009	0.002	0.536	0.001	0.000
Alcohol depende Food weight	3	5.241	1.874	0.007	0.002	0.536	0.001	0.005
Alcohol depende Food weight	4	5.078	1.848	0.006	0.002	0.534	0.001	0.006
Alcohol depende Iron	0	-0.035	0.010	-0.007	0.002	0.369	0.002	0.001
Alcohol depende Iron	1	-0.034	0.010	-0.007	0.002	0.369	0.002	0.001
Alcohol depende Iron	2	-0.021	0.010	-0.004	0.002	0.369	0.002	0.042
Alcohol depende Iron	3	-0.022	0.010	-0.005	0.002	0.369	0.002	0.030
Alcohol depende Iron	4	-0.022	0.010	-0.005	0.002	0.369	0.002	0.029
Alcohol depende Protein	0	-0.091	0.057	-0.003	0.002	0.292	0.002	0.111

Alcohol depende	Protein	1	-0.086	0.057	-0.003	0.002	0.292	0.002	0.128
Alcohol depende	Protein	2	-0.072	0.057	-0.003	0.002	0.292	0.002	0.205
Alcohol depende	Protein	3	-0.058	0.057	-0.002	0.002	0.292	0.002	0.307
Alcohol depende	Protein	4	-0.057	0.057	-0.002	0.002	0.292	0.002	0.317
Alcohol depende	Vitamin B12	0	-0.007	0.009	-0.001	0.002	0.157	0.003	0.469
Alcohol depende	Vitamin B12	1	-0.006	0.009	-0.001	0.002	0.157	0.003	0.500
Alcohol depende	Vitamin B12	2	-0.001	0.009	0.000	0.002	0.157	0.003	0.932
Alcohol depende	Vitamin B12	3	-0.001	0.009	0.000	0.002	0.157	0.003	0.934
Alcohol depende	Vitamin B12	4	-0.001	0.009	0.000	0.002	0.157	0.003	0.942
Alcohol depende	Vitamin B6	0	-0.002	0.002	-0.002	0.002	0.339	0.002	0.315
Alcohol depende	Vitamin B6	1	-0.002	0.002	-0.002	0.002	0.340	0.002	0.295
Alcohol depende	Vitamin B6	2	-0.002	0.002	-0.002	0.002	0.340	0.002	0.335
Alcohol depende	Vitamin B6	3	-0.001	0.002	-0.001	0.002	0.340	0.002	0.560
Alcohol depende	Vitamin B6	4	-0.001	0.002	-0.001	0.002	0.340	0.002	0.544
Alcohol depende	Vitamin C	0	-0.551	0.247	-0.005	0.002	0.396	0.002	0.026
Alcohol depende	Vitamin C	1	-0.575	0.246	-0.005	0.002	0.400	0.002	0.019
Alcohol depende	Vitamin C	2	-0.255	0.244	-0.002	0.002	0.399	0.002	0.295
Alcohol depende	Vitamin C	3	-0.146	0.244	-0.001	0.002	0.399	0.002	0.550
Alcohol depende	Vitamin C	4	-0.158	0.242	-0.001	0.002	0.397	0.002	0.515
Alcohol depende	Vitamin D	0	-0.002	0.006	-0.001	0.002	0.127	0.003	0.703
Alcohol depende	Vitamin D	1	-0.002	0.006	-0.001	0.002	0.127	0.003	0.703
Alcohol depende	Vitamin D	2	0.001	0.006	0.000	0.002	0.127	0.003	0.924
Alcohol depende	Vitamin D	3	0.001	0.006	0.000	0.002	0.127	0.003	0.842
Alcohol depende	Vitamin D	4	0.001	0.006	0.000	0.002	0.127	0.003	0.847
Alcohol depende	Vitamin E	0	-0.026	0.010	-0.006	0.002	0.296	0.002	0.010
Alcohol depende	Vitamin E	1	-0.027	0.010	-0.006	0.002	0.296	0.002	0.008
Alcohol depende	Vitamin E	2	-0.015	0.010	-0.003	0.002	0.296	0.002	0.148
Alcohol depende	Vitamin E	3	-0.008	0.010	-0.002	0.002	0.296	0.002	0.452
Alcohol depende	Vitamin E	4	-0.008	0.010	-0.002	0.002	0.295	0.002	0.457
Anorexia Nervosa	Alcohol	0	-0.035	0.048	-0.002	0.002	0.479	0.002	0.475
Anorexia Nervosa	Alcohol	1	-0.025	0.048	-0.001	0.002	0.496	0.002	0.605
Anorexia Nervosa	Alcohol	2	-0.040	0.048	-0.002	0.002	0.497	0.002	0.404
Anorexia Nervosa	Alcohol	3	-0.073	0.039	-0.003	0.002	0.496	0.002	0.065
Anorexia Nervosa	Alcohol	4	-0.071	0.039	-0.003	0.002	0.497	0.002	0.071
Anorexia Nervosa	Calcium	0	0.976	0.800	0.003	0.002	0.340	0.002	0.222
Anorexia Nervosa	Calcium	1	0.960	0.799	0.003	0.002	0.340	0.002	0.230
Anorexia Nervosa	Calcium	2	0.730	0.799	0.002	0.002	0.340	0.002	0.361
Anorexia Nervosa	Calcium	3	0.797	0.794	0.002	0.002	0.340	0.002	0.316
Anorexia Nervosa	Calcium	4	0.626	0.792	0.002	0.002	0.339	0.002	0.429
Anorexia Nervosa	Carbohydrate	0	0.133	0.184	0.002	0.002	0.409	0.002	0.469
Anorexia Nervosa	Carbohydrate	1	0.135	0.183	0.002	0.002	0.410	0.002	0.461

Anorexia Nervosa	Carbohydrate	2	0.090	0.183	0.001	0.002	0.410	0.002	0.622
Anorexia Nervosa	Carbohydrate	3	0.112	0.181	0.001	0.002	0.409	0.002	0.536
Anorexia Nervosa	Carbohydrate	4	0.075	0.180	0.001	0.002	0.409	0.002	0.677
Anorexia Nervosa	Carotene	0	11.295	5.683	0.004	0.002	0.255	0.003	0.047
Anorexia Nervosa	Carotene	1	10.295	5.616	0.004	0.002	0.260	0.003	0.067
Anorexia Nervosa	Carotene	2	8.102	5.610	0.003	0.002	0.260	0.003	0.149
Anorexia Nervosa	Carotene	3	8.375	5.605	0.003	0.002	0.260	0.003	0.135
Anorexia Nervosa	Carotene	4	6.698	5.556	0.002	0.002	0.257	0.003	0.228
Anorexia Nervosa	Fibre	0	0.057	0.015	0.008	0.002	0.436	0.002	0.000
Anorexia Nervosa	Fibre	1	0.054	0.015	0.008	0.002	0.439	0.002	0.000
Anorexia Nervosa	Fibre	2	0.045	0.015	0.007	0.002	0.439	0.002	0.002
Anorexia Nervosa	Fibre	3	0.048	0.015	0.007	0.002	0.439	0.002	0.001
Anorexia Nervosa	Fibre	4	0.043	0.015	0.006	0.002	0.436	0.002	0.003
Anorexia Nervosa	Fat	0	-0.080	0.065	-0.003	0.002	0.335	0.002	0.216
Anorexia Nervosa	Fat	1	-0.071	0.064	-0.002	0.002	0.341	0.002	0.271
Anorexia Nervosa	Fat	2	-0.087	0.064	-0.003	0.002	0.341	0.002	0.177
Anorexia Nervosa	Fat	3	-0.090	0.064	-0.003	0.002	0.341	0.002	0.160
Anorexia Nervosa	Fat	4	-0.095	0.064	-0.003	0.002	0.341	0.002	0.140
Anorexia Nervosa	Folate	0	0.926	0.251	0.008	0.002	0.406	0.002	0.000
Anorexia Nervosa	Folate	1	0.900	0.250	0.008	0.002	0.407	0.002	0.000
Anorexia Nervosa	Folate	2	0.781	0.249	0.007	0.002	0.407	0.002	0.002
Anorexia Nervosa	Folate	3	0.798	0.249	0.007	0.002	0.407	0.002	0.001
Anorexia Nervosa	Folate	4	0.730	0.247	0.006	0.002	0.405	0.002	0.003
Anorexia Nervosa	Food weight	0	1.775	1.801	0.002	0.002	0.537	0.001	0.324
Anorexia Nervosa	Food weight	1	1.737	1.799	0.002	0.002	0.536	0.001	0.334
Anorexia Nervosa	Food weight	2	0.994	1.797	0.001	0.002	0.536	0.001	0.580
Anorexia Nervosa	Food weight	3	0.626	1.779	0.001	0.002	0.536	0.001	0.725
Anorexia Nervosa	Food weight	4	0.096	1.755	0.000	0.002	0.534	0.001	0.956
Anorexia Nervosa	Iron	0	0.035	0.010	0.007	0.002	0.369	0.002	0.000
Anorexia Nervosa	Iron	1	0.035	0.010	0.007	0.002	0.369	0.002	0.000
Anorexia Nervosa	Iron	2	0.025	0.010	0.005	0.002	0.369	0.002	0.010
Anorexia Nervosa	Iron	3	0.025	0.010	0.005	0.002	0.369	0.002	0.010
Anorexia Nervosa	Iron	4	0.023	0.010	0.005	0.002	0.369	0.002	0.017
Anorexia Nervosa	Protein	0	-0.047	0.054	-0.002	0.002	0.292	0.002	0.381
Anorexia Nervosa	Protein	1	-0.046	0.054	-0.002	0.002	0.292	0.002	0.397
Anorexia Nervosa	Protein	2	-0.048	0.054	-0.002	0.002	0.292	0.002	0.377
Anorexia Nervosa	Protein	3	-0.045	0.054	-0.002	0.002	0.292	0.002	0.401
Anorexia Nervosa	Protein	4	-0.054	0.054	-0.002	0.002	0.292	0.002	0.316
Anorexia Nervosa	Vitamin B12	0	0.009	0.009	0.002	0.002	0.157	0.003	0.300
Anorexia Nervosa	Vitamin B12	1	0.009	0.009	0.002	0.002	0.157	0.003	0.294
Anorexia Nervosa	Vitamin B12	2	0.006	0.009	0.001	0.002	0.157	0.003	0.497

Anorexia Nervos	Vitamin B12	3	0.006	0.009	0.001	0.002	0.157	0.003	0.478
Anorexia Nervos	Vitamin B12	4	0.006	0.009	0.001	0.002	0.157	0.003	0.538
Anorexia Nervos	Vitamin B6	0	-0.001	0.002	-0.001	0.002	0.339	0.002	0.650
Anorexia Nervos	Vitamin B6	1	-0.001	0.002	-0.001	0.002	0.340	0.002	0.589
Anorexia Nervos	Vitamin B6	2	0.000	0.002	-0.001	0.002	0.340	0.002	0.792
Anorexia Nervos	Vitamin B6	3	0.000	0.002	0.000	0.002	0.340	0.002	0.856
Anorexia Nervos	Vitamin B6	4	-0.001	0.002	-0.001	0.002	0.340	0.002	0.673
Anorexia Nervos	Vitamin C	0	0.924	0.235	0.008	0.002	0.396	0.002	0.000
Anorexia Nervos	Vitamin C	1	0.897	0.234	0.008	0.002	0.400	0.002	0.000
Anorexia Nervos	Vitamin C	2	0.678	0.232	0.006	0.002	0.399	0.002	0.004
Anorexia Nervos	Vitamin C	3	0.699	0.232	0.006	0.002	0.399	0.002	0.003
Anorexia Nervos	Vitamin C	4	0.640	0.230	0.006	0.002	0.397	0.002	0.005
Anorexia Nervos	Vitamin D	0	0.011	0.006	0.003	0.002	0.127	0.003	0.068
Anorexia Nervos	Vitamin D	1	0.011	0.006	0.003	0.002	0.127	0.003	0.072
Anorexia Nervos	Vitamin D	2	0.009	0.006	0.003	0.002	0.127	0.003	0.139
Anorexia Nervos	Vitamin D	3	0.009	0.006	0.003	0.002	0.127	0.003	0.129
Anorexia Nervos	Vitamin D	4	0.009	0.006	0.003	0.002	0.127	0.003	0.154
Anorexia Nervos	Vitamin E	0	0.032	0.010	0.007	0.002	0.296	0.002	0.001
Anorexia Nervos	Vitamin E	1	0.032	0.010	0.007	0.002	0.296	0.002	0.001
Anorexia Nervos	Vitamin E	2	0.023	0.010	0.005	0.002	0.296	0.002	0.019
Anorexia Nervos	Vitamin E	3	0.023	0.010	0.005	0.002	0.296	0.002	0.016
Anorexia Nervos	Vitamin E	4	0.021	0.010	0.004	0.002	0.295	0.002	0.029
Autism spectrum	Alcohol	0	-0.043	0.048	-0.002	0.002	0.479	0.002	0.371
Autism spectrum	Alcohol	1	-0.051	0.048	-0.002	0.002	0.496	0.002	0.289
Autism spectrum	Alcohol	2	-0.074	0.048	-0.003	0.002	0.497	0.002	0.122
Autism spectrum	Alcohol	3	0.025	0.039	0.001	0.002	0.496	0.002	0.531
Autism spectrum	Alcohol	4	0.024	0.039	0.001	0.002	0.497	0.002	0.535
Autism spectrum	Calcium	0	1.017	0.799	0.003	0.002	0.340	0.002	0.203
Autism spectrum	Calcium	1	1.118	0.799	0.003	0.002	0.340	0.002	0.162
Autism spectrum	Calcium	2	0.959	0.799	0.003	0.002	0.340	0.002	0.230
Autism spectrum	Calcium	3	0.662	0.795	0.002	0.002	0.340	0.002	0.405
Autism spectrum	Calcium	4	0.641	0.792	0.002	0.002	0.339	0.002	0.418
Autism spectrum	Carbohydrate	0	0.155	0.183	0.002	0.002	0.409	0.002	0.399
Autism spectrum	Carbohydrate	1	0.206	0.183	0.002	0.002	0.410	0.002	0.261
Autism spectrum	Carbohydrate	2	0.166	0.183	0.002	0.002	0.410	0.002	0.366
Autism spectrum	Carbohydrate	3	0.055	0.181	0.001	0.002	0.409	0.002	0.761
Autism spectrum	Carbohydrate	4	0.053	0.180	0.001	0.002	0.409	0.002	0.767
Autism spectrum	Carotene	0	6.905	5.681	0.002	0.002	0.255	0.003	0.224
Autism spectrum	Carotene	1	6.603	5.615	0.002	0.002	0.260	0.003	0.240
Autism spectrum	Carotene	2	4.200	5.611	0.002	0.002	0.260	0.003	0.454
Autism spectrum	Carotene	3	4.552	5.607	0.002	0.002	0.260	0.003	0.417

Autism spectrum Carotene	4	4.678	5.558	0.002	0.002	0.257	0.003	0.400
Autism spectrum Fibre	0	0.024	0.015	0.003	0.002	0.436	0.002	0.113
Autism spectrum Fibre	1	0.024	0.015	0.003	0.002	0.439	0.002	0.114
Autism spectrum Fibre	2	0.015	0.015	0.002	0.002	0.439	0.002	0.316
Autism spectrum Fibre	3	0.011	0.015	0.002	0.002	0.439	0.002	0.476
Autism spectrum Fibre	4	0.011	0.015	0.002	0.002	0.436	0.002	0.465
Autism spectrum Fat	0	0.158	0.065	0.005	0.002	0.335	0.002	0.015
Autism spectrum Fat	1	0.178	0.064	0.006	0.002	0.341	0.002	0.006
Autism spectrum Fat	2	0.156	0.064	0.005	0.002	0.341	0.002	0.015
Autism spectrum Fat	3	0.141	0.064	0.004	0.002	0.341	0.002	0.029
Autism spectrum Fat	4	0.132	0.064	0.004	0.002	0.341	0.002	0.040
Autism spectrum Folate	0	-0.101	0.251	-0.001	0.002	0.406	0.002	0.688
Autism spectrum Folate	1	-0.089	0.250	-0.001	0.002	0.407	0.002	0.723
Autism spectrum Folate	2	-0.187	0.249	-0.002	0.002	0.407	0.002	0.452
Autism spectrum Folate	3	-0.179	0.249	-0.002	0.002	0.407	0.002	0.472
Autism spectrum Folate	4	-0.180	0.247	-0.002	0.002	0.405	0.002	0.467
Autism spectrum Food weight	0	-0.121	1.800	0.000	0.002	0.537	0.001	0.947
Autism spectrum Food weight	1	-0.346	1.798	0.000	0.002	0.536	0.001	0.848
Autism spectrum Food weight	2	-1.203	1.797	-0.002	0.002	0.536	0.001	0.503
Autism spectrum Food weight	3	-0.352	1.779	0.000	0.002	0.536	0.001	0.843
Autism spectrum Food weight	4	-0.160	1.755	0.000	0.002	0.534	0.001	0.927
Autism spectrum Iron	0	0.011	0.010	0.002	0.002	0.369	0.002	0.276
Autism spectrum Iron	1	0.011	0.010	0.002	0.002	0.369	0.002	0.242
Autism spectrum Iron	2	0.001	0.010	0.000	0.002	0.369	0.002	0.881
Autism spectrum Iron	3	0.006	0.010	0.001	0.002	0.369	0.002	0.537
Autism spectrum Iron	4	0.006	0.010	0.001	0.002	0.369	0.002	0.554
Autism spectrum Protein	0	0.006	0.054	0.000	0.002	0.292	0.002	0.916
Autism spectrum Protein	1	0.019	0.054	0.001	0.002	0.292	0.002	0.724
Autism spectrum Protein	2	0.028	0.054	0.001	0.002	0.292	0.002	0.610
Autism spectrum Protein	3	0.025	0.054	0.001	0.002	0.292	0.002	0.641
Autism spectrum Protein	4	0.021	0.054	0.001	0.002	0.292	0.002	0.691
Autism spectrum Vitamin B12	0	-0.020	0.009	-0.004	0.002	0.157	0.003	0.029
Autism spectrum Vitamin B12	1	-0.018	0.009	-0.004	0.002	0.157	0.003	0.051
Autism spectrum Vitamin B12	2	-0.020	0.009	-0.004	0.002	0.157	0.003	0.028
Autism spectrum Vitamin B12	3	-0.018	0.009	-0.004	0.002	0.157	0.003	0.046
Autism spectrum Vitamin B12	4	-0.018	0.009	-0.004	0.002	0.157	0.003	0.043
Autism spectrum Vitamin B6	0	-0.006	0.002	-0.007	0.002	0.339	0.002	0.000
Autism spectrum Vitamin B6	1	-0.005	0.002	-0.007	0.002	0.340	0.002	0.001
Autism spectrum Vitamin B6	2	-0.004	0.002	-0.005	0.002	0.340	0.002	0.011
Autism spectrum Vitamin B6	3	-0.004	0.002	-0.005	0.002	0.340	0.002	0.012
Autism spectrum Vitamin B6	4	-0.004	0.002	-0.005	0.002	0.340	0.002	0.012

Autism spectrum	Vitamin C	0	-0.277	0.235	-0.003	0.002	0.396	0.002	0.237
Autism spectrum	Vitamin C	1	-0.271	0.234	-0.002	0.002	0.400	0.002	0.246
Autism spectrum	Vitamin C	2	-0.486	0.232	-0.004	0.002	0.399	0.002	0.036
Autism spectrum	Vitamin C	3	-0.448	0.232	-0.004	0.002	0.399	0.002	0.053
Autism spectrum	Vitamin C	4	-0.433	0.230	-0.004	0.002	0.397	0.002	0.059
Autism spectrum	Vitamin D	0	-0.001	0.006	0.000	0.002	0.127	0.003	0.940
Autism spectrum	Vitamin D	1	0.000	0.006	0.000	0.002	0.127	0.003	0.973
Autism spectrum	Vitamin D	2	-0.001	0.006	0.000	0.002	0.127	0.003	0.811
Autism spectrum	Vitamin D	3	-0.001	0.006	0.000	0.002	0.127	0.003	0.913
Autism spectrum	Vitamin D	4	-0.001	0.006	0.000	0.002	0.127	0.003	0.901
Autism spectrum	Vitamin E	0	0.033	0.010	0.007	0.002	0.296	0.002	0.001
Autism spectrum	Vitamin E	1	0.033	0.010	0.007	0.002	0.296	0.002	0.001
Autism spectrum	Vitamin E	2	0.022	0.010	0.005	0.002	0.296	0.002	0.023
Autism spectrum	Vitamin E	3	0.021	0.010	0.004	0.002	0.296	0.002	0.033
Autism spectrum	Vitamin E	4	0.020	0.010	0.004	0.002	0.295	0.002	0.039
Bipolar disorder	Alcohol	0	0.197	0.050	0.009	0.002	0.479	0.002	0.000
Bipolar disorder	Alcohol	1	0.170	0.049	0.008	0.002	0.496	0.002	0.001
Bipolar disorder	Alcohol	2	0.147	0.049	0.007	0.002	0.497	0.002	0.003
Bipolar disorder	Alcohol	3	0.057	0.040	0.003	0.002	0.496	0.002	0.157
Bipolar disorder	Alcohol	4	0.059	0.040	0.003	0.002	0.497	0.002	0.144
Bipolar disorder	Calcium	0	2.515	0.820	0.007	0.002	0.340	0.002	0.002
Bipolar disorder	Calcium	1	2.608	0.820	0.007	0.002	0.340	0.002	0.002
Bipolar disorder	Calcium	2	2.301	0.820	0.006	0.002	0.340	0.002	0.005
Bipolar disorder	Calcium	3	2.534	0.816	0.007	0.002	0.340	0.002	0.002
Bipolar disorder	Calcium	4	2.641	0.813	0.007	0.002	0.339	0.002	0.001
Bipolar disorder	Carbohydrate	0	0.403	0.188	0.005	0.002	0.409	0.002	0.032
Bipolar disorder	Carbohydrate	1	0.429	0.188	0.005	0.002	0.410	0.002	0.023
Bipolar disorder	Carbohydrate	2	0.365	0.188	0.004	0.002	0.410	0.002	0.052
Bipolar disorder	Carbohydrate	3	0.444	0.186	0.005	0.002	0.409	0.002	0.017
Bipolar disorder	Carbohydrate	4	0.472	0.185	0.005	0.002	0.409	0.002	0.011
Bipolar disorder	Carotene	0	27.618	5.829	0.010	0.002	0.255	0.003	0.000
Bipolar disorder	Carotene	1	29.535	5.760	0.010	0.002	0.260	0.003	0.000
Bipolar disorder	Carotene	2	26.293	5.757	0.009	0.002	0.260	0.003	0.000
Bipolar disorder	Carotene	3	27.671	5.753	0.010	0.002	0.260	0.003	0.000
Bipolar disorder	Carotene	4	28.336	5.702	0.010	0.002	0.257	0.003	0.000
Bipolar disorder	Fibre	0	0.106	0.016	0.015	0.002	0.436	0.002	0.000
Bipolar disorder	Fibre	1	0.111	0.015	0.016	0.002	0.439	0.002	0.000
Bipolar disorder	Fibre	2	0.098	0.015	0.014	0.002	0.439	0.002	0.000
Bipolar disorder	Fibre	3	0.106	0.015	0.015	0.002	0.439	0.002	0.000
Bipolar disorder	Fibre	4	0.108	0.015	0.015	0.002	0.436	0.002	0.000
Bipolar disorder	Fat	0	0.235	0.067	0.007	0.002	0.335	0.002	0.000

Bipolar disorder	Fat	1	0.227	0.066	0.007	0.002	0.341	0.002	0.001
Bipolar disorder	Fat	2	0.204	0.066	0.006	0.002	0.341	0.002	0.002
Bipolar disorder	Fat	3	0.200	0.066	0.006	0.002	0.341	0.002	0.003
Bipolar disorder	Fat	4	0.213	0.066	0.007	0.002	0.341	0.002	0.001
Bipolar disorder	Folate	0	1.452	0.258	0.012	0.002	0.406	0.002	0.000
Bipolar disorder	Folate	1	1.516	0.256	0.013	0.002	0.407	0.002	0.000
Bipolar disorder	Folate	2	1.357	0.256	0.011	0.002	0.407	0.002	0.000
Bipolar disorder	Folate	3	1.407	0.256	0.012	0.002	0.407	0.002	0.000
Bipolar disorder	Folate	4	1.446	0.253	0.012	0.002	0.405	0.002	0.000
Bipolar disorder	Food weight	0	11.401	1.848	0.014	0.002	0.537	0.001	0.000
Bipolar disorder	Food weight	1	11.359	1.847	0.014	0.002	0.536	0.001	0.000
Bipolar disorder	Food weight	2	10.301	1.845	0.013	0.002	0.536	0.001	0.000
Bipolar disorder	Food weight	3	9.156	1.827	0.011	0.002	0.536	0.001	0.000
Bipolar disorder	Food weight	4	9.358	1.802	0.012	0.002	0.534	0.001	0.000
Bipolar disorder	Iron	0	0.065	0.010	0.014	0.002	0.369	0.002	0.000
Bipolar disorder	Iron	1	0.065	0.010	0.014	0.002	0.369	0.002	0.000
Bipolar disorder	Iron	2	0.051	0.010	0.011	0.002	0.369	0.002	0.000
Bipolar disorder	Iron	3	0.051	0.010	0.011	0.002	0.369	0.002	0.000
Bipolar disorder	Iron	4	0.052	0.010	0.011	0.002	0.369	0.002	0.000
Bipolar disorder	Protein	0	0.145	0.055	0.005	0.002	0.292	0.002	0.009
Bipolar disorder	Protein	1	0.150	0.055	0.006	0.002	0.292	0.002	0.007
Bipolar disorder	Protein	2	0.153	0.055	0.006	0.002	0.292	0.002	0.006
Bipolar disorder	Protein	3	0.154	0.055	0.006	0.002	0.292	0.002	0.005
Bipolar disorder	Protein	4	0.163	0.055	0.006	0.002	0.292	0.002	0.003
Bipolar disorder	Vitamin B12	0	0.035	0.009	0.007	0.002	0.157	0.003	0.000
Bipolar disorder	Vitamin B12	1	0.036	0.009	0.008	0.002	0.157	0.003	0.000
Bipolar disorder	Vitamin B12	2	0.032	0.009	0.007	0.002	0.157	0.003	0.001
Bipolar disorder	Vitamin B12	3	0.032	0.009	0.007	0.002	0.157	0.003	0.000
Bipolar disorder	Vitamin B12	4	0.033	0.009	0.007	0.002	0.157	0.003	0.000
Bipolar disorder	Vitamin B6	0	0.003	0.002	0.004	0.002	0.339	0.002	0.051
Bipolar disorder	Vitamin B6	1	0.004	0.002	0.005	0.002	0.340	0.002	0.025
Bipolar disorder	Vitamin B6	2	0.005	0.002	0.006	0.002	0.340	0.002	0.004
Bipolar disorder	Vitamin B6	3	0.005	0.002	0.006	0.002	0.340	0.002	0.003
Bipolar disorder	Vitamin B6	4	0.005	0.002	0.007	0.002	0.340	0.002	0.002
Bipolar disorder	Vitamin C	0	0.830	0.241	0.008	0.002	0.396	0.002	0.001
Bipolar disorder	Vitamin C	1	0.893	0.240	0.008	0.002	0.400	0.002	0.000
Bipolar disorder	Vitamin C	2	0.580	0.238	0.005	0.002	0.399	0.002	0.015
Bipolar disorder	Vitamin C	3	0.644	0.238	0.006	0.002	0.399	0.002	0.007
Bipolar disorder	Vitamin C	4	0.663	0.236	0.006	0.002	0.397	0.002	0.005
Bipolar disorder	Vitamin D	0	0.021	0.006	0.007	0.002	0.127	0.003	0.001
Bipolar disorder	Vitamin D	1	0.022	0.006	0.007	0.002	0.127	0.003	0.000

Bipolar disorder	Vitamin D	2	0.020	0.006	0.006	0.002	0.127	0.003	0.001
Bipolar disorder	Vitamin D	3	0.020	0.006	0.006	0.002	0.127	0.003	0.001
Bipolar disorder	Vitamin D	4	0.021	0.006	0.006	0.002	0.127	0.003	0.001
Bipolar disorder	Vitamin E	0	0.048	0.010	0.010	0.002	0.296	0.002	0.000
Bipolar disorder	Vitamin E	1	0.049	0.010	0.010	0.002	0.296	0.002	0.000
Bipolar disorder	Vitamin E	2	0.036	0.010	0.008	0.002	0.296	0.002	0.000
Bipolar disorder	Vitamin E	3	0.039	0.010	0.008	0.002	0.296	0.002	0.000
Bipolar disorder	Vitamin E	4	0.041	0.010	0.009	0.002	0.295	0.002	0.000
BMI	Alcohol	0	-0.158	0.049	-0.007	0.002	0.479	0.002	0.001
BMI	Alcohol	1	-0.118	0.048	-0.005	0.002	0.496	0.002	0.014
BMI	Alcohol	2	-0.084	0.048	-0.004	0.002	0.497	0.002	0.082
BMI	Alcohol	3	0.247	0.040	0.011	0.002	0.496	0.002	0.000
BMI	Alcohol	4	0.245	0.040	0.011	0.002	0.497	0.002	0.000
BMI	Calcium	0	-1.231	0.807	-0.003	0.002	0.340	0.002	0.127
BMI	Calcium	1	-0.960	0.807	-0.003	0.002	0.340	0.002	0.234
BMI	Calcium	2	-0.383	0.807	-0.001	0.002	0.340	0.002	0.635
BMI	Calcium	3	-1.076	0.804	-0.003	0.002	0.340	0.002	0.180
BMI	Calcium	4	-1.146	0.801	-0.003	0.002	0.339	0.002	0.152
BMI	Carbohydrate	0	-1.903	0.185	-0.022	0.002	0.409	0.002	0.000
BMI	Carbohydrate	1	-1.693	0.185	-0.019	0.002	0.410	0.002	0.000
BMI	Carbohydrate	2	-1.598	0.185	-0.018	0.002	0.410	0.002	0.000
BMI	Carbohydrate	3	-1.870	0.183	-0.021	0.002	0.409	0.002	0.000
BMI	Carbohydrate	4	-1.885	0.182	-0.022	0.002	0.409	0.002	0.000
BMI	Carotene	0	-4.179	5.732	-0.002	0.002	0.255	0.003	0.466
BMI	Carotene	1	-12.708	5.671	-0.004	0.002	0.260	0.003	0.025
BMI	Carotene	2	-7.764	5.670	-0.003	0.002	0.260	0.003	0.171
BMI	Carotene	3	-6.503	5.671	-0.002	0.002	0.260	0.003	0.252
BMI	Carotene	4	-7.504	5.621	-0.003	0.002	0.257	0.003	0.182
BMI	Fibre	0	-0.068	0.015	-0.010	0.002	0.436	0.002	0.000
BMI	Fibre	1	-0.087	0.015	-0.012	0.002	0.439	0.002	0.000
BMI	Fibre	2	-0.066	0.015	-0.009	0.002	0.439	0.002	0.000
BMI	Fibre	3	-0.074	0.015	-0.011	0.002	0.439	0.002	0.000
BMI	Fibre	4	-0.076	0.015	-0.011	0.002	0.436	0.002	0.000
BMI	Fat	0	-0.581	0.066	-0.018	0.002	0.335	0.002	0.000
BMI	Fat	1	-0.435	0.065	-0.014	0.002	0.341	0.002	0.000
BMI	Fat	2	-0.403	0.065	-0.013	0.002	0.341	0.002	0.000
BMI	Fat	3	-0.435	0.065	-0.014	0.002	0.341	0.002	0.000
BMI	Fat	4	-0.433	0.065	-0.014	0.002	0.341	0.002	0.000
BMI	Folate	0	-1.732	0.253	-0.015	0.002	0.406	0.002	0.000
BMI	Folate	1	-1.871	0.252	-0.016	0.002	0.407	0.002	0.000
BMI	Folate	2	-1.581	0.252	-0.013	0.002	0.407	0.002	0.000

BMI	Folate	3	-1.508	0.252	-0.013	0.002	0.407	0.002	0.000
BMI	Folate	4	-1.547	0.250	-0.013	0.002	0.405	0.002	0.000
BMI	Food weight	0	9.669	1.817	0.012	0.002	0.537	0.001	0.000
BMI	Food weight	1	8.557	1.816	0.011	0.002	0.536	0.001	0.000
BMI	Food weight	2	10.391	1.816	0.013	0.002	0.536	0.001	0.000
BMI	Food weight	3	13.217	1.799	0.016	0.002	0.536	0.001	0.000
BMI	Food weight	4	12.783	1.774	0.016	0.002	0.534	0.001	0.000
BMI	Iron	0	-0.096	0.010	-0.021	0.002	0.369	0.002	0.000
BMI	Iron	1	-0.093	0.010	-0.020	0.002	0.369	0.002	0.000
BMI	Iron	2	-0.071	0.010	-0.015	0.002	0.369	0.002	0.000
BMI	Iron	3	-0.051	0.010	-0.011	0.002	0.369	0.002	0.000
BMI	Iron	4	-0.052	0.010	-0.011	0.002	0.368	0.002	0.000
BMI	Protein	0	0.098	0.055	0.004	0.002	0.292	0.002	0.071
BMI	Protein	1	0.163	0.054	0.006	0.002	0.292	0.002	0.003
BMI	Protein	2	0.179	0.054	0.007	0.002	0.292	0.002	0.001
BMI	Protein	3	0.178	0.055	0.007	0.002	0.292	0.002	0.001
BMI	Protein	4	0.177	0.054	0.007	0.002	0.292	0.002	0.001
BMI	Vitamin B12	0	-0.006	0.009	-0.001	0.002	0.157	0.003	0.543
BMI	Vitamin B12	1	0.003	0.009	0.001	0.002	0.157	0.003	0.715
BMI	Vitamin B12	2	0.012	0.009	0.003	0.002	0.157	0.003	0.193
BMI	Vitamin B12	3	0.017	0.009	0.004	0.002	0.157	0.003	0.063
BMI	Vitamin B12	4	0.017	0.009	0.004	0.002	0.157	0.003	0.066
BMI	Vitamin B6	0	-0.001	0.002	-0.001	0.002	0.339	0.002	0.700
BMI	Vitamin B6	1	0.000	0.002	0.000	0.002	0.340	0.002	0.831
BMI	Vitamin B6	2	-0.001	0.002	-0.001	0.002	0.340	0.002	0.671
BMI	Vitamin B6	3	0.000	0.002	0.000	0.002	0.340	0.002	0.947
BMI	Vitamin B6	4	0.000	0.002	0.000	0.002	0.340	0.002	0.862
BMI	Vitamin C	0	-0.952	0.237	-0.009	0.002	0.396	0.002	0.000
BMI	Vitamin C	1	-1.116	0.236	-0.010	0.002	0.400	0.002	0.000
BMI	Vitamin C	2	-0.607	0.234	-0.006	0.002	0.399	0.002	0.010
BMI	Vitamin C	3	-0.471	0.234	-0.004	0.002	0.399	0.002	0.044
BMI	Vitamin C	4	-0.517	0.232	-0.005	0.002	0.397	0.002	0.026
BMI	Vitamin D	0	-0.010	0.006	-0.003	0.002	0.127	0.003	0.099
BMI	Vitamin D	1	-0.008	0.006	-0.003	0.002	0.127	0.003	0.163
BMI	Vitamin D	2	-0.004	0.006	-0.001	0.002	0.127	0.003	0.539
BMI	Vitamin D	3	-0.001	0.006	0.000	0.002	0.127	0.003	0.830
BMI	Vitamin D	4	-0.002	0.006	-0.001	0.002	0.127	0.003	0.795
BMI	Vitamin E	0	-0.079	0.010	-0.017	0.002	0.296	0.002	0.000
BMI	Vitamin E	1	-0.085	0.010	-0.018	0.002	0.295	0.002	0.000
BMI	Vitamin E	2	-0.064	0.010	-0.013	0.002	0.296	0.002	0.000
BMI	Vitamin E	3	-0.065	0.010	-0.014	0.002	0.296	0.002	0.000

BMI	Vitamin E	4	-0.066	0.010	-0.014	0.002	0.295	0.002	0.000
Educational attai	Alcohol	0	0.202	0.050	0.009	0.002	0.479	0.002	0.000
Educational attai	Alcohol	1	0.137	0.050	0.006	0.002	0.496	0.002	0.006
Educational attai	Alcohol	2	0.038	0.050	0.002	0.002	0.497	0.002	0.448
Educational attai	Alcohol	3	-0.178	0.041	-0.008	0.002	0.496	0.002	0.000
Educational attai	Alcohol	4	-0.175	0.041	-0.008	0.002	0.497	0.002	0.000
Educational attai	Calcium	0	5.316	0.832	0.014	0.002	0.340	0.002	0.000
Educational attai	Calcium	1	5.348	0.832	0.014	0.002	0.340	0.002	0.000
Educational attai	Calcium	2	3.745	0.839	0.010	0.002	0.340	0.002	0.000
Educational attai	Calcium	3	4.185	0.835	0.011	0.002	0.340	0.002	0.000
Educational attai	Calcium	4	4.194	0.832	0.011	0.002	0.339	0.002	0.000
Educational attai	Carbohydrate	0	1.472	0.191	0.017	0.002	0.409	0.002	0.000
Educational attai	Carbohydrate	1	1.416	0.191	0.016	0.002	0.410	0.002	0.000
Educational attai	Carbohydrate	2	1.136	0.192	0.013	0.002	0.410	0.002	0.000
Educational attai	Carbohydrate	3	1.318	0.190	0.015	0.002	0.409	0.002	0.000
Educational attai	Carbohydrate	4	1.331	0.189	0.015	0.002	0.409	0.002	0.000
Educational attai	Carotene	0	22.363	5.913	0.008	0.002	0.255	0.003	0.000
Educational attai	Carotene	1	29.628	5.844	0.010	0.002	0.260	0.003	0.000
Educational attai	Carotene	2	14.673	5.892	0.005	0.002	0.260	0.003	0.013
Educational attai	Carotene	3	13.504	5.890	0.005	0.002	0.260	0.003	0.022
Educational attai	Carotene	4	13.703	5.838	0.005	0.002	0.257	0.003	0.019
Educational attai	Fibre	0	0.142	0.016	0.020	0.002	0.436	0.002	0.000
Educational attai	Fibre	1	0.160	0.016	0.023	0.002	0.439	0.002	0.000
Educational attai	Fibre	2	0.099	0.016	0.014	0.002	0.439	0.002	0.000
Educational attai	Fibre	3	0.104	0.016	0.015	0.002	0.439	0.002	0.000
Educational attai	Fibre	4	0.104	0.015	0.015	0.002	0.436	0.002	0.000
Educational attai	Fat	0	0.564	0.068	0.018	0.002	0.335	0.002	0.000
Educational attai	Fat	1	0.480	0.067	0.015	0.002	0.341	0.002	0.000
Educational attai	Fat	2	0.388	0.068	0.012	0.002	0.341	0.002	0.000
Educational attai	Fat	3	0.411	0.068	0.013	0.002	0.341	0.002	0.000
Educational attai	Fat	4	0.411	0.067	0.013	0.002	0.341	0.002	0.000
Educational attai	Folate	0	2.366	0.261	0.020	0.002	0.406	0.002	0.000
Educational attai	Folate	1	2.539	0.260	0.021	0.002	0.407	0.002	0.000
Educational attai	Folate	2	1.721	0.262	0.015	0.002	0.407	0.002	0.000
Educational attai	Folate	3	1.659	0.262	0.014	0.002	0.407	0.002	0.000
Educational attai	Folate	4	1.665	0.259	0.014	0.002	0.405	0.002	0.000
Educational attai	Food weight	0	11.267	1.874	0.014	0.002	0.537	0.001	0.000
Educational attai	Food weight	1	11.720	1.873	0.015	0.002	0.536	0.001	0.000
Educational attai	Food weight	2	6.525	1.888	0.008	0.002	0.536	0.001	0.001
Educational attai	Food weight	3	4.712	1.870	0.006	0.002	0.536	0.001	0.012
Educational attai	Food weight	4	5.027	1.844	0.006	0.002	0.534	0.001	0.006

Educational attai	Iron	0	0.181	0.010	0.039	0.002	0.369	0.002	0.000
Educational attai	Iron	1	0.180	0.010	0.039	0.002	0.369	0.002	0.000
Educational attai	Iron	2	0.116	0.010	0.025	0.002	0.369	0.002	0.000
Educational attai	Iron	3	0.102	0.010	0.022	0.002	0.369	0.002	0.000
Educational attai	Iron	4	0.102	0.010	0.022	0.002	0.369	0.002	0.000
Educational attai	Protein	0	0.232	0.056	0.009	0.002	0.292	0.002	0.000
Educational attai	Protein	1	0.209	0.056	0.008	0.002	0.292	0.002	0.000
Educational attai	Protein	2	0.182	0.057	0.007	0.002	0.292	0.002	0.001
Educational attai	Protein	3	0.182	0.057	0.007	0.002	0.292	0.002	0.001
Educational attai	Protein	4	0.180	0.056	0.007	0.002	0.292	0.002	0.001
Educational attai	Vitamin B12	0	0.055	0.009	0.012	0.002	0.157	0.003	0.000
Educational attai	Vitamin B12	1	0.053	0.009	0.011	0.002	0.157	0.003	0.000
Educational attai	Vitamin B12	2	0.030	0.009	0.006	0.002	0.157	0.003	0.002
Educational attai	Vitamin B12	3	0.026	0.009	0.005	0.002	0.157	0.003	0.006
Educational attai	Vitamin B12	4	0.026	0.009	0.005	0.002	0.157	0.003	0.006
Educational attai	Vitamin B6	0	0.000	0.002	0.000	0.002	0.339	0.002	0.859
Educational attai	Vitamin B6	1	0.000	0.002	0.001	0.002	0.340	0.002	0.821
Educational attai	Vitamin B6	2	0.002	0.002	0.003	0.002	0.340	0.002	0.146
Educational attai	Vitamin B6	3	0.002	0.002	0.003	0.002	0.340	0.002	0.229
Educational attai	Vitamin B6	4	0.002	0.002	0.003	0.002	0.340	0.002	0.221
Educational attai	Vitamin C	0	2.308	0.244	0.021	0.002	0.396	0.002	0.000
Educational attai	Vitamin C	1	2.492	0.243	0.023	0.002	0.400	0.002	0.000
Educational attai	Vitamin C	2	0.993	0.244	0.009	0.002	0.400	0.002	0.000
Educational attai	Vitamin C	3	0.886	0.243	0.008	0.002	0.399	0.002	0.000
Educational attai	Vitamin C	4	0.898	0.241	0.008	0.002	0.397	0.002	0.000
Educational attai	Vitamin D	0	0.036	0.006	0.011	0.002	0.127	0.003	0.000
Educational attai	Vitamin D	1	0.037	0.006	0.011	0.002	0.127	0.003	0.000
Educational attai	Vitamin D	2	0.023	0.006	0.007	0.002	0.127	0.003	0.000
Educational attai	Vitamin D	3	0.022	0.006	0.007	0.002	0.127	0.003	0.001
Educational attai	Vitamin D	4	0.022	0.006	0.007	0.002	0.127	0.003	0.001
Educational attai	Vitamin E	0	0.139	0.010	0.029	0.002	0.296	0.002	0.000
Educational attai	Vitamin E	1	0.143	0.010	0.030	0.002	0.296	0.002	0.000
Educational attai	Vitamin E	2	0.082	0.010	0.017	0.002	0.296	0.002	0.000
Educational attai	Vitamin E	3	0.081	0.010	0.017	0.002	0.296	0.002	0.000
Educational attai	Vitamin E	4	0.081	0.010	0.017	0.002	0.295	0.002	0.000
Food addiction	Alcohol	0	-0.038	0.049	-0.002	0.002	0.479	0.002	0.438
Food addiction	Alcohol	1	-0.041	0.048	-0.002	0.002	0.496	0.002	0.399
Food addiction	Alcohol	2	-0.039	0.048	-0.002	0.002	0.497	0.002	0.415
Food addiction	Alcohol	3	0.010	0.040	0.000	0.002	0.496	0.002	0.802
Food addiction	Alcohol	4	0.010	0.040	0.000	0.002	0.497	0.002	0.801
Food addiction	Calcium	0	1.240	0.805	0.003	0.002	0.340	0.002	0.123

Food addiction	Calcium	1	1.275	0.804	0.003	0.002	0.340	0.002	0.113
Food addiction	Calcium	2	1.314	0.803	0.003	0.002	0.340	0.002	0.102
Food addiction	Calcium	3	1.206	0.799	0.003	0.002	0.340	0.002	0.131
Food addiction	Calcium	4	1.264	0.796	0.003	0.002	0.339	0.002	0.112
Food addiction	Carbohydrate	0	0.202	0.185	0.002	0.002	0.409	0.002	0.275
Food addiction	Carbohydrate	1	0.218	0.184	0.003	0.002	0.410	0.002	0.236
Food addiction	Carbohydrate	2	0.225	0.184	0.003	0.002	0.410	0.002	0.223
Food addiction	Carbohydrate	3	0.187	0.182	0.002	0.002	0.409	0.002	0.306
Food addiction	Carbohydrate	4	0.200	0.181	0.002	0.002	0.409	0.002	0.269
Food addiction	Carotene	0	-0.077	5.716	0.000	0.002	0.255	0.003	0.989
Food addiction	Carotene	1	-0.129	5.649	0.000	0.002	0.260	0.003	0.982
Food addiction	Carotene	2	0.134	5.641	0.000	0.002	0.260	0.003	0.981
Food addiction	Carotene	3	0.086	5.636	0.000	0.002	0.260	0.003	0.988
Food addiction	Carotene	4	0.546	5.586	0.000	0.002	0.257	0.003	0.922
Food addiction	Fibre	0	0.006	0.015	0.001	0.002	0.436	0.002	0.719
Food addiction	Fibre	1	0.006	0.015	0.001	0.002	0.439	0.002	0.715
Food addiction	Fibre	2	0.007	0.015	0.001	0.002	0.439	0.002	0.653
Food addiction	Fibre	3	0.005	0.015	0.001	0.002	0.439	0.002	0.742
Food addiction	Fibre	4	0.006	0.015	0.001	0.002	0.436	0.002	0.679
Food addiction	Fat	0	0.107	0.065	0.003	0.002	0.335	0.002	0.100
Food addiction	Fat	1	0.114	0.065	0.004	0.002	0.341	0.002	0.078
Food addiction	Fat	2	0.116	0.065	0.004	0.002	0.341	0.002	0.073
Food addiction	Fat	3	0.114	0.065	0.004	0.002	0.341	0.002	0.079
Food addiction	Fat	4	0.118	0.065	0.004	0.002	0.341	0.002	0.068
Food addiction	Folate	0	0.038	0.253	0.000	0.002	0.406	0.002	0.882
Food addiction	Folate	1	0.043	0.251	0.000	0.002	0.407	0.002	0.865
Food addiction	Folate	2	0.063	0.251	0.001	0.002	0.407	0.002	0.801
Food addiction	Folate	3	0.061	0.250	0.001	0.002	0.407	0.002	0.807
Food addiction	Folate	4	0.082	0.248	0.001	0.002	0.405	0.002	0.742
Food addiction	Food weight	0	-0.138	1.813	0.000	0.002	0.537	0.001	0.939
Food addiction	Food weight	1	-0.206	1.811	0.000	0.002	0.536	0.001	0.910
Food addiction	Food weight	2	-0.106	1.808	0.000	0.002	0.536	0.001	0.953
Food addiction	Food weight	3	0.239	1.790	0.000	0.002	0.536	0.001	0.894
Food addiction	Food weight	4	0.364	1.765	0.000	0.002	0.534	0.001	0.837
Food addiction	Iron	0	0.000	0.010	0.000	0.002	0.369	0.002	0.985
Food addiction	Iron	1	0.000	0.010	0.000	0.002	0.369	0.002	0.964
Food addiction	Iron	2	0.002	0.010	0.000	0.002	0.369	0.002	0.846
Food addiction	Iron	3	0.004	0.010	0.001	0.002	0.369	0.002	0.692
Food addiction	Iron	4	0.004	0.010	0.001	0.002	0.369	0.002	0.649
Food addiction	Protein	0	0.029	0.054	0.001	0.002	0.292	0.002	0.600
Food addiction	Protein	1	0.033	0.054	0.001	0.002	0.292	0.002	0.541

Food addiction	Protein	2	0.035	0.054	0.001	0.002	0.292	0.002	0.515
Food addiction	Protein	3	0.033	0.054	0.001	0.002	0.292	0.002	0.541
Food addiction	Protein	4	0.036	0.054	0.001	0.002	0.292	0.002	0.501
Food addiction	Vitamin B12	0	0.001	0.009	0.000	0.002	0.157	0.003	0.921
Food addiction	Vitamin B12	1	0.002	0.009	0.000	0.002	0.157	0.003	0.853
Food addiction	Vitamin B12	2	0.002	0.009	0.001	0.002	0.157	0.003	0.789
Food addiction	Vitamin B12	3	0.003	0.009	0.001	0.002	0.157	0.003	0.755
Food addiction	Vitamin B12	4	0.003	0.009	0.001	0.002	0.157	0.003	0.741
Food addiction	Vitamin B6	0	0.000	0.002	0.001	0.002	0.339	0.002	0.814
Food addiction	Vitamin B6	1	0.001	0.002	0.001	0.002	0.340	0.002	0.759
Food addiction	Vitamin B6	2	0.001	0.002	0.001	0.002	0.340	0.002	0.720
Food addiction	Vitamin B6	3	0.001	0.002	0.001	0.002	0.340	0.002	0.724
Food addiction	Vitamin B6	4	0.001	0.002	0.001	0.002	0.340	0.002	0.675
Food addiction	Vitamin C	0	-0.178	0.236	-0.002	0.002	0.396	0.002	0.451
Food addiction	Vitamin C	1	-0.175	0.235	-0.002	0.002	0.400	0.002	0.456
Food addiction	Vitamin C	2	-0.143	0.233	-0.001	0.002	0.399	0.002	0.539
Food addiction	Vitamin C	3	-0.137	0.233	-0.001	0.002	0.399	0.002	0.555
Food addiction	Vitamin C	4	-0.125	0.231	-0.001	0.002	0.397	0.002	0.588
Food addiction	Vitamin D	0	0.002	0.006	0.001	0.002	0.127	0.003	0.745
Food addiction	Vitamin D	1	0.002	0.006	0.001	0.002	0.127	0.003	0.714
Food addiction	Vitamin D	2	0.003	0.006	0.001	0.002	0.127	0.003	0.668
Food addiction	Vitamin D	3	0.003	0.006	0.001	0.002	0.127	0.003	0.654
Food addiction	Vitamin D	4	0.003	0.006	0.001	0.002	0.127	0.003	0.637
Food addiction	Vitamin E	0	0.005	0.010	0.001	0.002	0.296	0.002	0.580
Food addiction	Vitamin E	1	0.005	0.010	0.001	0.002	0.296	0.002	0.590
Food addiction	Vitamin E	2	0.006	0.010	0.001	0.002	0.296	0.002	0.508
Food addiction	Vitamin E	3	0.006	0.010	0.001	0.002	0.296	0.002	0.540
Food addiction	Vitamin E	4	0.007	0.010	0.001	0.002	0.295	0.002	0.485
Height	Alcohol	0	0.260	0.070	0.011	0.003	0.479	0.002	0.000
Height	Alcohol	1	0.248	0.069	0.011	0.003	0.496	0.002	0.000
Height	Alcohol	2	0.218	0.069	0.010	0.003	0.497	0.002	0.002
Height	Alcohol	3	0.156	0.057	0.007	0.003	0.496	0.002	0.006
Height	Alcohol	4	0.161	0.057	0.007	0.003	0.497	0.002	0.005
Height	Calcium	0	15.677	1.151	0.041	0.003	0.340	0.002	0.000
Height	Calcium	1	15.641	1.150	0.041	0.003	0.340	0.002	0.000
Height	Calcium	2	15.120	1.150	0.039	0.003	0.340	0.002	0.000
Height	Calcium	3	15.299	1.144	0.040	0.003	0.340	0.002	0.000
Height	Calcium	4	15.515	1.139	0.040	0.003	0.339	0.002	0.000
Height	Carbohydrate	0	4.534	0.264	0.052	0.003	0.409	0.002	0.000
Height	Carbohydrate	1	4.496	0.264	0.051	0.003	0.410	0.002	0.000
Height	Carbohydrate	2	4.409	0.264	0.050	0.003	0.410	0.002	0.000

Height	Carbohydrate	3	4.472	0.261	0.051	0.003	0.409	0.002	0.000
Height	Carbohydrate	4	4.524	0.259	0.052	0.003	0.409	0.002	0.000
Height	Carotene	0	42.250	8.180	0.015	0.003	0.255	0.003	0.000
Height	Carotene	1	44.394	8.084	0.016	0.003	0.260	0.003	0.000
Height	Carotene	2	39.964	8.077	0.014	0.003	0.260	0.003	0.000
Height	Carotene	3	39.216	8.070	0.014	0.003	0.260	0.003	0.000
Height	Carotene	4	41.219	7.999	0.014	0.003	0.257	0.003	0.000
Height	Fibre	0	0.272	0.022	0.039	0.003	0.436	0.002	0.000
Height	Fibre	1	0.277	0.022	0.040	0.003	0.439	0.002	0.000
Height	Fibre	2	0.258	0.021	0.037	0.003	0.439	0.002	0.000
Height	Fibre	3	0.260	0.021	0.037	0.003	0.439	0.002	0.000
Height	Fibre	4	0.265	0.021	0.038	0.003	0.436	0.002	0.000
Height	Fat	0	1.547	0.093	0.049	0.003	0.335	0.002	0.000
Height	Fat	1	1.515	0.093	0.048	0.003	0.341	0.002	0.000
Height	Fat	2	1.487	0.093	0.047	0.003	0.341	0.002	0.000
Height	Fat	3	1.497	0.093	0.047	0.003	0.341	0.002	0.000
Height	Fat	4	1.508	0.092	0.047	0.003	0.341	0.002	0.000
Height	Folate	0	4.491	0.361	0.038	0.003	0.406	0.002	0.000
Height	Folate	1	4.530	0.360	0.038	0.003	0.407	0.002	0.000
Height	Folate	2	4.263	0.359	0.036	0.003	0.407	0.002	0.000
Height	Folate	3	4.244	0.358	0.036	0.003	0.407	0.002	0.000
Height	Folate	4	4.338	0.355	0.037	0.003	0.405	0.002	0.000
Height	Food weight	0	36.691	2.593	0.045	0.003	0.537	0.001	0.000
Height	Food weight	1	36.908	2.591	0.046	0.003	0.536	0.001	0.000
Height	Food weight	2	35.307	2.588	0.044	0.003	0.536	0.001	0.000
Height	Food weight	3	34.687	2.562	0.043	0.003	0.536	0.001	0.000
Height	Food weight	4	35.449	2.527	0.044	0.003	0.534	0.001	0.000
Height	Iron	0	0.231	0.014	0.049	0.003	0.369	0.002	0.000
Height	Iron	1	0.231	0.014	0.049	0.003	0.369	0.002	0.000
Height	Iron	2	0.210	0.014	0.045	0.003	0.369	0.002	0.000
Height	Iron	3	0.207	0.014	0.044	0.003	0.369	0.002	0.000
Height	Iron	4	0.210	0.014	0.045	0.003	0.369	0.002	0.000
Height	Protein	0	0.918	0.078	0.034	0.003	0.292	0.002	0.000
Height	Protein	1	0.906	0.078	0.034	0.003	0.292	0.002	0.000
Height	Protein	2	0.891	0.078	0.033	0.003	0.292	0.002	0.000
Height	Protein	3	0.891	0.078	0.033	0.003	0.292	0.002	0.000
Height	Protein	4	0.904	0.077	0.034	0.003	0.292	0.002	0.000
Height	Vitamin B12	0	0.065	0.013	0.014	0.003	0.157	0.003	0.000
Height	Vitamin B12	1	0.064	0.013	0.014	0.003	0.157	0.003	0.000
Height	Vitamin B12	2	0.056	0.013	0.012	0.003	0.157	0.003	0.000
Height	Vitamin B12	3	0.055	0.013	0.012	0.003	0.157	0.003	0.000

Height	Vitamin B12	4	0.056	0.013	0.012	0.003	0.157	0.003	0.000
Height	Vitamin B6	0	0.025	0.002	0.032	0.003	0.339	0.002	0.000
Height	Vitamin B6	1	0.025	0.002	0.032	0.003	0.340	0.002	0.000
Height	Vitamin B6	2	0.025	0.002	0.032	0.003	0.340	0.002	0.000
Height	Vitamin B6	3	0.025	0.002	0.032	0.003	0.340	0.002	0.000
Height	Vitamin B6	4	0.026	0.002	0.033	0.003	0.340	0.002	0.000
Height	Vitamin C	0	2.484	0.338	0.022	0.003	0.396	0.002	0.000
Height	Vitamin C	1	2.527	0.336	0.023	0.003	0.400	0.002	0.000
Height	Vitamin C	2	2.059	0.334	0.019	0.003	0.400	0.002	0.000
Height	Vitamin C	3	2.019	0.333	0.018	0.003	0.399	0.002	0.000
Height	Vitamin C	4	2.095	0.331	0.019	0.003	0.397	0.002	0.000
Height	Vitamin D	0	0.029	0.009	0.009	0.003	0.127	0.003	0.001
Height	Vitamin D	1	0.029	0.009	0.009	0.003	0.127	0.003	0.001
Height	Vitamin D	2	0.025	0.009	0.008	0.003	0.127	0.003	0.004
Height	Vitamin D	3	0.024	0.009	0.007	0.003	0.127	0.003	0.006
Height	Vitamin D	4	0.025	0.009	0.008	0.003	0.127	0.003	0.004
Height	Vitamin E	0	0.190	0.014	0.040	0.003	0.296	0.002	0.000
Height	Vitamin E	1	0.192	0.014	0.040	0.003	0.296	0.002	0.000
Height	Vitamin E	2	0.173	0.014	0.036	0.003	0.296	0.002	0.000
Height	Vitamin E	3	0.173	0.014	0.036	0.003	0.296	0.002	0.000
Height	Vitamin E	4	0.176	0.014	0.037	0.003	0.295	0.002	0.000
Lupus	Alcohol	0	0.007	0.050	0.000	0.002	0.479	0.002	0.894
Lupus	Alcohol	1	0.021	0.049	0.001	0.002	0.496	0.002	0.665
Lupus	Alcohol	2	0.023	0.049	0.001	0.002	0.497	0.002	0.645
Lupus	Alcohol	3	0.016	0.040	0.001	0.002	0.496	0.002	0.698
Lupus	Alcohol	4	0.016	0.040	0.001	0.002	0.497	0.002	0.688
Lupus	Calcium	0	0.101	0.817	0.000	0.002	0.340	0.002	0.901
Lupus	Calcium	1	0.089	0.817	0.000	0.002	0.340	0.002	0.913
Lupus	Calcium	2	0.131	0.816	0.000	0.002	0.340	0.002	0.872
Lupus	Calcium	3	0.147	0.812	0.000	0.002	0.340	0.002	0.856
Lupus	Calcium	4	0.203	0.809	0.001	0.002	0.339	0.002	0.802
Lupus	Carbohydrate	0	-0.124	0.188	-0.001	0.002	0.409	0.002	0.509
Lupus	Carbohydrate	1	-0.114	0.187	-0.001	0.002	0.410	0.002	0.543
Lupus	Carbohydrate	2	-0.107	0.187	-0.001	0.002	0.410	0.002	0.567
Lupus	Carbohydrate	3	-0.100	0.185	-0.001	0.002	0.409	0.002	0.590
Lupus	Carbohydrate	4	-0.094	0.184	-0.001	0.002	0.409	0.002	0.611
Lupus	Carotene	0	-3.887	5.806	-0.001	0.002	0.255	0.003	0.503
Lupus	Carotene	1	-5.474	5.738	-0.002	0.002	0.260	0.003	0.340
Lupus	Carotene	2	-5.159	5.731	-0.002	0.002	0.260	0.003	0.368
Lupus	Carotene	3	-4.200	5.726	-0.002	0.002	0.260	0.003	0.463
Lupus	Carotene	4	-3.642	5.675	-0.001	0.002	0.257	0.003	0.521

Lupus	Fibre	0	0.000	0.015	0.000	0.002	0.436	0.002	0.981
Lupus	Fibre	1	-0.004	0.015	-0.001	0.002	0.439	0.002	0.778
Lupus	Fibre	2	-0.003	0.015	0.000	0.002	0.439	0.002	0.849
Lupus	Fibre	3	0.000	0.015	0.000	0.002	0.439	0.002	0.993
Lupus	Fibre	4	0.001	0.015	0.000	0.002	0.436	0.002	0.934
Lupus	Fat	0	-0.106	0.066	-0.003	0.002	0.335	0.002	0.112
Lupus	Fat	1	-0.088	0.066	-0.003	0.002	0.341	0.002	0.181
Lupus	Fat	2	-0.087	0.066	-0.003	0.002	0.341	0.002	0.187
Lupus	Fat	3	-0.095	0.066	-0.003	0.002	0.341	0.002	0.151
Lupus	Fat	4	-0.091	0.066	-0.003	0.002	0.341	0.002	0.163
Lupus	Folate	0	0.019	0.257	0.000	0.002	0.406	0.002	0.942
Lupus	Folate	1	-0.020	0.255	0.000	0.002	0.407	0.002	0.936
Lupus	Folate	2	-0.001	0.255	0.000	0.002	0.407	0.002	0.996
Lupus	Folate	3	0.030	0.254	0.000	0.002	0.407	0.002	0.905
Lupus	Folate	4	0.051	0.252	0.000	0.002	0.405	0.002	0.840
Lupus	Food weight	0	3.991	1.841	0.005	0.002	0.537	0.001	0.030
Lupus	Food weight	1	3.903	1.839	0.005	0.002	0.536	0.001	0.034
Lupus	Food weight	2	3.995	1.836	0.005	0.002	0.536	0.001	0.030
Lupus	Food weight	3	3.712	1.818	0.005	0.002	0.536	0.001	0.041
Lupus	Food weight	4	3.799	1.793	0.005	0.002	0.534	0.001	0.034
Lupus	Iron	0	-0.011	0.010	-0.002	0.002	0.369	0.002	0.290
Lupus	Iron	1	-0.011	0.010	-0.002	0.002	0.369	0.002	0.290
Lupus	Iron	2	-0.009	0.010	-0.002	0.002	0.369	0.002	0.359
Lupus	Iron	3	-0.007	0.010	-0.002	0.002	0.369	0.002	0.446
Lupus	Iron	4	-0.007	0.010	-0.001	0.002	0.369	0.002	0.493
Lupus	Protein	0	-0.014	0.055	-0.001	0.002	0.292	0.002	0.802
Lupus	Protein	1	-0.009	0.055	0.000	0.002	0.292	0.002	0.865
Lupus	Protein	2	-0.008	0.055	0.000	0.002	0.292	0.002	0.882
Lupus	Protein	3	-0.010	0.055	0.000	0.002	0.292	0.002	0.861
Lupus	Protein	4	-0.006	0.055	0.000	0.002	0.292	0.002	0.918
Lupus	Vitamin B12	0	0.002	0.009	0.001	0.002	0.157	0.003	0.810
Lupus	Vitamin B12	1	0.003	0.009	0.001	0.002	0.157	0.003	0.773
Lupus	Vitamin B12	2	0.003	0.009	0.001	0.002	0.157	0.003	0.722
Lupus	Vitamin B12	3	0.004	0.009	0.001	0.002	0.157	0.003	0.686
Lupus	Vitamin B12	4	0.004	0.009	0.001	0.002	0.157	0.003	0.636
Lupus	Vitamin B6	0	0.000	0.002	0.000	0.002	0.339	0.002	0.923
Lupus	Vitamin B6	1	0.000	0.002	0.000	0.002	0.340	0.002	0.841
Lupus	Vitamin B6	2	0.000	0.002	0.000	0.002	0.340	0.002	0.849
Lupus	Vitamin B6	3	0.000	0.002	0.000	0.002	0.340	0.002	0.904
Lupus	Vitamin B6	4	0.000	0.002	0.000	0.002	0.340	0.002	0.966
Lupus	Vitamin C	0	0.054	0.240	0.001	0.002	0.396	0.002	0.821

Lupus	Vitamin C	1	0.013	0.239	0.000	0.002	0.400	0.002	0.955
Lupus	Vitamin C	2	0.048	0.237	0.000	0.002	0.399	0.002	0.840
Lupus	Vitamin C	3	0.101	0.237	0.001	0.002	0.399	0.002	0.670
Lupus	Vitamin C	4	0.118	0.235	0.001	0.002	0.397	0.002	0.615
Lupus	Vitamin D	0	-0.005	0.006	-0.002	0.002	0.127	0.003	0.430
Lupus	Vitamin D	1	-0.005	0.006	-0.002	0.002	0.127	0.003	0.419
Lupus	Vitamin D	2	-0.005	0.006	-0.001	0.002	0.127	0.003	0.447
Lupus	Vitamin D	3	-0.004	0.006	-0.001	0.002	0.127	0.003	0.486
Lupus	Vitamin D	4	-0.004	0.006	-0.001	0.002	0.127	0.003	0.523
Lupus	Vitamin E	0	-0.010	0.010	-0.002	0.002	0.296	0.002	0.309
Lupus	Vitamin E	1	-0.011	0.010	-0.002	0.002	0.296	0.002	0.269
Lupus	Vitamin E	2	-0.010	0.010	-0.002	0.002	0.296	0.002	0.322
Lupus	Vitamin E	3	-0.008	0.010	-0.002	0.002	0.296	0.002	0.398
Lupus	Vitamin E	4	-0.007	0.010	-0.002	0.002	0.295	0.002	0.446
Major depressive	Alcohol	0	0.018	0.048	0.001	0.002	0.479	0.002	0.711
Major depressive	Alcohol	1	0.027	0.048	0.001	0.002	0.496	0.002	0.567
Major depressive	Alcohol	2	0.039	0.048	0.002	0.002	0.497	0.002	0.412
Major depressive	Alcohol	3	0.124	0.039	0.005	0.002	0.496	0.002	0.002
Major depressive	Alcohol	4	0.123	0.039	0.005	0.002	0.497	0.002	0.002
Major depressive	Calcium	0	-1.280	0.797	-0.003	0.002	0.340	0.002	0.108
Major depressive	Calcium	1	-1.113	0.797	-0.003	0.002	0.340	0.002	0.162
Major depressive	Calcium	2	-0.667	0.796	-0.002	0.002	0.340	0.002	0.402
Major depressive	Calcium	3	-0.807	0.793	-0.002	0.002	0.340	0.002	0.309
Major depressive	Calcium	4	-0.846	0.790	-0.002	0.002	0.339	0.002	0.284
Major depressive	Carbohydrate	0	-0.470	0.183	-0.005	0.002	0.409	0.002	0.010
Major depressive	Carbohydrate	1	-0.358	0.183	-0.004	0.002	0.410	0.002	0.050
Major depressive	Carbohydrate	2	-0.292	0.183	-0.003	0.002	0.410	0.002	0.110
Major depressive	Carbohydrate	3	-0.365	0.181	-0.004	0.002	0.409	0.002	0.043
Major depressive	Carbohydrate	4	-0.376	0.180	-0.004	0.002	0.409	0.002	0.036
Major depressive	Carotene	0	-10.454	5.661	-0.004	0.002	0.255	0.003	0.065
Major depressive	Carotene	1	-13.888	5.597	-0.005	0.002	0.260	0.003	0.013
Major depressive	Carotene	2	-10.846	5.592	-0.004	0.002	0.260	0.003	0.053
Major depressive	Carotene	3	-8.288	5.592	-0.003	0.002	0.260	0.003	0.138
Major depressive	Carotene	4	-8.436	5.543	-0.003	0.002	0.257	0.003	0.128
Major depressive	Fibre	0	-0.019	0.015	-0.003	0.002	0.436	0.002	0.207
Major depressive	Fibre	1	-0.026	0.015	-0.004	0.002	0.439	0.002	0.081
Major depressive	Fibre	2	-0.012	0.015	-0.002	0.002	0.439	0.002	0.403
Major depressive	Fibre	3	-0.008	0.015	-0.001	0.002	0.439	0.002	0.608
Major depressive	Fibre	4	-0.008	0.015	-0.001	0.002	0.436	0.002	0.583
Major depressive	Fat	0	-0.185	0.065	-0.006	0.002	0.335	0.002	0.004
Major depressive	Fat	1	-0.116	0.064	-0.004	0.002	0.341	0.002	0.070

Major depressive	Fat	2	-0.105	0.064	-0.003	0.002	0.341	0.002	0.102
Major depressive	Fat	3	-0.136	0.064	-0.004	0.002	0.341	0.002	0.034
Major depressive	Fat	4	-0.143	0.064	-0.005	0.002	0.341	0.002	0.025
Major depressive	Folate	0	-0.517	0.250	-0.004	0.002	0.406	0.002	0.039
Major depressive	Folate	1	-0.558	0.249	-0.005	0.002	0.407	0.002	0.025
Major depressive	Folate	2	-0.349	0.248	-0.003	0.002	0.407	0.002	0.161
Major depressive	Folate	3	-0.247	0.248	-0.002	0.002	0.407	0.002	0.320
Major depressive	Folate	4	-0.256	0.246	-0.002	0.002	0.405	0.002	0.299
Major depressive	Food weight	0	3.157	1.795	0.004	0.002	0.537	0.001	0.079
Major depressive	Food weight	1	2.602	1.793	0.003	0.002	0.536	0.001	0.147
Major depressive	Food weight	2	3.708	1.792	0.005	0.002	0.536	0.001	0.039
Major depressive	Food weight	3	4.088	1.775	0.005	0.002	0.536	0.001	0.021
Major depressive	Food weight	4	4.114	1.751	0.005	0.002	0.534	0.001	0.019
Major depressive	Iron	0	-0.039	0.010	-0.008	0.002	0.369	0.002	0.000
Major depressive	Iron	1	-0.038	0.010	-0.008	0.002	0.369	0.002	0.000
Major depressive	Iron	2	-0.024	0.010	-0.005	0.002	0.369	0.002	0.012
Major depressive	Iron	3	-0.014	0.010	-0.003	0.002	0.369	0.002	0.133
Major depressive	Iron	4	-0.015	0.009	-0.003	0.002	0.369	0.002	0.119
Major depressive	Protein	0	-0.070	0.054	-0.003	0.002	0.292	0.002	0.196
Major depressive	Protein	1	-0.036	0.054	-0.001	0.002	0.292	0.002	0.497
Major depressive	Protein	2	-0.015	0.054	-0.001	0.002	0.292	0.002	0.781
Major depressive	Protein	3	-0.017	0.054	-0.001	0.002	0.292	0.002	0.754
Major depressive	Protein	4	-0.021	0.054	-0.001	0.002	0.292	0.002	0.700
Major depressive	Vitamin B12	0	-0.020	0.009	-0.004	0.002	0.157	0.003	0.024
Major depressive	Vitamin B12	1	-0.016	0.009	-0.003	0.002	0.157	0.003	0.082
Major depressive	Vitamin B12	2	-0.009	0.009	-0.002	0.002	0.157	0.003	0.296
Major depressive	Vitamin B12	3	-0.006	0.009	-0.001	0.002	0.157	0.003	0.505
Major depressive	Vitamin B12	4	-0.006	0.009	-0.001	0.002	0.157	0.003	0.490
Major depressive	Vitamin B6	0	-0.004	0.002	-0.005	0.002	0.339	0.002	0.018
Major depressive	Vitamin B6	1	-0.004	0.002	-0.004	0.002	0.340	0.002	0.030
Major depressive	Vitamin B6	2	-0.003	0.002	-0.004	0.002	0.340	0.002	0.080
Major depressive	Vitamin B6	3	-0.002	0.002	-0.003	0.002	0.340	0.002	0.151
Major depressive	Vitamin B6	4	-0.002	0.002	-0.003	0.002	0.340	0.002	0.142
Major depressive	Vitamin C	0	-1.131	0.234	-0.010	0.002	0.396	0.002	0.000
Major depressive	Vitamin C	1	-1.187	0.233	-0.011	0.002	0.400	0.002	0.000
Major depressive	Vitamin C	2	-0.863	0.231	-0.008	0.002	0.399	0.002	0.000
Major depressive	Vitamin C	3	-0.693	0.231	-0.006	0.002	0.399	0.002	0.003
Major depressive	Vitamin C	4	-0.689	0.229	-0.006	0.002	0.397	0.002	0.003
Major depressive	Vitamin D	0	-0.007	0.006	-0.002	0.002	0.127	0.003	0.236
Major depressive	Vitamin D	1	-0.006	0.006	-0.002	0.002	0.127	0.003	0.308
Major depressive	Vitamin D	2	-0.003	0.006	-0.001	0.002	0.127	0.003	0.625

Major depressive	Vitamin D	3	-0.001	0.006	0.000	0.002	0.127	0.003	0.880
Major depressive	Vitamin D	4	-0.001	0.006	0.000	0.002	0.127	0.003	0.866
Major depressive	Vitamin E	0	-0.034	0.010	-0.007	0.002	0.296	0.002	0.001
Major depressive	Vitamin E	1	-0.036	0.010	-0.008	0.002	0.296	0.002	0.000
Major depressive	Vitamin E	2	-0.025	0.010	-0.005	0.002	0.296	0.002	0.010
Major depressive	Vitamin E	3	-0.022	0.010	-0.005	0.002	0.296	0.002	0.025
Major depressive	Vitamin E	4	-0.022	0.010	-0.005	0.002	0.295	0.002	0.020
OCD	Alcohol	0	-0.018	0.049	-0.001	0.002	0.479	0.002	0.716
OCD	Alcohol	1	-0.024	0.048	-0.001	0.002	0.496	0.002	0.622
OCD	Alcohol	2	-0.035	0.048	-0.002	0.002	0.497	0.002	0.466
OCD	Alcohol	3	-0.044	0.039	-0.002	0.002	0.496	0.002	0.269
OCD	Alcohol	4	-0.043	0.039	-0.002	0.002	0.497	0.002	0.281
OCD	Calcium	0	2.264	0.801	0.006	0.002	0.340	0.002	0.005
OCD	Calcium	1	2.288	0.801	0.006	0.002	0.340	0.002	0.004
OCD	Calcium	2	2.010	0.800	0.005	0.002	0.340	0.002	0.012
OCD	Calcium	3	1.978	0.796	0.005	0.002	0.340	0.002	0.013
OCD	Calcium	4	2.026	0.793	0.005	0.002	0.339	0.002	0.011
OCD	Carbohydrate	0	0.345	0.184	0.004	0.002	0.409	0.002	0.061
OCD	Carbohydrate	1	0.352	0.184	0.004	0.002	0.410	0.002	0.055
OCD	Carbohydrate	2	0.299	0.184	0.003	0.002	0.410	0.002	0.103
OCD	Carbohydrate	3	0.288	0.181	0.003	0.002	0.409	0.002	0.113
OCD	Carbohydrate	4	0.293	0.181	0.003	0.002	0.409	0.002	0.105
OCD	Carotene	0	8.227	5.693	0.003	0.002	0.255	0.003	0.148
OCD	Carotene	1	8.579	5.626	0.003	0.002	0.260	0.003	0.127
OCD	Carotene	2	6.230	5.620	0.002	0.002	0.260	0.003	0.268
OCD	Carotene	3	6.172	5.615	0.002	0.002	0.260	0.003	0.272
OCD	Carotene	4	6.671	5.566	0.002	0.002	0.257	0.003	0.231
OCD	Fibre	0	0.059	0.015	0.008	0.002	0.436	0.002	0.000
OCD	Fibre	1	0.060	0.015	0.009	0.002	0.439	0.002	0.000
OCD	Fibre	2	0.051	0.015	0.007	0.002	0.439	0.002	0.001
OCD	Fibre	3	0.050	0.015	0.007	0.002	0.439	0.002	0.001
OCD	Fibre	4	0.051	0.015	0.007	0.002	0.436	0.002	0.001
OCD	Fat	0	0.068	0.065	0.002	0.002	0.335	0.002	0.297
OCD	Fat	1	0.067	0.065	0.002	0.002	0.341	0.002	0.297
OCD	Fat	2	0.054	0.065	0.002	0.002	0.341	0.002	0.403
OCD	Fat	3	0.053	0.064	0.002	0.002	0.341	0.002	0.411
OCD	Fat	4	0.056	0.064	0.002	0.002	0.341	0.002	0.385
OCD	Folate	0	0.567	0.252	0.005	0.002	0.406	0.002	0.024
OCD	Folate	1	0.581	0.250	0.005	0.002	0.407	0.002	0.020
OCD	Folate	2	0.452	0.250	0.004	0.002	0.407	0.002	0.071
OCD	Folate	3	0.444	0.249	0.004	0.002	0.407	0.002	0.075

OCD	Folate	4	0.467	0.247	0.004	0.002	0.405	0.002	0.059
OCD	Food weight	0	1.829	1.805	0.002	0.002	0.537	0.001	0.311
OCD	Food weight	1	1.819	1.804	0.002	0.002	0.536	0.001	0.313
OCD	Food weight	2	1.111	1.801	0.001	0.002	0.536	0.001	0.537
OCD	Food weight	3	1.120	1.783	0.001	0.002	0.536	0.001	0.530
OCD	Food weight	4	1.256	1.759	0.002	0.002	0.534	0.001	0.475
OCD	Iron	0	0.031	0.010	0.007	0.002	0.369	0.002	0.001
OCD	Iron	1	0.031	0.010	0.007	0.002	0.369	0.002	0.001
OCD	Iron	2	0.021	0.010	0.005	0.002	0.369	0.002	0.028
OCD	Iron	3	0.020	0.010	0.004	0.002	0.369	0.002	0.036
OCD	Iron	4	0.021	0.010	0.004	0.002	0.369	0.002	0.029
OCD	Protein	0	-0.009	0.054	0.000	0.002	0.292	0.002	0.864
OCD	Protein	1	-0.008	0.054	0.000	0.002	0.292	0.002	0.889
OCD	Protein	2	-0.011	0.054	0.000	0.002	0.292	0.002	0.843
OCD	Protein	3	-0.010	0.054	0.000	0.002	0.292	0.002	0.847
OCD	Protein	4	-0.007	0.054	0.000	0.002	0.292	0.002	0.895
OCD	Vitamin B12	0	0.013	0.009	0.003	0.002	0.157	0.003	0.136
OCD	Vitamin B12	1	0.014	0.009	0.003	0.002	0.157	0.003	0.123
OCD	Vitamin B12	2	0.010	0.009	0.002	0.002	0.157	0.003	0.246
OCD	Vitamin B12	3	0.011	0.009	0.002	0.002	0.157	0.003	0.240
OCD	Vitamin B12	4	0.011	0.009	0.002	0.002	0.157	0.003	0.217
OCD	Vitamin B6	0	0.001	0.002	0.001	0.002	0.339	0.002	0.607
OCD	Vitamin B6	1	0.001	0.002	0.001	0.002	0.340	0.002	0.557
OCD	Vitamin B6	2	0.001	0.002	0.002	0.002	0.340	0.002	0.431
OCD	Vitamin B6	3	0.001	0.002	0.002	0.002	0.340	0.002	0.443
OCD	Vitamin B6	4	0.001	0.002	0.002	0.002	0.340	0.002	0.395
OCD	Vitamin C	0	0.084	0.235	0.001	0.002	0.396	0.002	0.722
OCD	Vitamin C	1	0.097	0.234	0.001	0.002	0.400	0.002	0.677
OCD	Vitamin C	2	-0.132	0.232	-0.001	0.002	0.399	0.002	0.571
OCD	Vitamin C	3	-0.134	0.232	-0.001	0.002	0.399	0.002	0.562
OCD	Vitamin C	4	-0.113	0.230	-0.001	0.002	0.397	0.002	0.624
OCD	Vitamin D	0	0.014	0.006	0.004	0.002	0.127	0.003	0.018
OCD	Vitamin D	1	0.014	0.006	0.005	0.002	0.127	0.003	0.016
OCD	Vitamin D	2	0.013	0.006	0.004	0.002	0.127	0.003	0.037
OCD	Vitamin D	3	0.013	0.006	0.004	0.002	0.127	0.003	0.037
OCD	Vitamin D	4	0.013	0.006	0.004	0.002	0.127	0.003	0.033
OCD	Vitamin E	0	0.032	0.010	0.007	0.002	0.296	0.002	0.001
OCD	Vitamin E	1	0.032	0.010	0.007	0.002	0.296	0.002	0.001
OCD	Vitamin E	2	0.023	0.010	0.005	0.002	0.296	0.002	0.016
OCD	Vitamin E	3	0.023	0.010	0.005	0.002	0.296	0.002	0.019
OCD	Vitamin E	4	0.024	0.010	0.005	0.002	0.295	0.002	0.014

Persistent thinne Alcohol	0	-0.101	0.057	-0.004	0.003	0.479	0.002	0.077
Persistent thinne Alcohol	1	-0.102	0.056	-0.005	0.003	0.496	0.002	0.071
Persistent thinne Alcohol	2	-0.104	0.056	-0.005	0.003	0.497	0.002	0.064
Persistent thinne Alcohol	3	-0.151	0.046	-0.007	0.002	0.496	0.002	0.001
Persistent thinne Alcohol	4	-0.150	0.046	-0.007	0.002	0.497	0.002	0.001
Persistent thinne Calcium	0	-0.586	0.942	-0.002	0.002	0.340	0.002	0.534
Persistent thinne Calcium	1	-0.649	0.942	-0.002	0.002	0.340	0.002	0.491
Persistent thinne Calcium	2	-0.616	0.941	-0.002	0.002	0.340	0.002	0.513
Persistent thinne Calcium	3	-0.536	0.936	-0.001	0.002	0.340	0.002	0.567
Persistent thinne Calcium	4	-0.510	0.932	-0.001	0.002	0.339	0.002	0.585
Persistent thinne Carbohydrate	0	0.267	0.216	0.003	0.003	0.409	0.002	0.217
Persistent thinne Carbohydrate	1	0.229	0.216	0.003	0.003	0.410	0.002	0.289
Persistent thinne Carbohydrate	2	0.233	0.216	0.003	0.003	0.410	0.002	0.281
Persistent thinne Carbohydrate	3	0.267	0.213	0.003	0.002	0.409	0.002	0.210
Persistent thinne Carbohydrate	4	0.273	0.212	0.003	0.002	0.409	0.002	0.199
Persistent thinne Carotene	0	-1.856	6.695	-0.001	0.002	0.255	0.003	0.782
Persistent thinne Carotene	1	-0.994	6.617	0.000	0.002	0.260	0.003	0.881
Persistent thinne Carotene	2	-0.849	6.608	0.000	0.002	0.260	0.003	0.898
Persistent thinne Carotene	3	-0.853	6.602	0.000	0.002	0.260	0.003	0.897
Persistent thinne Carotene	4	-0.397	6.544	0.000	0.002	0.257	0.003	0.952
Persistent thinne Fibre	0	0.002	0.018	0.000	0.003	0.436	0.002	0.921
Persistent thinne Fibre	1	0.004	0.018	0.001	0.003	0.439	0.002	0.843
Persistent thinne Fibre	2	0.004	0.018	0.001	0.003	0.439	0.002	0.821
Persistent thinne Fibre	3	0.006	0.017	0.001	0.003	0.439	0.002	0.753
Persistent thinne Fibre	4	0.007	0.017	0.001	0.003	0.436	0.002	0.706
Persistent thinne Fat	0	0.163	0.077	0.005	0.002	0.335	0.002	0.033
Persistent thinne Fat	1	0.142	0.076	0.005	0.002	0.341	0.002	0.061
Persistent thinne Fat	2	0.140	0.076	0.004	0.002	0.341	0.002	0.065
Persistent thinne Fat	3	0.141	0.076	0.004	0.002	0.341	0.002	0.063
Persistent thinne Fat	4	0.139	0.076	0.004	0.002	0.341	0.002	0.065
Persistent thinne Folate	0	0.607	0.296	0.005	0.003	0.406	0.002	0.040
Persistent thinne Folate	1	0.613	0.294	0.005	0.003	0.407	0.002	0.037
Persistent thinne Folate	2	0.624	0.294	0.005	0.003	0.407	0.002	0.034
Persistent thinne Folate	3	0.619	0.293	0.005	0.003	0.407	0.002	0.035
Persistent thinne Folate	4	0.634	0.291	0.005	0.003	0.405	0.002	0.029
Persistent thinne Food weight	0	-8.612	2.123	-0.011	0.003	0.537	0.001	0.000
Persistent thinne Food weight	1	-8.431	2.121	-0.010	0.003	0.536	0.001	0.000
Persistent thinne Food weight	2	-8.391	2.117	-0.010	0.003	0.536	0.001	0.000
Persistent thinne Food weight	3	-8.740	2.096	-0.011	0.003	0.536	0.001	0.000
Persistent thinne Food weight	4	-8.567	2.067	-0.011	0.003	0.534	0.001	0.000
Persistent thinne Iron	0	0.016	0.011	0.003	0.002	0.369	0.002	0.169

Persistent thinne	Iron	1	0.015	0.011	0.003	0.002	0.369	0.002	0.184
Persistent thinne	Iron	2	0.015	0.011	0.003	0.002	0.369	0.002	0.179
Persistent thinne	Iron	3	0.013	0.011	0.003	0.002	0.369	0.002	0.263
Persistent thinne	Iron	4	0.013	0.011	0.003	0.002	0.369	0.002	0.248
Persistent thinne	Protein	0	0.058	0.064	0.002	0.002	0.292	0.002	0.361
Persistent thinne	Protein	1	0.047	0.064	0.002	0.002	0.292	0.002	0.457
Persistent thinne	Protein	2	0.050	0.064	0.002	0.002	0.292	0.002	0.434
Persistent thinne	Protein	3	0.049	0.063	0.002	0.002	0.292	0.002	0.442
Persistent thinne	Protein	4	0.050	0.063	0.002	0.002	0.292	0.002	0.434
Persistent thinne	Vitamin B12	0	0.005	0.011	0.001	0.002	0.157	0.003	0.614
Persistent thinne	Vitamin B12	1	0.004	0.011	0.001	0.002	0.157	0.003	0.723
Persistent thinne	Vitamin B12	2	0.004	0.011	0.001	0.002	0.157	0.003	0.710
Persistent thinne	Vitamin B12	3	0.003	0.011	0.001	0.002	0.157	0.003	0.765
Persistent thinne	Vitamin B12	4	0.003	0.011	0.001	0.002	0.157	0.003	0.750
Persistent thinne	Vitamin B6	0	0.001	0.002	0.001	0.002	0.339	0.002	0.744
Persistent thinne	Vitamin B6	1	0.001	0.002	0.001	0.002	0.340	0.002	0.803
Persistent thinne	Vitamin B6	2	0.001	0.002	0.001	0.002	0.340	0.002	0.737
Persistent thinne	Vitamin B6	3	0.001	0.002	0.001	0.002	0.340	0.002	0.771
Persistent thinne	Vitamin B6	4	0.001	0.002	0.001	0.002	0.340	0.002	0.737
Persistent thinne	Vitamin C	0	-0.466	0.277	-0.004	0.003	0.396	0.002	0.092
Persistent thinne	Vitamin C	1	-0.454	0.275	-0.004	0.003	0.400	0.002	0.099
Persistent thinne	Vitamin C	2	-0.446	0.273	-0.004	0.003	0.400	0.002	0.103
Persistent thinne	Vitamin C	3	-0.451	0.273	-0.004	0.003	0.399	0.002	0.098
Persistent thinne	Vitamin C	4	-0.429	0.270	-0.004	0.002	0.397	0.002	0.113
Persistent thinne	Vitamin D	0	0.008	0.007	0.002	0.002	0.127	0.003	0.286
Persistent thinne	Vitamin D	1	0.007	0.007	0.002	0.002	0.127	0.003	0.312
Persistent thinne	Vitamin D	2	0.007	0.007	0.002	0.002	0.127	0.003	0.308
Persistent thinne	Vitamin D	3	0.007	0.007	0.002	0.002	0.127	0.003	0.332
Persistent thinne	Vitamin D	4	0.007	0.007	0.002	0.002	0.127	0.003	0.321
Persistent thinne	Vitamin E	0	0.008	0.011	0.002	0.002	0.296	0.002	0.504
Persistent thinne	Vitamin E	1	0.008	0.011	0.002	0.002	0.296	0.002	0.468
Persistent thinne	Vitamin E	2	0.008	0.011	0.002	0.002	0.296	0.002	0.481
Persistent thinne	Vitamin E	3	0.008	0.011	0.002	0.002	0.296	0.002	0.470
Persistent thinne	Vitamin E	4	0.008	0.011	0.002	0.002	0.295	0.002	0.458
Schizophrenia	Alcohol	0	0.056	0.050	0.003	0.002	0.479	0.002	0.267
Schizophrenia	Alcohol	1	0.047	0.050	0.002	0.002	0.496	0.002	0.349
Schizophrenia	Alcohol	2	0.034	0.050	0.002	0.002	0.497	0.002	0.496
Schizophrenia	Alcohol	3	0.013	0.041	0.001	0.002	0.496	0.002	0.754
Schizophrenia	Alcohol	4	0.014	0.041	0.001	0.002	0.497	0.002	0.729
Schizophrenia	Calcium	0	5.683	0.831	0.015	0.002	0.340	0.002	0.000
Schizophrenia	Calcium	1	5.753	0.831	0.015	0.002	0.340	0.002	0.000

Schizophrenia	Calcium	2	5.755	0.830	0.015	0.002	0.340	0.002	0.000
Schizophrenia	Calcium	3	5.868	0.826	0.015	0.002	0.340	0.002	0.000
Schizophrenia	Calcium	4	5.768	0.823	0.015	0.002	0.339	0.002	0.000
Schizophrenia	Carbohydrate	0	0.969	0.191	0.011	0.002	0.409	0.002	0.000
Schizophrenia	Carbohydrate	1	1.001	0.190	0.011	0.002	0.410	0.002	0.000
Schizophrenia	Carbohydrate	2	0.987	0.190	0.011	0.002	0.410	0.002	0.000
Schizophrenia	Carbohydrate	3	1.011	0.188	0.012	0.002	0.409	0.002	0.000
Schizophrenia	Carbohydrate	4	0.987	0.187	0.011	0.002	0.409	0.002	0.000
Schizophrenia	Carotene	0	38.618	5.905	0.014	0.002	0.255	0.003	0.000
Schizophrenia	Carotene	1	38.579	5.836	0.014	0.002	0.260	0.003	0.000
Schizophrenia	Carotene	2	37.522	5.831	0.013	0.002	0.260	0.003	0.000
Schizophrenia	Carotene	3	38.367	5.826	0.013	0.002	0.260	0.003	0.000
Schizophrenia	Carotene	4	37.520	5.775	0.013	0.002	0.257	0.003	0.000
Schizophrenia	Fibre	0	0.156	0.016	0.022	0.002	0.436	0.002	0.000
Schizophrenia	Fibre	1	0.157	0.016	0.022	0.002	0.439	0.002	0.000
Schizophrenia	Fibre	2	0.153	0.016	0.022	0.002	0.439	0.002	0.000
Schizophrenia	Fibre	3	0.159	0.015	0.023	0.002	0.439	0.002	0.000
Schizophrenia	Fibre	4	0.156	0.015	0.022	0.002	0.436	0.002	0.000
Schizophrenia	Fat	0	0.376	0.068	0.012	0.002	0.335	0.002	0.000
Schizophrenia	Fat	1	0.386	0.067	0.012	0.002	0.341	0.002	0.000
Schizophrenia	Fat	2	0.374	0.067	0.012	0.002	0.341	0.002	0.000
Schizophrenia	Fat	3	0.366	0.067	0.012	0.002	0.341	0.002	0.000
Schizophrenia	Fat	4	0.365	0.067	0.012	0.002	0.341	0.002	0.000
Schizophrenia	Folate	0	1.857	0.261	0.016	0.002	0.406	0.002	0.000
Schizophrenia	Folate	1	1.875	0.260	0.016	0.002	0.407	0.002	0.000
Schizophrenia	Folate	2	1.861	0.259	0.016	0.002	0.407	0.002	0.000
Schizophrenia	Folate	3	1.911	0.259	0.016	0.002	0.407	0.002	0.000
Schizophrenia	Folate	4	1.876	0.257	0.016	0.002	0.405	0.002	0.000
Schizophrenia	Food weight	0	14.306	1.872	0.018	0.002	0.537	0.001	0.000
Schizophrenia	Food weight	1	14.183	1.870	0.018	0.002	0.536	0.001	0.000
Schizophrenia	Food weight	2	13.917	1.868	0.017	0.002	0.536	0.001	0.000
Schizophrenia	Food weight	3	13.683	1.850	0.017	0.002	0.536	0.001	0.000
Schizophrenia	Food weight	4	13.317	1.824	0.017	0.002	0.534	0.001	0.000
Schizophrenia	Iron	0	0.068	0.010	0.015	0.002	0.369	0.002	0.000
Schizophrenia	Iron	1	0.068	0.010	0.015	0.002	0.369	0.002	0.000
Schizophrenia	Iron	2	0.064	0.010	0.014	0.002	0.369	0.002	0.000
Schizophrenia	Iron	3	0.066	0.010	0.014	0.002	0.369	0.002	0.000
Schizophrenia	Iron	4	0.066	0.010	0.014	0.002	0.369	0.002	0.000
Schizophrenia	Protein	0	0.307	0.056	0.011	0.002	0.292	0.002	0.000
Schizophrenia	Protein	1	0.315	0.056	0.012	0.002	0.292	0.002	0.000
Schizophrenia	Protein	2	0.330	0.056	0.012	0.002	0.292	0.002	0.000

Schizophrenia	Protein	3	0.336	0.056	0.012	0.002	0.292	0.002	0.000
Schizophrenia	Protein	4	0.332	0.056	0.012	0.002	0.292	0.002	0.000
Schizophrenia	Vitamin B12	0	0.043	0.009	0.009	0.002	0.157	0.003	0.000
Schizophrenia	Vitamin B12	1	0.045	0.009	0.009	0.002	0.157	0.003	0.000
Schizophrenia	Vitamin B12	2	0.045	0.009	0.009	0.002	0.157	0.003	0.000
Schizophrenia	Vitamin B12	3	0.046	0.009	0.010	0.002	0.157	0.003	0.000
Schizophrenia	Vitamin B12	4	0.045	0.009	0.010	0.002	0.157	0.003	0.000
Schizophrenia	Vitamin B6	0	0.004	0.002	0.005	0.002	0.339	0.002	0.012
Schizophrenia	Vitamin B6	1	0.005	0.002	0.006	0.002	0.340	0.002	0.007
Schizophrenia	Vitamin B6	2	0.006	0.002	0.007	0.002	0.340	0.002	0.001
Schizophrenia	Vitamin B6	3	0.006	0.002	0.008	0.002	0.340	0.002	0.000
Schizophrenia	Vitamin B6	4	0.006	0.002	0.008	0.002	0.340	0.002	0.000
Schizophrenia	Vitamin C	0	0.516	0.244	0.005	0.002	0.396	0.002	0.034
Schizophrenia	Vitamin C	1	0.530	0.243	0.005	0.002	0.400	0.002	0.029
Schizophrenia	Vitamin C	2	0.449	0.241	0.004	0.002	0.400	0.002	0.062
Schizophrenia	Vitamin C	3	0.512	0.241	0.005	0.002	0.399	0.002	0.033
Schizophrenia	Vitamin C	4	0.475	0.239	0.004	0.002	0.397	0.002	0.047
Schizophrenia	Vitamin D	0	0.027	0.006	0.009	0.002	0.127	0.003	0.000
Schizophrenia	Vitamin D	1	0.028	0.006	0.009	0.002	0.127	0.003	0.000
Schizophrenia	Vitamin D	2	0.027	0.006	0.009	0.002	0.127	0.003	0.000
Schizophrenia	Vitamin D	3	0.028	0.006	0.009	0.002	0.127	0.003	0.000
Schizophrenia	Vitamin D	4	0.028	0.006	0.009	0.002	0.127	0.003	0.000
Schizophrenia	Vitamin E	0	0.061	0.010	0.013	0.002	0.296	0.002	0.000
Schizophrenia	Vitamin E	1	0.061	0.010	0.013	0.002	0.296	0.002	0.000
Schizophrenia	Vitamin E	2	0.056	0.010	0.012	0.002	0.296	0.002	0.000
Schizophrenia	Vitamin E	3	0.058	0.010	0.012	0.002	0.296	0.002	0.000
Schizophrenia	Vitamin E	4	0.057	0.010	0.012	0.002	0.295	0.002	0.000
