# Supplementary material for: Association between polygenic propensity for psychiatric disorders and nutrient intake
Source: Commun Biol. 2021 Aug 26;4:965. doi: 10.1038/s42003-021-02469-4 (PMC8390493; doi:10.1038/s42003-021-02469-4)
Supplement: Supplementary file 6 — Reporting Summary [file 42003_2021_2469_MOESM6_ESM.pdf]

nature research

Corresponding author(s): Gerome Breen

Last updated by author(s): May 27, 2021

Reporting Summary

Nature Research wishes to improve the reproducibility of the work that we publish. This form provides structure for consistency and transparency in reporting. For further information on Nature Research policies, see our Editorial Policies and the Editorial Policy Checklist.

Statistics

For all statistical analyses, confirm that the following items are present in the figure legend, table legend, main text, or Methods section.

n/a	Confirmed	
☐	☒	The exact sample size (*n*) for each experimental group/condition, given as a discrete number and unit of measurement
☐	☒	A statement on whether measurements were taken from distinct samples or whether the same sample was measured repeatedly
☒	☐	The statistical test(s) used AND whether they are one- or two-sided<br>*Only common tests should be described solely by name; describe more complex techniques in the Methods section.*
☐	☒	A description of all covariates tested
☐	☒	A description of any assumptions or corrections, such as tests of normality and adjustment for multiple comparisons
☐	☒	A full description of the statistical parameters including central tendency (e.g. means) or other basic estimates (e.g. regression coefficient) AND variation (e.g. standard deviation) or associated estimates of uncertainty (e.g. confidence intervals)
☒	☐	For null hypothesis testing, the test statistic (e.g. *F*, *t*, *r*) with confidence intervals, effect sizes, degrees of freedom and *P* value noted<br>*Give P values as exact values whenever suitable.*
☒	☐	For Bayesian analysis, information on the choice of priors and Markov chain Monte Carlo settings
☒	☐	For hierarchical and complex designs, identification of the appropriate level for tests and full reporting of outcomes
☐	☒	Estimates of effect sizes (e.g. Cohen's *d*, Pearson's *r*), indicating how they were calculated

Our web collection on statistics for biologists contains articles on many of the points above.

Software and code

Policy information about availability of computer code

Data collection	No software was used
Data analysis	Analysis code can be accessed on https://github.com/AvinaHunjan/scripts. Software can be accessed for PRSice, at https://choishingwan.github.io/PRSice/.

For manuscripts utilizing custom algorithms or software that are central to the research but not yet described in published literature, software must be made available to editors and reviewers. We strongly encourage code deposition in a community repository (e.g. GitHub). See the Nature Research guidelines for submitting code & software for further information.

Data

Policy information about availability of data

All manuscripts must include a data availability statement. This statement should provide the following information, where applicable:

- Accession codes, unique identifiers, or web links for publicly available datasets
- A list of figures that have associated raw data
- A description of any restrictions on data availability

Authors had full access to the data supporting the findings of this study. UK Biobank is an open access resource. Data are available to bona fide scientists, undertaking health-related research that is in the public good. All individual-level data from UK Biobank can be accessed by applying to the UK Biobank Central Access Committee (http://www.ukbiobank.ac.uk/register-apply/). The datasets used in this study are available from A. K. Hunjan on reasonable request

Field-specific reporting

Please select the one below that is the best fit for your research. If you are not sure, read the appropriate sections before making your selection.

☐ Life sciences ☒ Behavioural & social sciences ☐ Ecological, evolutionary & environmental sciences

For a reference copy of the document with all sections, see nature.com/documents/nr-reporting-summary-flat.pdf

Behavioural & social sciences study design

All studies must disclose on these points even when the disclosure is negative.

Study description	Quantitative longitudinal study
Research sample	This study used data from the UK Biobank, a large-scale biomedical database and research resource. Since 2006, UK Biobank has collected biological and medical data on approximately 500,000 individuals, aged between 40 and 69 years old and living in the UK, as part of a large-scale prospective study.
Sampling strategy	UK Biobank investigators sent postal invitations to 9,238,453 individuals registered with the UK's National Health Service who lived within approximately 25 miles (40 km) of one of 22 assessment centers located throughout England, Wales, and Scotland (i.e., convenience based sampling). Overall, 503,317 participants consented to join the study cohort and visited an assessment center between 2006 and 2010, resulting in a participation rate of 5.45% (thus, the sampling population is volunteer-based).
Data collection	Dietary behavior was assessed using a web-based 24-hour dietary assessment tool which asked about the frequency of consumption of common foods and drinks. Participants were asked whether what they ate and drank yesterday was typical (Data-Field 100020) and if they routinely followed a special diet. Responses were automatically coded to provide estimated daily nutrient intake. Participants were invited to complete the assessment on five occasions. In total, 211,036 participants completed the dietary assessment at least once.
Timing	Participants recruited between April 2009 and September 2010 completed the dietary assessment at the assessment centre. After the recruitment period closed, the dietary assessment was completed remotely on four separate occasions (cycle 1, February-April 2011; cycle 2, June-September 2011; cycle 3, October-December 2011; cycle 4, April-June 2012) by participants who provided an email address at recruitment.
Data exclusions	Pregnant females were removed as well as potential outliers in the dataset using lower and upper cutoff limits to each estimated nutrient. We included all time points each participant completed the assessment but restricted our analyses to individuals with genotype data who also had complete data on variables that may potentially influence nutrient intake: typicality and kind of diet followed, socioeconomic status, educational attainment physical activity, and medications and diagnoses that affect body composition. Our sample after exclusion consisted of 350,339 data entries for each estimated nutrient intake covering 163,619 participants (77.5% of the original sample).
Non-participation	When individuals were contacted by the UK Biobank project they were told "At some time in the future, participants might be re-contacted by UK Biobank and asked more questions, although giving such additional help would be entirely optional. Similarly, some participants might be asked in later years to attend another assessment visit (including questions, measurements and samples), although again attendance at such visits would be optional" (https://www.ukbiobank.ac.uk/media/ei3bagfb/participant_information_leaflet-baseline.pdf). Given that follow-up assessments were optional this explains why not all participants recruited by the UK Biobank project completed the diet assessment at all 5 time points. Nonetheless, the sample size used in our study was sufficiently large.
Randomization	Participants were not assigned to different groups

Reporting for specific materials, systems and methods

We require information from authors about some types of materials, experimental systems and methods used in many studies. Here, indicate whether each material, system or method listed is relevant to your study. If you are not sure if a list item applies to your research, read the appropriate section before selecting a response.

Materials & experimental systems

n/a	Involved in the study	
☒	☐	Antibodies
☒	☐	Eukaryotic cell lines
☒	☐	Palaeontology and archaeology
☒	☐	Animals and other organisms
☐	☒	Human research participants
☒	☐	Clinical data
☒	☐	Dual use research of concern

Methods

n/a	Involved in the study	
☒	☐	ChIP-seq
☒	☐	Flow cytometry
☒	☐	MRI-based neuroimaging

Human research participants

Policy information about studies involving human research participants

Population characteristics	See above
Recruitment	UK Biobank is not representative of the sampling population; there is evidence of a "healthy volunteer" selection bias. However, this does not impact our findings as we aimed to see how dietary intake in "healthy" individuals is influenced by genetic risk for a psychiatric disorder.
Ethics oversight	Protocol approval was not required. However, in the methods we state "We obtained approval for this research under an approved access request (application 23395) to UK Biobank. UK Biobank has approval from the North West Multi-centre Research Ethics Committee, which covers the UK, and the Patient Information Advisory Group for gaining access to information that would allow it to invite people to participate. Our use of the data was governed by the analysis plan in our access request and the terms of the material transfer agreement between KCL and UK Biobank. We assert that all procedures contributing to this work comply with the ethical standards of the relevant national and institutional committees on human experimentation and with the Helsinki Declaration of 1975, as revised in 2008."

Note that full information on the approval of the study protocol must also be provided in the manuscript.
